# Clinical aspects of impulsive-compulsive behaviors under dopaminergic treatment—A scoping review

**DOI:** 10.1177/10398562261439053

**Published:** 2026-03-28

**Authors:** Mirjam Wolfschlag, Darya Ståhl, Anders Håkansson, Per Odin

**Affiliations:** Clinical Addiction Research Unit, Psychiatry, Department of Clinical Sciences Lund, Faculty of Medicine, 5193 Lund University, Lund, Sweden; Region Skåne, Malmö Addiction Center, Skåne University Hospital, Malmö, Sweden; Unit for Biological and Precision Psychiatry, Psychiatry, Department of Clinical Sciences Lund, Faculty of Medicine, 5193 Lund University, Lund, Sweden; Clinical Addiction Research Unit, Psychiatry, Department of Clinical Sciences Lund, Faculty of Medicine, 5193 Lund University, Lund, Sweden; Region Skåne, Malmö Addiction Center, Skåne University Hospital, Malmö, Sweden; Division of Neurology, Department of Clinical Sciences Lund, Faculty of Medicine, 5193 Lund University, Lund, Sweden; Region Skåne, Section of Neurology, Department of Neurology, Rehabilitation Medicine, Memory and Geriatrics, Skåne University Hospital, Malmö, Sweden

**Keywords:** impulse control disorder, dopamine agonist, Parkinson’s disease, restless legs syndrome, pituitary disorder

## Abstract

**Objective:**

Dopaminergic medications are commonly prescribed for Parkinson’s disease (PD), restless legs syndrome, pituitary and psychotic disorders. Accumulating evidence indicates that dopaminergic treatment triggers impulsive-compulsive behaviors (ICBs), yet systematic synthesis of clinical evidence remains limited.

**Method:**

This scoping review summarizes original research directly clinically relevant to ICBs under dopaminergic treatment, selected through systematic searches of MEDLINE PubMed, Web of Science, Embase and PsycInfo.

**Results:**

Of 349 included studies, most focused on PD, with limited evidence from other populations. Reported ICB prevalences ranged between 15 and 35%. ICBs were associated with impaired daily functioning, depression, anxiety and significant family and financial burden. Younger age, male sex and dopamine agonist treatment, especially with pramipexole and ropinirole, were ICB risk factors. Management mainly involved tapering or discontinuing dopamine agonist treatment.

**Conclusion:**

ICBs in PD patients are well characterized, but under-recognition in health care and limited treatment strategies persist. Iatrogenic ICBs in other patient populations remain poorly studied.

## Introduction

In this review, “dopaminergic medications” refers to drugs with direct dopaminergic receptor activity, including dopamine agonists (e.g., pramipexole, ropinirole, rotigotine) and levodopa, that are used in Parkinson’s disease (PD),^1,2^ restless legs syndrome^3^ and prolactinomas/hyperprolactinemia.^1^ Additionally, the term includes antipsychotics with partial dopamine agonism (e.g., aripiprazole), used mainly in schizophrenia and bipolar disorder. These dopaminergic medications can induce impulsive-compulsive behaviors (ICBs) like pathological gambling, compulsive sexuality, shopping, and eating, but also compulsive behaviors like punding and dopamine dysregulation syndrome (compulsive overuse of dopaminergic medication despite harmful consequences).^4–10^

From a clinical perspective, ICBs under dopaminergic medication are highly relevant and can have tremendous psychological, social and economic consequences.^11,12^ ICBs affect not only patients’ quality of life but also burden families with powerlessness, violence, and financial hardship.^11,13,14^ ICBs are often reversible after treatment modification, although management may remain complex and requires multidisciplinary follow-up.^15,16^

While narrative reviews on ICBs in PD are available, a recent, systematic overview is lacking and evidence beyond PD populations remains limited. This scoping review summarizes original research on dopaminergic treatment-related ICBs, with focus on prevalence, impact, risk factors, comorbidities and management.

## Materials and methods

This review was conducted in the Covidence web tool^17^ according to PRISMA guidelines^18^ (Supplemental file S1), and the scoping design was chosen over a more systematic approach due to the broad research question and the substantial heterogeneity across study populations. Two reviewers applied independent search strategies to the databases MEDLINE PubMed, Web of Science, Embase and PsycInfo in April 2025 (Supplemental file S2). Included were original studies in any patient population published in English in any year with the exposure dopaminergic treatment and ICB as outcome. Excluded were animal studies, case reports, conference abstracts and studies focusing on neurobiological or psychological mechanisms only.

Title/abstract screening and full-text assessment were performed by two reviewers independently and discrepancies were resolved through consensus discussion. Data were extracted using a standardized form including study characteristics and outcomes. Findings were synthesized to identify consensus, major trends, key contributions and areas of controversy.

## Results

### Included studies

After full-text assessment, 349 original articles were selected for data extraction (Figure 1), published between 2003 and 2025. Lists of study characteristics and main findings are provided as Supplemental file S3 and S4. Throughout the results section, studies mentioned are referenced as article A1–A349 according to supplementary material. The vast majority of studies was performed in PD patients (*n* = 299, 86%), with additional studies in patients with pituitary disorders (*n* = 15, 4%), restless legs syndrome (*n* = 14, 4%) and psychotic disorders (*n* = 8, 2%).

**Figure 1.**
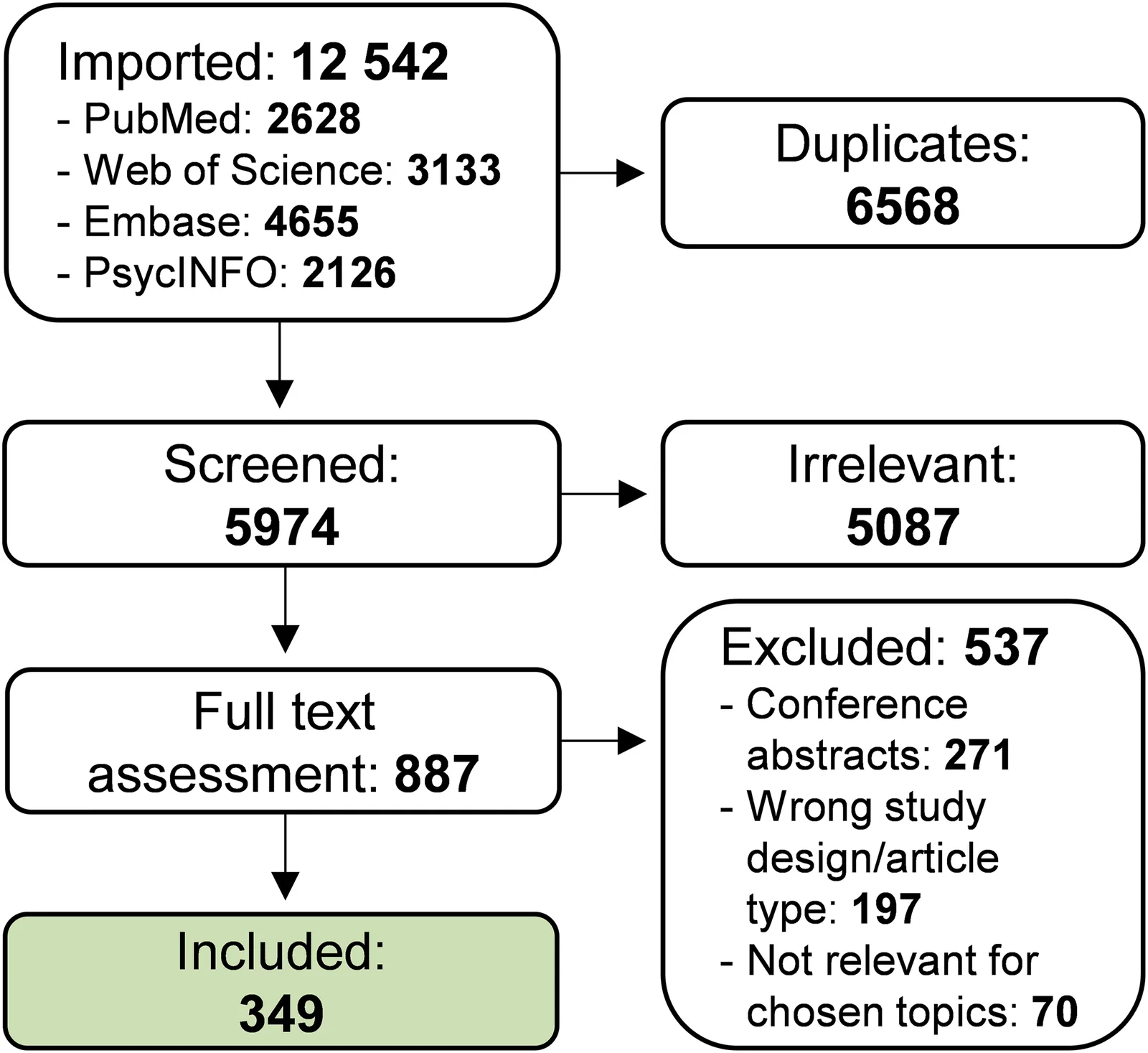
Flow chart of screening and inclusion process.

About three quarters of studies were conducted in Western regions (Figure 2), with the largest fractions in Europe (*n* = 184, 52%) and North America (*n* = 60, 18%). Especially under-represented were South American populations (*n* = 6, 2%).

**Figure 2.**
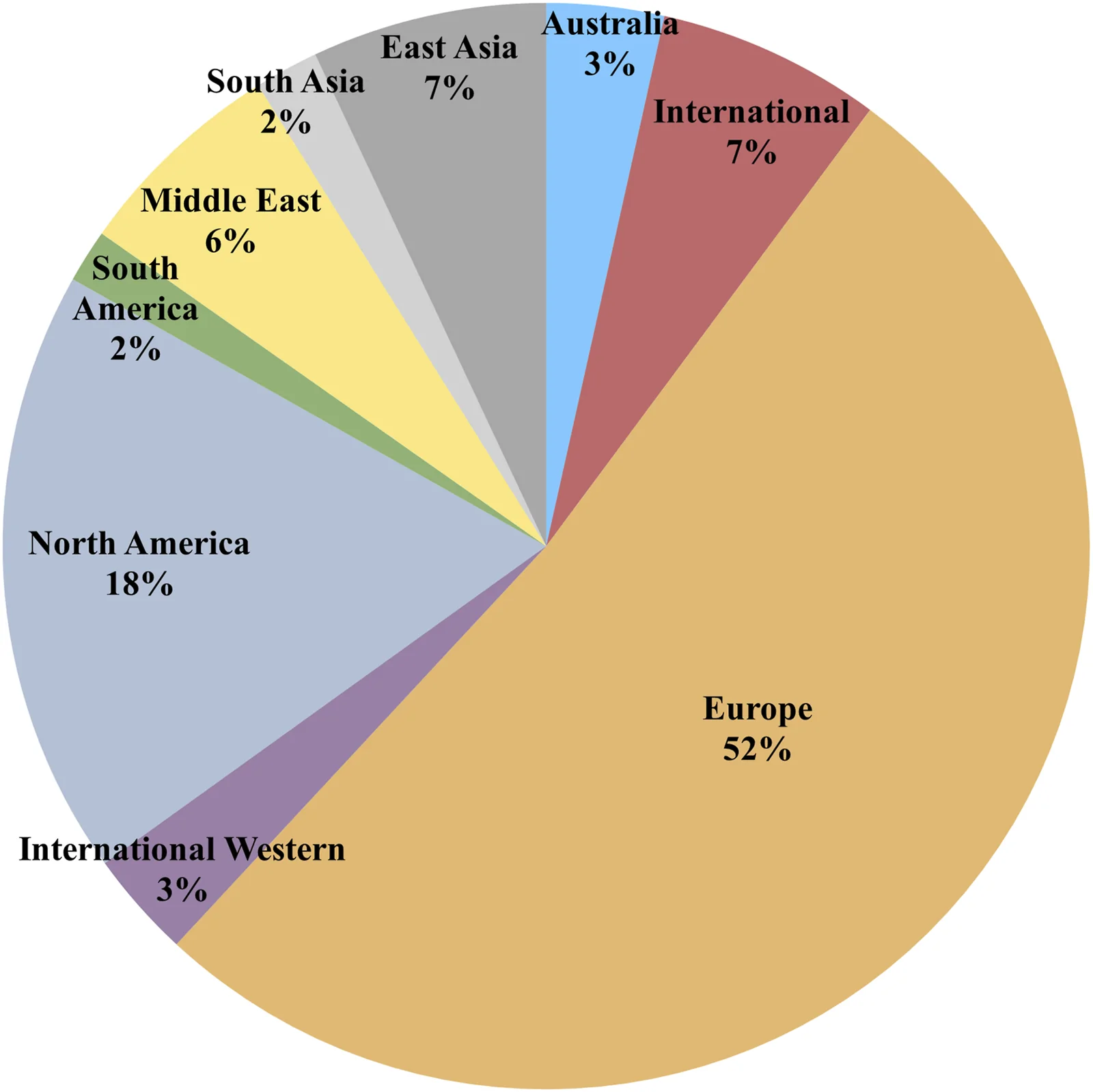
Regions of origin for included studies.

Evidence stems mainly from cross-sectional designs (*n* = 153, 44%), cohort (*n* = 92, 26%) and case-control studies (*n* = 44, 13%). The largest clinical studies in PD reach populations up to 3000 patients ^A10,A164,A309,A331,A332^, with one French cohort including as many as 12,000 patients ^A95^. In comparison, the largest non-PD population included 398 prolactinoma patients ^A97^.

### Prevalence

Prevalence estimates of ICBs, primarily based on cross-sectional studies of PD populations, vary widely across studies and regions, ranging from about 6% in East Asian cohorts ^A57,A96,A246^ to over 50% in European, South American and South Asian samples ^A4,A41,A203,A318,A319^. Most estimates fall between 15 and 35%, suggesting that ICBs are not equally common worldwide and that the population studied, assessment methods and diagnostic criteria used may underlie these differences. Among the most frequently reported ICBs were hypersexuality, pathological gambling and compulsive shopping, with median prevalence of 7.9%, 3.9% and 7.2%, respectively, and wide variability across studies (approximate ranges 1–42%, 0,5–35%, 0,5–30%).

Less studied than in PD patients, the prevalence of ICBs in restless legs syndrome varies between 7 and 40% ^A126,A118,A251,A278,A327^. Moreover, in patients with prolactinomas and other pituitary disorders the ICB frequency was reported to lie between 7 and 61% ^A20,A33,A52,A75,A79^. ICB prevalences under treatment with the antipsychotic aripiprazole have not been studied systematically, but the prevalence of pathological gambling in one cohort was about 6% ^A62^.

### Impact

ICBs developed under dopaminergic treatment negatively affected quality of life and health ^A5,A88,A179,A191,A246,A262^, daily functioning and work ^A45,A246^, sleep and daytime fatigue ^A159^. Multiple studies also report high rates of depression, anxiety and apathy, highlighting the effect on psychological health ^A67,A200,A274^.

ICBs are associated with psychological distress, anxiety, shame, reduced intimacy, anger and social withdrawal among relatives of affected patients ^A211,A304^. Furthermore, families experience severe economic consequences from excessive gambling or shopping, frequently bearing debts or managing patients’ finances ^A88,A159^. Notably, most relatives receive no warning about the risk of developing ICB under dopaminergic therapy and must themselves initiate contact for medical modification for the patient ^A211,A304^.

Patients tend to underreport ICBs: In a Swiss study ^A30^, relatives reported hypersexuality in 55% of patients, while only 17% of patients did. In a Swedish study ^A338^, just 49% disclosed behaviors to physicians, and in a Danish study only 5–26% informed healthcare providers or relatives ^A45^.

Some studies found no clear link between ICBs and quality of life, reporting mild symptoms ^A171^, while another suggested that depression, anxiety and apathy had a greater negative influence than ICB ^A67^.

### Risk factors

Regarding ICB risk factors, PD cohort studies have contributed with the strongest evidence, supported by a large number of cross-sectional studies and some case-control designs. In PD patients, there is a strong consensus for younger age at PD onset as ICB risk factor. Male patients are more vulnerable, especially for compulsive sexuality and pathological gambling, with some negative and contradictory findings ^A2,A74,A214^. As an exception, compulsive shopping and eating seem more common in women ^A84,A150,A319,A332^. In non-PD populations, a few studies suggest that females might be at higher risk for ICBs ^A157,A237,A327^.

Regarding pharmacotherapy, dopamine agonists are established high-risk agents for developing ICB in contrast to levodopa. Despite some studies showing no differences between agonist types ^A20,A63,A75,A327^, the D2/3 receptor agonists pramipexole and ropinirole have been identified to bear the highest ICB risk ^A63,A263,A296,A340^. MAO-B inhibitors are pointed out as safe in the ICB context with some exceptions ^A108,A223,A224,A337,A349^, while findings on amantadine treatment range from describing it as an additional risk factor ^A95,A148,A186,A284,A331^ to suggesting it as management strategy for ICBs ^A53,A102,A306^. In patients with psychotic disorders, aripiprazole has been pointed out as a strong ICB risk factor, particularly for gambling-related behaviors ^A62,A69,A103,A219^, but even other antipsychotics might have a relation to ICBs ^A345^.

Even though multiple studies showed a dose effect of dopamine agonist and total dopaminergic treatment on ICBs, there are many negative findings. Longer disease duration is commonly associated with developing ICB, yet the duration of dopaminergic therapy seems to have no such effect with some exceptions ^A46,A63,A100,A176,A204,A284^. Extended-release formulations have both been related to a lower ^A77,A263^ or equally high ICB risk ^A108,A120,A150^ compared to immediate-release designs.

### Comorbidity

Comorbidity findings were mainly derived from cross-sectional studies comparing PD patients with and without ICB, although some studies were descriptive ^A3,A44,A145,A177,A178^. In several studies, comparisons were also made with healthy control groups ^A13,A86,A87^. Depression and anxiety were common in PD patients with ICBs. One study found depression in 66% (22% severe) with pathologic gambling, binge-eating and punding particularly linked to depression ^A274^. Another study observed higher levels of depression, anxiety and symptoms of obsessive-compulsive disorders in patients with ICBs compared to controls ^A326^. While several studies identified depression or anxiety as risk factors for ICBs ^A4,A10,A15^, others found no association ^A34,A112^. Apathy, anhedonia and decreased motivation were also frequently associated symptoms of ICB ^A19,A272,A280^, as were other non-motor symptoms such as vivid dreams, hallucinations, mental fatigue and irritability ^A338^.

Cognitive findings in ICB patients vary: One study showed executive deficits in all ICB groups ^A321^, while others saw no cognitive differences ^A15,A34,A182^. REM sleep behavior disorder, excessive daytime sleepiness and autonomic dysfunction have been linked to ICB in several studies ^A342,A347^, though some results showed no association after adjustment ^A32^.

Regarding lifestyle factors, higher prevalences of smoking, caffeine and alcohol consumption, and unmarried status were noted in patients with iatrogenic ICBs ^A10,A29,A112^. Psychiatric history, including alcohol or substance use and family history of gambling, were more common in ICB patients ^A274,A337^. Interestingly, one study reported lower harmful alcohol use in PD patients with ICBs, suggesting ICBs may not follow a general dependence pattern ^A70^.

### Management

The major body of evidence for ICB management stems from cohort studies exploring longitudinal changes in dopaminergic treatment and from interventional designs including a small number of randomized controlled trials. The most common, often successful approach to manage any ICB in all patient populations is adapting the dopaminergic treatment, mainly by dopamine agonist tapering or discontinuation. In addition, advanced PD therapies can be an option, with some studies showing a clear reduction in ICBs after deep brain stimulation ^A6,A163,A184,A272^ and some a weaker or no effect ^A130,A224,A238,A286^. Even levodopa-carbidopa intestinal gel ^A51,A59,A190,A308^ and, to some degree, apomorphine infusion ^A22,A26,A212,A308^ have been proven helpful in mitigating ICBs in general. ICB pharmacotherapy can include antidepressants ^A196^, antipsychotics ^A290^ or other drugs ^A36,A174,A241^ with some beneficial effects. Addiction-oriented pharmacological approaches such as naltrexone, and rarely buprenorphine, have been explored, although evidence remains limited ^A241,A337^. There is also some evidence for the efficacy of counselling/psychotherapy ^A228,A231^, abstinence support groups ^A37,A196^, rehabilitation programs ^A170^ or transcranial stimulation ^A226^. However, both for ICB pharmacotherapy ^A17,A59,A292^ and other strategies ^A25,A306^, many studies fail to prove their efficacy in treating any ICB.

## Summary and future perspectives

Clinical knowledge about ICBs in PD has been accumulated over the past 20 years, enabling identification of vulnerable patients and adaptation of dopaminergic treatment. Prevalence estimates and proof for the severe impact of ICBs highlight the need to recognize them as a major contributor to the PD disease burden. While a basic understanding for high-dose D2/3 receptor agonists as the main ICB risk factor has been established, controversies remain regarding specific treatment strategies. Moreover, most studies were performed in Western populations and little is known about global ICB differences. Comorbid psychiatric conditions, including depression, anxiety and apathy, both predispose individuals to and result from ICBs, suggesting a bidirectional relationship that warrants further investigation. The functional impact of ICBs deserves greater attention as systematic assessment of caregiver burden remains rare. Aripiprazole has been associated with ICBs such as pathological gambling, which often improve after dose reduction or discontinuation ^A62,A89,A103^. As a limitation of this review, studies were not ranked according to evidence level and no formal risk-of-bias or quality assessment was performed.

Taken together, future studies in PD patients should focus on mapping the ICB effect of specific dopaminergic therapies and routes of administration, but also on providing evidence-based ICB interventions. Non-PD populations have barely been studied and systematic research is urgently needed. The transferability of results from PD populations is doubtful due to substantially different demographics, underlying disease pathologies and dopaminergic therapy approaches. Especially for aripiprazole, most evidence is anecdotal so far, and risk factors in different psychiatric populations should be explored to guide monitoring of ICB during antipsychotic treatment. Further, future research should systematically evaluate psychosocial consequences for patients and their relatives, essential to refine screening, prevention and intervention strategies.

## Supplemental material

Supplemental material - Clinical aspects of impulsive-compulsive behaviors under dopaminergic treatment – A scoping review Supplemental material for Clinical aspects of impulsive-compulsive behaviors under dopaminergic treatment—A scoping review by Mirjam Wolfschlag, Darya Ståhl, Anders Håkansson and Per Odin in Australasian Psychiatry

## Data Availability

All results extracted from original studies and data charted in this scoping review are provided as Supplementary file S3 and S4.*
