# Supplementary material for: Clinical aspects of impulsive-compulsive behaviors under dopaminergic treatment—A scoping review
Source: Australas Psychiatry. 2026 Mar 28;34(5):432–6. doi: 10.1177/10398562261439053 (PMC13669450; doi:10.1177/10398562261439053)
Supplement: Supplementary file 1 [file sj-zip-1-apy-10.1177_10398562261439053.zip › Supplementary file S2_Search strategies.pdf]

# Search strategies for electronic databases

## Reviewer 1

**PubMed**

"dopaminergic medication"[Title/Abstract] OR "dopamine replacement therapy"[Title/Abstract] OR "dopaminergic therapy"[Title/Abstract] OR "dopamine modulator"[Title/Abstract] OR "dopaminergic drug*"[Title/Abstract] OR "dopaminergic treatment"[Title/Abstract] OR "dopamine agonist"[Title/Abstract] OR parkinson*[Title/Abstract] OR RLS[Title/Abstract] OR restless legs[Title/Abstract] OR fibromyalgia[Title/Abstract] OR pituitary adenoma[Title/Abstract] OR hyperprolactinem*[Title/Abstract] OR aripiprazole[Title/Abstract] OR pramipexole[Title/Abstract] OR ropinirole[Title/Abstract] OR levodopa[Title/Abstract]

AND

"impulse control"[Title/Abstract] OR impulsiv*[Title/Abstract] OR compulsiv*[Title/Abstract] OR gambl*[Title/Abstract] OR hypersexual*[Title/Abstract] OR buying[Title/Abstract] OR buyer*[Title/Abstract] OR shopp*[Title/Abstract] OR pund*[Title/Abstract] OR hobbyism[Title/Abstract]

NOT

review[Title] OR metaanalysis[Title] OR meta-analysis[Title] OR "meta analysis"[Title] OR mouse[Title] OR mice[Title] OR rat[Title] OR rats[Title] OR hamster[Title] OR rodent*[Title] OR animal [Title] OR case [Title]

**Embase**

'dopaminergic medication':ab,ti OR 'dopamine replacement therapy':ab,ti OR 'dopaminergic therapy':ab,ti OR 'dopamine modulator':ab,ti OR 'dopaminergic drug*':ab,ti OR 'dopaminergic treatment':ab,ti OR 'dopamine agonist':ab,ti OR parkinson*:ab,ti OR rls:ab,ti OR 'restles legs':ab,ti OR fibromyalgia:ab,ti OR 'pituitary adenoma':ab,ti OR hyperprolactinem*:ab,ti OR aripiprazole:ab,ti OR pramipexole:ab,ti OR ropinirole:ab,ti OR levodopa:ab,ti

AND

'impulse control':ab,ti OR impulsiv*:ab,ti OR compulsiv*:ab,ti OR gambl*:ab,ti OR hypersexual*:ab,ti OR buying:ab,ti OR buyer*:ab,ti OR shopp*:ab,ti OR pund*:ab,ti OR hobbyism:ab,ti

NOT

'review':ti OR 'metaanalysis':ti OR 'meta-analysis':ti OR 'mouse':ti OR 'mice':ti OR 'rat':ti OR 'rats':ti OR 'hamster':ti OR 'rodent*':ti OR 'animal':ti OR 'case':ti) AND [humans]/lim AND [english]/lim AND [2000-2025]/py AND ('article'/it OR 'article in press'/it OR 'clinical trial'/it)

**PsycINFO**
XB ( 'impulse control' OR impulsiv* OR compulsiv* OR gambl* OR hypersexual* OR buying OR buyer* OR shopp* OR pund* OR hobbyism ) AND XB ( 'dopaminergic medication' OR 'dopamine replacement therapy' OR 'dopaminergic therapy' OR 'dopamine modulator' OR 'dopaminergic drug*' OR 'dopaminergic treatment' OR 'dopamine agonist' OR parkinson* OR rls OR 'restles legs' OR fibromyalgia OR 'pituitary adenoma' OR hyperprolactinem* OR aripiprazole OR pramipexole OR ropinirole OR levodopa ) NOT TI ( 'review' OR 'metaanalysis' OR 'meta-analysis' OR 'mouse' OR 'mice' OR 'rat' OR 'rats' OR 'hamster' OR 'rodent*' OR 'animal' OR 'case' )

**Web of Science**
(TS=("dopaminergic medication" OR "dopamine replacement therapy" OR "dopaminergic therapy" OR "dopamine modulator" OR "dopaminergic drug*" OR "dopaminergic treatment" OR "dopamine agonist" OR parkinson* OR RLS OR "restless legs" OR fibromyalgia OR "pituitary adenoma" OR hyperprolactinem* OR aripiprazole OR pramipexole OR ropinirole OR levodopa))

AND

(TS=("impulse control" OR impulsiv* OR compulsiv* OR gambl* OR hypersexual* OR buying OR buyer* OR shopp* OR pund* OR hobbyism))

NOT

(TI=("review" OR "metaanalysis" OR "meta-analysis" OR "mouse" OR "mice" OR "rat" OR "rats" OR "hamster" OR "rodent*" OR "animal" OR "case"))

Refined by: Document Type = Article or Proceeding Paper; Language = English; Years = 2000–2025

## Reviewer 2

**PubMed**
(("dopamine agonists/adverse effects"[MeSH Terms] OR "dopamineagonists/pharmacology"[MeSH Terms] OR "dopamine agonists/therapeutic use"[MeSH Terms] OR "dopamine agents/adverse effects"[MeSH Terms] OR "dopamine agents/pharmacology"[MeSH Terms] OR "pramipexole/adverse effects"[MeSH Terms] OR "pramipexole/pharmacology"[MeSH Terms] OR "pramipexole/therapeutic use"[MeSH Terms] OR "aripiprazole/adverse effects"[MeSH Terms] OR "aripiprazole/pharmacology"[MeSH Terms] OR "aripiprazole/therapeutic use"[MeSH Terms] OR "ropinirole"[MeSH Terms] OR "dopaminergic medication"[Title/Abstract]) AND ("impulse control disorders"[MeSH Terms] OR "disruptive, impulse control, and conduct disorders/chemically induced"[MeSH Terms] OR "impulsive behavior/drug effects"[MeSH Terms] OR "gambling/chemically induced"[MeSH Terms] OR "gambling/therapy"[MeSH Terms] OR "binge-eating disorder"[MeSH Terms] OR "pathological gambling"[MeSH Terms] OR "hoarding disorder"[MeSH Terms] OR "compulsive sexual behavior disorder/therapy"[MeSH Terms] OR "hypersexuality"[All Fields] OR "compulsive shopping"[All Fields] OR "shopping addiction"[All Fields] OR punding[Title/Abstract] OR

hobbyism[Title/Abstract] OR hoard*[Title/Abstract] OR gambl*[Title/Abstract] OR impulsiv*[Title/Abstract] OR compulsiv*[Title/Abstract])) AND ("Parkinson Disease"[MeSH Terms] OR "schizophrenia"[MeSH Terms] OR "bipolar disorder"[MeSH Terms] OR "major depressive disorder"[MeSH Terms] OR "restless legs syndrome"[MeSH Terms] OR "prolactinoma"[MeSH Terms] OR "psychiatric patients"[Title/Abstract])

**Embase**

'dopamine agonist'/exp OR 'dopamine agonist adverse drug reaction' OR 'pramipexole'/exp OR 'aripiprazole'/exp OR 'ropinirole'/exp OR 'dopaminergic agent'/exp OR 'dopaminergic medication':ti,ab

AND

'impulse control disorder'/exp OR 'pathological gambling'/exp OR 'hypersexuality':ti,ab OR 'compulsive buying':ti,ab OR 'compulsive shopping':ti,ab OR 'shopping addiction':ti,ab OR 'punding':ti,ab OR 'hobbyism':ti,ab OR 'gambling'/exp OR 'hoarding disorder'/exp OR 'hoarding':ti,ab OR 'impulsive behavior'/exp OR 'compulsive behavior':ti,ab OR 'impulsivity':ti,ab OR 'compulsivity':ti,ab

AND

'parkinson disease'/exp OR 'schizophrenia'/exp OR 'bipolar disorder'/exp OR 'major depression'/exp OR 'restless legs syndrome'/exp OR 'prolactinoma'/exp OR 'psychiatric patient':ti,ab

**Web Of Science**

TS=("dopamine agonist*" OR "dopaminergic agent*" OR "dopaminergic medication*" OR pramipexole OR ropinirole OR aripiprazole)
AND
TS=("impulse control disorder*" OR "impulsive behavior*" OR "compulsive behavior*" OR "gambling disorder*" OR "pathological gambling" OR hypersexuality OR "compulsive buying" OR "shopping addiction" OR punding OR hobbyism OR hoarding)
AND
TS=("Parkinson's disease" OR schizophrenia OR "bipolar disorder" OR "major depressive disorder" OR "restless legs syndrome" OR prolactinoma OR "psychiatric patient*" OR "neurological disorder*")

**PsycINFO**

("dopamine agonist*" OR "dopaminergic agent*" OR pramipexole OR ropinirole OR aripiprazole OR "dopaminergic medication*") AND DE "Impulse Control Disorders" ORDE "Pathological Gambling" ORDE "Compulsive Behavior" ORDE "Gambling" ORDE "Hoarding" ORTI,AB("impulse control disorder*" OR "impulsive behavior*" OR "compulsive behavior*" OR "gambling disorder*" OR "pathological gambling" OR hypersexuality OR "compulsive shopping" OR "compulsive buying" OR "shopping addiction" OR punding OR hobbyism OR hoarding) AND DE "Parkinson's Disease" ORDE "Schizophrenia" ORDE "Bipolar Disorder" ORDE "Major Depression" ORDE "Restless Legs Syndrome" ORDE "Psychiatric Patients" ORTI,AB("psychiatric patient*" OR "neurological disorder*" OR prolactinoma)
