# Supplementary material for: Clinical aspects of impulsive-compulsive behaviors under dopaminergic treatment—A scoping review
Source: Australas Psychiatry. 2026 Mar 28;34(5):432–6. doi: 10.1177/10398562261439053 (PMC13669450; doi:10.1177/10398562261439053)
Supplement: Supplementary file 1 [file sj-zip-1-apy-10.1177_10398562261439053.zip › Supplementary file S3_Included studies_Overview.pdf]

Abbreviations

**AE**	Adverse event
**ALS**	Amyotrophic lateral sclerosis
**BE**	Binge eating
**BIS-11**	Barratt impulsiveness scale
**BL**	Baseline
**CB**	Compulsive buying
**CSB**	Compulsive sexual behavior
**COMT-I**	COMT inhibitor
**DA**	Dopamine agonist
**DAWS**	Dopamine agonist withdrawal syndrome
**DBS**	Deep brain stimulation
**DDS**	Dopamine dysregulation syndrome
**ER**	Extended-release
**FU**	Follow-up
**GPi**	Globus pallidus interna
**HC**	Healthy control
**ICB**	Impulsive-compulsive behaviour
**ICD**	Impulse control disorder (PG, CSB, CB, BE)
**IGT**	Iowa gambling task
**IR**	Immediate-release
**LCIG**	Levodopa-carbidopa intestinal gel
**LD**	Levodopa
**LEDD**	Levodopa equivalent daily dose
**MAOB-I**	MAO-B inhibitor
**OCD**	Obsessive-compulsive disorder
**PD**	Parkinson's disease
**PG**	Pathological gambling
**PPX**	Pramipexole
**QOL**	Quality of life
**QUIP**	Questionnaire for Impulsive-Compulsive Disorders in Parkinson’s Disease
**RBD**	REM sleep behavior disorder
**RCT**	Randomized controlled trial
**RLS**	Restless legs syndrome
**ROP**	Ropinirole
**STN**	Subthalamic nucleus

#	First author Year	Article title	Country	Study design	Population description	Total N
A1	Abbes 2018 [1]	Subthalamic stimulation and neuropsychiatric symptoms in Parkinson's disease: Results from a long-term follow-up cohort study	France and Switzerland	Cohort study	PD with STN DBS	N=69
A2	Abosch 2011 [2]	Impulsive behavior and associated clinical variables in parkinson's disease	USA	Cross-sectional study	PD	N=128
A3	Ahearn 2012 [3]	An exploration of apathy and impulsivity in Parkinson disease	UK	Cross-sectional study	PD	N=99
A4	Almalki 2024 [4]	Impulse control disorders in patients with dopamine agonist-treated pituitary adenomas: a cross-sectional multicenter study	Saudi Arabia and UAE	Cross-sectional study	Prolactinoma or non-functioning pituitary adenoma under DA treatment	N=200
A5	Alvarado-Bolaños 2015 [5]	Impact of neuropsychiatric symptoms on the quality of life of subjects with Parkinson's disease	Mexico	Cross-sectional study	PD	N=492
A6	Amami 2015 [6]	Impulse control behaviours in patients with Parkinson's disease after subthalamic deep brain stimulation:De novo cases and 3-year follow-up	Italy	Cohort study	PD with STN DBS	N=56
A7	Amstutz 2025 [7]	Impulse Control Disorders and Effort-Based Decision-Making in Parkinson's Disease Patients with Subthalamic Nucleus Deep Brain Stimulation	Switzerland	Cohort study	PD with STN DBS	N=43
A8	Angioletti 2020 [8]	Metacognition deficits and impulsivity in Parkinson's Disease patients with and without gambling behavior: A pilot study	Italy	Cross-sectional study	PD	N=48
A9	Antonini 2016 [9]	Impulse control disorder related behaviours during long-term rotigotine treatment: a post hoc analysis	International	Post hoc analysis of 6 studies	PD with rotigotine treatment	N=786
A10	Antonini 2017 [10]	ICARUS study: Prevalence and clinical features of impulse control disorders in Parkinson's disease	Italy	Cohort study	PD	N=1069
A11	Aoki 2019 [11]	Deterioration of postural deformity in Parkinson's disease patients with punding and hobbyism	Japan	Cross-sectional study	PD	N=80
A12	Ardouin 2006 [12]	Pathological gambling in Parkinson's disease improves on chronic subthalamic nucleus stimulation	France and Canada	Cohort study	PD with STN DBS	N=598
A13	Ataoglu 2024 [13]	Impulse control disorders related cognitive and behavioral features in Parkinson's disease	Turkey	Cross-sectional study	PD and HCs	N=139 (72+67)
A14	Aumann 2020 [14]	Self-reported rates of impulsivity in Parkinson's Disease	USA	Cross-sectional study	PD and HCs	N=204 (111+93)
A15	Auyeung 2011 [15]	Impulse control disorders in Chinese Parkinson's disease patients: The effect of ergot derived dopamine agonist	China	Cross-sectional study	PD	N=213
A16	Avanzi 2006 [16]	Prevalence of pathological gambling in patients with Parkinson's disease	Italy	Cross-sectional study	PD and HCs	N=490 (98+392)

A17	Ávila 2011 [17]	Impulsive and compulsive behaviors in Parkinson's disease: A one-year follow-up study	Spain	Cohort study	PD with ICBs	N=25
A18	Azmin 2016 [18]	Impulse control behaviours in a Malaysian Parkinson's disease population	Malaysia	Cross-sectional study	PD	N=80
A19	Baig 2019 [19]	Impulse control disorders in Parkinson disease and RBD: A longitudinal study of severity	UK	Combined cohort and qualitative study	PD, RBD, HCs	N=489 (92+102+295)
A20	Bancos 2014 [20]	Impulse control disorders in patients with dopamine agonist-treated prolactinomas and nonfunctioning pituitary adenomas: A case-control study	USA	Case-control study	Prolactinoma with DA treatment and nonfunctioning pituitary adenoma without DA treatment	N=147 (77+70)
A21	Barake 2014 [21]	Investigation of impulsivity in patients on dopamine agonist therapy for hyperprolactinemia: A pilot study	USA	Cross-sectional study	Hyperprolactinemia treated, untreated, HCs	N=30 (10+10+10)
A22	Barbosa 2017 [22]	A Retrospective Evaluation of the Frequency of Impulsive Compulsive Behaviors in Parkinson's Disease Patients Treated with Continuous Waking Day Apomorphine Pumps	UK	Patient records review	PD with apomorphine infusion	N=28
A23	Barbosa [23] 2018	The Outcome of Dopamine Dysregulation Syndrome in Parkinson's Disease: A Retrospective Postmortem Study	UK	Patient records review	PD with and without DDS	N=193 (17+176)
A24	Barbosa 2018 [24]	Compulsive sexual behaviour in Parkinson's disease is associated with higher doses of levodopa	UK	Patient records review	PD with ICB	N=111
A25	Barbosa 2019 [25]	Lower nucleus accumbens α-synuclein load and D3 receptor levels in Parkinson's disease with impulsive compulsive behaviours	UK	Case-control study	PD with ICB and PD controls	N=60 (31+29)
A26	Barbosa 2022 [26]	The prevalence of impulsive compulsive behaviors in patients treated with apomorphine infusion: A retrospective analysis	UK	Patient records review	PD with apomorphine infusion	N=193
A27	Barbosa 2022 [27]	Neuropsychiatric Features of Punding and Hobbyism in Parkinson's Disease	UK	Cross-sectional study	PD with punding, hobbyism or neither	N=72 (21+26+25)
A28	Barone 2019 [28]	Risk factors for impulse control disorders and related behaviors in Parkinson's disease: secondary analyses of the ICARUS study	Italy	Cohort study	PD	N=712
A29	Bastiaens 2013 [29]	Prospective cohort study of impulse control disorders in Parkinson's disease	USA	Cohort study	PD without ICDs at baseline	N=164 (46 analysed for incident ICDs)
A30	Baumann-Vogel 2015 [30]	Impulse control disorders in Parkinson's disease: Don't set your mind at rest by self-assessments	Switzerland	Cross-sectional study	PD	N=150
A31	Bayard 2013 [31]	Decision-making, reward-seeking behaviors and dopamine agonist therapy in restless legs syndrome	France	Case-control study	RLS treated, untreated, HCs	N=149 (50+39+60)
A32	Bayard 2014 [32]	Impulse control disorder and rapid eye movement sleep behavior disorder in Parkinson's disease	France	Cross-sectional study	PD	N=98

A33	Beccuti 2021 [33]	Increased prevalence of impulse control disorder symptoms in endocrine diseases treated with dopamine agonists: a cross-sectional study	Italy	Cross-sectional study	Pituitary disorder with or without DA use	N=190 (132+58)
A34	Bentivoglio 2013 [34]	Neuropsychological features of patients with Parkinson's disease and impulse control disorders	Italy	Case-control study	PD with and without ICD	N=34 (17+17)
A35	Benussi 2017 [35]	Modulating risky decision-making in Parkinson's disease by transcranial direct current stimulation	Italy	RCT	PD	N=60 (20 anodal, 20 cathodal, 20 sham)
A36	Bermejo 2010 [36]	Zonisamide in managing impulse control disorders in Parkinson's disease	Spain	Intervention study other than RCT	PD with ICDs	N=15
A37	Bharmal 2010 [37]	Outcomes of patients with Parkinson disease and pathological gambling	Canada	Cohort study	PD	N=146
A38	Binck 2020 [38]	Contributing Factors and Evolution of Impulse Control Disorder in the Luxembourg Parkinson Cohort	Luxembourg	Cohort study	PD with and without DA treatment, matched HCs	BL: N=899 (253+217+429) FU: N=156 (102+54+0)
A39	Biundo 2017 [39]	Impulse control disorders in advanced Parkinson's disease with dyskinesia: The ALTHEA study	Italy	Cross-sectional study	PD	N=251
A40	Bostwick 2009 [40]	Frequency of new-onset pathologic compulsive gambling or hypersexuality after drug treatment of idiopathic Parkinson disease	USA	Patient records review	PD	N=267
A41	Brigas 2024 [41]	Living with Parkinson's disease in Portugal: Findings from PRISM study	Portugal	Cross-sectional study	PD	N=80
A42	Bril 2017 [42]	A multifactorial study on nutritional status, binge eating and physical activity as main factors directly influencing body weight in Parkinson's disease	Argentina	Cross-sectional study	PD	N=114
A43	Cabrini 2009 [43]	Preliminary evaluation of the DDS-PC inventory: A new tool to assess impulsive - Compulsive behaviours associated to dopamine replacement therapy in Parkinson's disease	Italy	Cross-sectional study	PD with and without DDS	N=38 (10+28)
A44	Callesen 2014 [44]	Impulsive and compulsive behaviors among Danish patients with Parkinson's disease: Prevalence, depression, and personality	Denmark	Cross-sectional study	PD	N=490
A45	Callesen 2017 [45]	Phenomenology and gender characteristics of hobbyism and punding in Parkinson's disease: A self-report study	Denmark	Cross-sectional study	PD	N=490
A46	Canesi 2020 [46]	Hypomania, depression, euthymia: New evidence in Parkinson's disease	Italy	Cross-sectional study	PD and HCs	N=143 (93+50)
A47	Cao 2020 [47]	Cross-sectional and longitudinal associations between probable rapid eye movement sleep behavior disorder and impulse control disorders in Parkinson's disease	China	Cohort study	PD	N=320
A48	Carbunaru 2018 [48]	Impulse control disorders in Parkinson's: Sleep disorders and nondopaminergic associations	Mexico	Cross-sectional study	PD	N=53

A49	Castrioto 2015 [49]	Iowa gambling task impairment in Parkinson's disease can be normalised by reduction of dopaminergic medication after subthalamic stimulation	France	Cohort study	PD with STN DBS and HCs	N=44 (20+24)
A50	Catalán 2013 [50]	Levodopa infusion improves impulsivity and dopamine dysregulation syndrome in Parkinson's disease	Spain	Cohort study	PD with ICB with LCIG infusion	N=8
A51	Catalan 2018 [51]	Improvement of impulse control disorders associated with levodopa–carbidopa intestinal gel treatment in advanced Parkinson's disease	Spain	Cohort study	PD	N=62
A52	Celik 2018 [52]	Impulse control disorders in patients with prolactinoma receiving dopamine agonist therapy: a prospective study with 1 year follow-up	Turkey	Cohort study	Prolactinoma group (DA-treated), non-functioning pituitary adenoma (untreated), HCs	N=88 (25+31+32)
A53	Cera 2014 [53]	Amantadine and cognitive flexibility: Decision making in Parkinson's patients with severe pathological gambling and other impulse control disorders	Italy	Cohort study	PD under amantadine treatment with PG, with other ICD, without ICD	N=33 (10+9+14)
A54	Chaudhuri 2015 [54]	A Pilot Prospective, Multicenter Observational Study of Dopamine Agonist Withdrawal Syndrome in Parkinson's Disease	UK, Germany, Denmark, Sweden, Italy, Spain	Cohort study	PD with DA tapering/discontinuation	N=51
A55	Chen 2023 [55]	Association between metabolic syndrome components and impulse control disorders in Parkinson's disease	China	Cross-sectional study	PD with and without ICDs	N=105 (39+66)
A56	Chen 2024 [56]	APOE contributes to longitudinal impulse control disorders progression in Parkinson's disease	International (majority of sites in the USA plus Europe, Israel, Australia)	Cohort study	De novo or early PD, drug-naïve at enrollment	N=287
A57	Chiang 2012 [57]	Are there ethnic differences in impulsive/compulsive behaviors in Parkinson's disease?	Taiwan	Cross-sectional study	PD	N=268
A58	Choi 2020 [58]	Neuropsychiatric Traits Associated with Refractory Impulse Control Disorder in Parkinson's Disease	South Korea	Case-control study	PD under DA substitution with LD	N=38
A59	Cilia 2014 [59]	Dopamine dysregulation syndrome in Parkinson's disease: From clinical and neuropsychological characterisation to management and long-term outcome	Italy	Case-control study	PD with and without DDS (matched)	N=105 (35+70)
A60	Contin 2023 [60]	Screening for impulse control disorders in Parkinson's disease and dopamine agonist use: a study of pharmacokinetic and psychological risk factors	Italy	Cross-sectional study	PD under DA treatment	N=105
A61	Cooper 2009 [61]	Prevalence of hypersexual behavior in Parkinson's disease patients: Not restricted to males and dopamine agonist use	USA	Cross-sectional study	PD	N=141

A62	Corbeil 2021 [62]	Problem Gambling Associated with Aripiprazole: A Nested Case-Control Study in a First-Episode Psychosis Program	Canada	Case-control study	Treated in single first-episode psychosis program	N=219
A63	Corvol 2018 [63]	Longitudinal analysis of impulse control disorders in Parkinson disease	France	Cohort study	Early PD	N=411
A64	Crockford 2008 [64]	Prevalence of problem and pathological gambling in Parkinson's disease	Canada	Cross-sectional study	PD	N=140
A65	Cuciureanu 2019 [65]	Neuropsychiatric changes in parkinson's disease patients: A prospective observational two years study	Romania	Cross-sectional study	PD	N=112
A66	Cunnington 2012 [66]	Identification of possible risk factors for the development of dopamine agonist withdrawal syndrome in Parkinson's disease	UK	Patient records review	PD with DA AEs	N=80
A67	Dalrymple 2024 [67]	Psychiatric predictors of quality of life in Parkinson's disease: A three-year longitudinal study	USA	Cohort study	PD	N=105
A68	Dang 2011 [68]	The emergence of devastating impulse control disorders during dopamine agonist therapy of the restless legs syndrome	Australia	Cross-sectional study	RLS with DA treatment and ICDs	N=12
A69	Dawood 2020 [69]	Safety and effectiveness of long-acting injectable Aripiprazole in daily practice: A naturalistic retrospective study	UK	Patient records review	Under aripiprazole depot treatment	N=57
A70	De Chazeron 2011 [70]	Hypersexuality and pathological gambling in Parkinson's disease: A cross-sectional case-control study	France	Case-control study	PD and HCs	N=254 (139+115)
A71	De Chazeron 2019 [71]	Compulsive eating behaviors in Parkinson's disease	France	Cross-sectional study	PD	N=51
A72	Delaney 2012 [72]	Perceptions of cause and control of impulse control behaviours in people with Parkinson's disease	UK	Qualitative study	PD with current or recent ICB	N=10
A73	De la Riva 2014 [73]	Course of psychiatric symptoms and global cognition in early Parkinson disease	USA and Europe	Cohort study	New onset PD, HCs	N=406 (261+145)
A74	Delpont 2017 [74]	Psychostimulant effect of dopaminergic treatment and addictions in Parkinson's disease	France	Cohort study	PD with STN DBS	N=102
A75	De Sousa 2020 [75]	Impulse Control Disorders in Dopamine Agonist-Treated Hyperprolactinemia: Prevalence and Risk Factors	Australia	Cross-sectional study	DA-treated hyperprolactinaemia and HCs	N=212 (113+99)
A76	De Wit 2022 [76]	Impulse control disorders associated with dopaminergic drugs: A disproportionality analysis using vigibase	International	Pharmacovigilance study	Reports of dopaminergic drug AEs (ICD cases and non-cases)	N=282,572 (852+281,720)
A77	Diago 2017 [77]	Association between REM sleep behaviour disorder and impulse control disorder in Parkinson's disease; patients with Parkinson's disease	Spain	Cross-sectional study	PD under DA treatment	N=73
A78	Di Rosa 2022 [78]	Anxiety predicts impulsive-compulsive behaviours in Parkinson's disease: Clinical relevance and theoretical implications	International	Cohort study	De novo PD (drug-naive at enrollment), Parkinsonism but Scan Without Evidence of Dopaminergic Deficit (stayed drug-naïve), HCs	N=652 (417+50+185)
A79	Dogansen 2019 [79]	Dopamine Agonist-Induced Impulse Control Disorders in Patients with Prolactinoma: A Cross-Sectional Multicenter Study	Turkey	Cross-sectional study	Prolactinoma patients on DA ≥3 months	N=398

A80	Drew 2020 [80]	Dopamine and reward hypersensitivity in Parkinson's disease with impulse control disorder	UK	Cross-sectional and cohort study	PD with and without ICDs, HCs	N=79 (23+26+31)
A81	Driver-Dunckley 2007 [81]	Gambling and increased sexual desire with dopaminergic medications in restless legs syndrome	USA	Cross-sectional study	RLS	N=70
A82	Du 2023 [82]	The mediating effects of depression, anxiety, and rapid eye movement sleep behavior disorder on the association between dopaminergic replacement therapy and impulse control disorders in Parkinson’s disease	International	Cohort study	PD	N=268
A83	Dubaz 2019 [83]	Changes in Prescribing Practices of Dopaminergic Medications in Individuals with Parkinson's Disease by Expert Care Centers from 2010 to 2017: The Parkinson's Foundation Quality Improvement Initiative	International	Register study	PD in 2010 and 2017	N=2717, N=2900
A84	El Otmani 2019 [84]	Impulse control disorders in Parkinson disease: A cross-sectional study in Morocco	Morocco	Cross-sectional study	PD	N=125
A85	Erdal 2021 [85]	Obsessive–compulsive disorder in restless legs syndrome	Turkey	Cross-sectional study	RLS and HCs	N=213 (113+100)
A86	Erga 2017 [86]	Impulsive and Compulsive Behaviors in Parkinson's Disease: The Norwegian ParkWest Study	Norway	Cross-sectional study	PD and HCs (matched)	N=184 (25+159)
A87	Erga 2020 [87]	Evolution of impulsive–compulsive behaviors and cognition in Parkinson’s disease	Norway	Cohort study	PD and HCs (matched)	N=280 (124+156)
A88	Erga 2020 [88]	Impulsive and compulsive behaviors in Parkinson's disease: Impact on quality of and satisfaction with life, and caregiver burden	Norway	Cross-sectional study	PD without dementia and caregivers	N=202 (101+101)
A89	Etminan 2017 [89]	Risk of Gambling Disorder and Impulse Control Disorder with Aripiprazole, Pramipexole, and Ropinirole: A Pharmacoepidemiologic Study	USA	Pharmacovigilance study	PG and ICD AE reports and controls each	N=3905 (355+3550), N=47751 (4341+43410)
A90	Eusebio 2013 [90]	Subthalamic nucleus stimulation and compulsive use of dopaminergic medication in Parkinson's disease	France	Cohort study	PD with STN DBS	N=110
A91	Evans 2004 [91]	Punding in Parkinson's disease: Its relation to the dopamine dysregulation syndrome	UK	Cross-sectional study	PD with and without punding	N=123 (17+106)
A92	Evans 2005 [92]	Factors influencing susceptibility to compulsive dopaminergic drug use in Parkinson disease	UK	Cross-sectional study	PD with DDS, without DDS, HCs	N=150 (25+25+100)
A93	Evans 2010 [93]	Compulsive use of dopaminergic drug therapy in Parkinson's disease: Reward and anti-reward	UK	Case-control study	PD wih and without DDS	N=40 (20+20)
A94	Evans 2014 [94]	A conditioned response as a measure of impulsive-compulsive behaviours in Parkinson's disease	Australia	Cross-sectional study	PD	N=25
A95	Fabbri 2024 [95]	Amantadine use in the French prospective NS-Park cohort	France	Cohort study	PD	N=12,542

A96	Fan 2009 [96]	Impulse control disorders in Parkinson's disease in a Chinese population	China	Cross-sectional study	PD (drug-naive) and caregivers as controls	N=444 (312+132)
A97	Fantini 2015 [97]	Increased risk of impulse control symptoms in Parkinson's disease with REM sleep behaviour disorder	France and Italy	Cross-sectional study	PD with and without RBD	N=216 (106+110)
A98	Fantini 2018 [98]	Sleep and REM sleep behaviour disorder in Parkinson's disease with impulse control disorder	France and Italy	Case-control study	PD with and without ICDs	N=80 (40+40)
A99	Fantini 2020 [99]	Is Rapid Eye Movement Sleep Behavior Disorder a Risk Factor for Impulse Control Disorder in Parkinson Disease?	International	Cohort study	Early PD with probable RBD	N=401
A100	Faouzi 2022 [100]	Machine Learning-Based Prediction of Impulse Control Disorders in Parkinson's Disease From Clinical and Genetic Data	North America and Europe	Cohort study	De novo or early PD	N=768 (380+388)
A101	Farnikova 2012 [101]	Comparison of personality characteristics in parkinson disease patients with and without impulse control disorders and in healthy volunteers	Czech Republic	Cross-sectional study	PD and HCs	N=102 (46+56)
A102	Fasano 2011 [102]	Management of punding in Parkinson's disease: An open-label prospective study	Italy	Intervention study other than RCT	PD with punding	N=10
A103	Fusaroli 2022 [103]	Impulse Control Disorders by Dopamine Partial Agonists: A Pharmacovigilance-Pharmacodynamic Assessment through the FDA Adverse Event Reporting System	International	Pharmacovigilance study	Under third-generation antipsychotic treatment with ICBs and control groups	N=8328 (2708+5620)
A104	Fusaroli 2023 [104]	Exploring the underlying mechanisms of drug-induced impulse control disorders: a pharmacovigilance-pharmacodynamic study	International	Pharmacovigilance study	ICD reports	N=19,887
A105	Fusaroli 2024 [105]	Unveiling the Burden of Drug-Induced Impulsivity: A Network Analysis of the FDA Adverse Event Reporting System	International	Pharmacovigilance study	Reports of impulsivity as AE of PPX or aripiprazole	N=27,601, N=80,238
A106	Galbiati 2015 [106]	Neurocognitive function in patients with idiopathic Restless Legs Syndrome before and after treatment with dopamine-agonist	Italy	Case-control study	Untreated RLS and HCs	N=30 (20+15)
A107	García-Rubio 2021 [107]	Analysis of Impulse Control Disorders (ICDs) and Factors Associated with Their Development in a Parkinson's Disease Population	Mexico	Cross-sectional study	PD with and without ICD	N=181 (80+101)
A108	Garcia-Ruiz 2014 [108]	Impulse control disorder in patients with Parkinson's disease under dopamine agonist therapy: A multicentre study	Spain	Cross-sectional study	PD under DA treatment for at least 6 months	N=233
A109	Garrett 2019 [109]	Epidemiology of impulse control disorders and association with dopamine agonist exposure, active component, U.S. Armed Forces, 2014-2018	US	Pharmacovigilance study	Active-duty U.S. military personnel with and without ICDs	N=31,865 (6373+25,492)
A110	Gee 2015 [110]	The Influence of Bilateral Subthalamic Nucleus Deep Brain Stimulation on Impulsivity and Prepulse Inhibition in Parkinson's Disease Patients	USA	Cohort study	PD with STN DBS	N=16
A111	Gendreau 2014 [111]	Detecting associations between behavioral addictions and dopamine agonists in the Food & Drug Administration's Adverse Event database	International	Pharmacovigilance study	Reports with ICDs as AE 2004 and 2007	N=375, N=607
A112	Gescheidt 2016 [112]	Impulse control disorders in patients with young-onset Parkinson's disease: A cross-sectional study seeking associated factors	Czech Republic	Case-control study	Young-onset PD and HCs	N=87 (49+38)

A113	Giladi 2007 [113]	New onset heightened interest or drive for gambling, shopping, eating or sexual activity in patients with Parkinson's disease: The role of dopamine agonist treatment and age at motor symptoms onset	Israel	Case-control study	PD and HCs	N=383 (193+190)
A114	Giladi 2013 [114]	The safety and tolerability of rotigotine transdermal system over a 6-year period in patients with early-stage Parkinson's disease	Australia, New Zealand, South Africa, Europe	Intervention study other than RCT	PD under rotigotine treatment	N=380
A115	Goerlich-Dobre 2014 [115]	Alexithymia-an independent risk factor for impulsive-compulsive disorders in Parkinson's disease	Germany	Cross-sectional study	PD	N=91
A116	Gómez 2017 [116]	A multi center comparative study of impulse control disorder in Latin American patients with Parkinson disease	Latin America	Cross-sectional study	PD	N=255
A117	Grall-Bronnec 2022 [117]	Parkinson's disease and iatrogenic impulsive- compulsive behaviors: A case/non-case study to build a complete model of individual vulnerability	France	Cohort study	PD with and without ICB increase	N=163 (57+106)
A118	Grall-Bronnec 2024 [118]	Parkinson's Disease, Impulsive-Compulsive Behaviors, and Health-Related Quality of Life	France	Cohort study	PD treated ≥ 6 months with dopaminergics	N=169
A119	Grosset 2006 [119]	Problematic gambling on dopamine agonists: Not such a rarity	UK	Cohort study	PD	N=388
A120	Gunduz 2024 [120]	Impulse control disorders in Parkinson's disease: a retrospective analysis of 1824 patients in a 12-year period	Turkey	Patient records review	PD	N=1824
A121	Gungabissoon 2020 [121]	Comparison of long-term use of prolonged-release ropinirole and immediate-release dopamine agonists in an observational study in patients with Parkinson's disease	UK	Cohort study	PD under ROP ER or IR DA	N=682 (341+341)
A122	Hack 2014 [122]	Impulsive and compulsive behaviors in Parkinson Study Group (PSG) centers performing deep brain stimulation surgery	USA	Cross-sectional study	PD treatment centers	N=48
A123	Hall 2023 [123]	Performance on the balloon analogue risk task and anticipatory response inhibition task is associated with severity of impulse control behaviours in people with Parkinson's disease	UK	Cross-sectional study	PD with and without DA treatment	N=83 (58+25)
A124	Hassan 2011 [124]	Dopamine agonist-triggered pathological behaviors: Surveillance in the PD clinic reveals high frequencies	USA	Patient records review	PD under DA treatment	N=321
A125	Hauser 2014 [125]	Long-term safety and sustained efficacy of extended-release pramipexole in early and advanced Parkinson's disease	Asia and Europe	Intervention study other than RCT	Early/advanced PD with PPX IR/ER treatment or placebo	N=902 (511+391)
A126	Heim 2016 [126]	Augmentation and impulsive behaviors in restless legs syndrome: Coexistence or association?	Austria	Case-control study	RLS	N=58
A127	Heim 2018 [127]	Reflection impulsivity perceptual decision-making in patients with restless legs syndrome	Austria	Case-control study	RLS drug-naive, on dopaminergic treatment, HCs	N=55 (20+15+20)
A128	Heim 2021 [128]	Birds of a feather flock together: Disadvantageous decision making in augmented restless legs syndrome patients with and without impulse control disorders	Austria	Cohort study	RLS with augmentation with and without ICDs, HCs	N=61 (19+21+21)

A129	Herath 2024 [129]	Impulse control disorders and other nonmotor symptoms in Sri Lankan patients with Parkinson's disease	Sri Lanka	Cohort study	PD	N=192
A130	Hernandez-Con 2023 [130]	Course of Impulse Control Disorder Symptoms in Parkinson's Disease: Deep Brain Stimulation Versus Medications	USA	Case-control study within cohort	PD with DBS and PD controls	N=54 (26+28)
A131	Hidding 2017 [131]	Impact of Combined Subthalamic Nucleus and Substantia Nigra Stimulation on Neuropsychiatric Symptoms in Parkinson's Disease Patients	Germany	RCT	PD with STN DBS	N=9
A132	Hinkle 2021 [132]	Attentional dysfunction and the punding spectrum in Parkinson's disease	International	Cohort study	PD (drug-naïve at enrollment)	N=484
A133	Hinojosa-Amaya 2020 [133]	Depression and Impulsivity Self-Assessment Tools to Identify Dopamine Agonist Side Effects in Patients With Pituitary Adenomas	USA	Cross-sectional study	Pituitary adenoma under DA treatment or drug naive	N=94 (67+27)
A134	Hollingworth 2015 [134]	Dopamine Agonists: Time Pattern of Adverse Effects Reporting in Australia	Australia	Pharmacovigilance study	ICD AEs under DA treatment	N=220
A135	Holman 2009 [135]	Impulse control disorder behaviors associated with pramipexole used to treat fibromyalgia	USA	Cohort study	Fibromyalgia under DA treatment with PG/CB	N=21
A136	Hu 2023 [136]	Different impulse control disorder evolution patterns and white matter microstructural damage in the progression of Parkinson's disease	USA, Europe, Israel, Australia	Cohort study	De novo PD	N=321
A137	Hurt 2014 [137]	Motor phenotypes, medication and mood: Further associations with impulsive behaviours in Parkinson's disease	UK	Cross-sectional study	PD	N=500
A138	Hälbig 2009 [138]	Subthalamic deep brain stimulation and impulse control in Parkinson's disease	USA	Cross-sectional study	PD with and without STN DBS	N=53 (16+37)
A139	Ihle 2020 [139]	Parkinson's disease polygenic risk score is not associated with impulse control disorders: A longitudinal study	France	Cohort study	PD	N=403
A140	Imamura 2008 [140]	Medications used to treat Parkinson's disease and the risk of gambling	USA	Case-control study	PD with and without PG	N=48 (11+37)
A141	Inoue 2010 [141]	Efficacy, safety and dose-response of pramipexole in Japanese patients with primary restless legs syndrome: Randomized trial	Japan	Intervention study other than RCT	RLS under PPX treatment	N=141
A142	Irmen 2019 [142]	Sensorimotor subthalamic stimulation restores risk-reward trade-off in Parkinson's disease	Germany	Case-control study	PD patients treated with STN DBS and HCs	N=34 (17+17)
A143	Isaias 2008 [143]	The relationship between impulsivity and impulse control disorders in Parkinson's disease	Italy	Cross-sectional study	PD and HCs	N=150 (50+100)
A144	Izzo 2020 [144]	Impulse control disorders in Parkinson's disease versus in healthy controls: A different predictive model	Italy	Cross-sectional study	PD and HCs	N=93 (51+42)
A145	Jaakkola 2014 [145]	Impulse control disorders are associated with multiple psychiatric symptoms in Parkinson's disease	Finland	Cross-sectional study	PD	N=279
A146	Jakobsen 2016 [146]	Neurological, metabolic, and psychiatric adverse events in children and adolescents treated with aripiprazole	Denmark	Pharmacovigilance study	Under aripiprazole treatment for psychotic and other disorders (<18 yrs)	N=19 (5+14)

A147	Janssen 2014 [147]	Subthalamic nucleus high-frequency stimulation for advanced Parkinson's disease: Motor and neuropsychological outcome after 10 years	Netherlands	Cohort study	PD with STN DBS	N=26
A148	Jeon 2020 [148]	What drugs modify the risk of iatrogenic impulse-control disorders in Parkinson's disease? A preliminary pharmacoepidemiologic study	USA	Cohort study	PD with PPX/ROP as first treatment	N=935
A149	Jesús 2020 [149]	Non-motor symptom burden in patients with Parkinson's disease with impulse control disorders and compulsive behaviours: results from the COPPADIS cohort	Spain	Cohort study	PD and HCs	N=792 (613+179)
A150	Jesús 2021 [150]	Integrating genetic and clinical data to predict impulse control disorders in Parkinson's disease	Spain	Cross-sectional study	PD	N=353
A151	Jeyadevan 2023 [151]	Quality of life implications for elevated trait impulsivity in people with Parkinson's disease	Australia	Cross-sectional study	PD without previous ICD	N=322
A152	Jiménez-Murcia 2012 [152]	Cognitive-Behavioral Therapy for Pathological Gambling in Parkinson's Disease: A Pilot Controlled Study	Spain	Intervention study other than RCT	PG with or without PD	N=60 (15+45)
A153	Johnson 2023 [153]	Predictors of Burden in Carers of Patients with Impulse Control Behaviors in Parkinson's Disease	UK	Cross-sectional study	PD with ICBs	N=45
A154	Joutsa 2012 [154]	Effects of dopamine agonist dose and gender on the prognosis of impulse control disorders in Parkinson's disease	Finland	Cohort study	PD	N=290
A155	Joutsa 2012 [155]	Impulse control disorders and depression in Finnish patients with Parkinson's disease	Finland	Cross-sectional study	PD	N=575
A156	Kapsomenakis 2023 [156]	Frequency of Impulsive-Compulsive Behavior and Associated Psychological Factors in Parkinson's Disease: Lack of Control or Too Much of It?	Greece	Part A: Patient records review<br>Part B: Cross-sectional study	Part A: PD on DA treatment<br>Part B: PD patients	Part A, N=892<br>Part B, N=30
A157	Ke 2022 [157]	The side effects of dopamine receptor agonist drugs in Chinese prolactinoma patients: a cross sectional study	China	Cross-sectional study	Prolactinoma with DA treatment, prolactinoma/pituitary adenoma without DA, HCs	N=96 (51+12+33)
A158	Kehagia 2014 [158]	Targeting impulsivity in Parkinson's disease using atomoxetine	UK	RCT	PD	N=25
A159	Kenangil 2010 [159]	Compulsive behaviors in patients with Parkinson's disease	Turkey	Case-control study	PD with and without ICD	N=98 (33+65)
A160	Kim 2013 [160]	Clinical characteristics of impulse control and repetitive behavior disorders in Parkinson's disease	South Korea	Cross-sectional study	PD	N=297
A161	Kim 2013 [161]	Impulse control and related behaviors after bilateral subthalamic stimulation in patients with Parkinson's disease	South Korea	Cohort study	PD with STN DBS	N=89
A162	Kim 2014 [162]	REM sleep behavior disorder: Association with motor complications and impulse control disorders in Parkinson's disease	South Korea	Cross-sectional study	PD	N=944
A163	Kim 2018 [163]	A 7-year observation of the effect of subthalamic deep brain stimulation on impulse control disorder in patients with Parkinson's disease	South Korea	Cohort study	PD with STN DBS	N=61

A164	Kobylecki 2020 [164]	The association between pain and impulse control behaviours in Parkinson's disease	UK	Cross-sectional study	PD	N=1909
A165	Koh 2022 [165]	A single-arm open-label pilot study of brief mindfulness meditation to control impulsivity in Parkinson's disease	Japan	Intervention study other than RCT	PD	N=20
A166	Kon 2018 [166]	The factors associated with impulse control behaviors in Parkinson's disease: A 2-year longitudinal retrospective cohort study	Japan	Cohort study	PD	N=141
A167	Kovács 2019 [167]	Screening for problematic Internet use may help identify impulse control disorders in Parkinson's disease	Hungary	Cross-sectional study	PD without previous ICDs	N=106
A168	Kraemmer 2016 [168]	Clinical-genetic model predicts incident impulse control disorders in Parkinson's disease	International	Cohort study	De novo PD without ICDs	N=276
A169	Krause 2022 [169]	Deep brain stimulation in Early Onset Parkinson's disease	Germany	Cohort study	Early-onset PD	N=46
A170	Krause 2022 [170]	Motor and non-motor improvements following short-term multidisciplinary day-clinic care in Parkinson´s disease	Germany	Intervention study other than RCT	PD	N=143
A171	Kuhlman 2019 [171]	Predictors of health-related quality of life in Parkinson's disease	USA	Cross-sectional study	PD	N=102
A172	Kuzu 2016 [172]	Switch from immediate-release pramipexole to extended-release pramipexole: The safety and efficacy characteristics of sixty-eight patients	Turkey	Patient records review	PD with switch from PPX IR to ER	N=68
A173	Lamberti 2016 [173]	Profile of Neuropsychiatric Symptoms in Parkinson's Disease: Surgical Candidates Compared to Controls	France	Cross-sectional study	PD DBS candidates and PD controls	N=494 (234+260)
A174	Laurencin 2023 [174]	Efficacy and safety of clonidine for the treatment of impulse control disorder in Parkinson's disease: a multicenter, parallel, randomised, double-blind, Phase 2b Clinical trial	France	RCT	PD with ICDs treated with clonidine or placebo	N=39 (19+20)
A175	Lawrence 2007 [175]	Predictors of punding in Parkinson's disease: Results from a questionnaire survey	UK	Cross-sectional study	PD and HCs	N=244 (141+103)
A176	Lee 2010 [176]	Association between the dose of dopaminergic medication and the behavioral disturbances in Parkinson disease	South Korea	Cross-sectional study	PD	N=1167
A177	Lee 2019 [177]	Behavioural and trait changes in parkinsonian patients with impulse control disorder after switching from dopamine agonist to levodopa therapy: Results of REIN-PD trial	South Korea	Intervention study other than RCT	PD with ICD, without ICD and medicated, without ICD and drug-naive	N=150 (50+60+40)
A178	Leplow 2023 [178]	Characteristics of behavioural addiction in Parkinson's disease patients with self-reported impulse control disorder and controls matched for levodopa equivalent dose: A matched case–control study	Germany	Case-control study	PD with and without ICDs	N=64
A179	Leroi 2011 [179]	Behavioural disorders, disability and quality of life in Parkinson's disease	UK	Cross-sectional study	PD	N=99
A180	Leroi 2012 [180]	Carer burden in apathy and impulse control disorders in Parkinson's disease	UK	Cross-sectional study	PD with ICD, apathy, PD controls (and caregivers)	N=71 (21+22+28)

A181	Leroi 2012 [181]	Apathy and impulse control disorders in Parkinson's disease: A direct comparison	UK	Case-control study	PD with ICD, apathy, PD controls	N=99 (35+26+38)
A182	Leroi 2013 [182]	Dopaminergic influences on executive function and impulsive behaviour in impulse control disorders in Parkinson's disease	UK	Cross-sectional study	PD with and without ICD, HCs	N=110 (35+55+20)
A183	Lertxundi 2018 [183]	Aripiprazole and impulse control disorders: Higher risk with the intramuscular depot formulation?	Europe	Pharmacovigilance study	ICD reports under aripiprazole treatment	N=160
A184	Lhommée 2012 [184]	Subthalamic stimulation in Parkinson's disease: Restoring the balance of motivated behaviours	France	Cohort study	PD with STN DBS	N=63
A185	Liang 2023 [185]	Predictors of dopamine dysregulation syndrome in patients with early Parkinson's disease	Canada	Cohort study	Early PD	N=283
A186	Lim 2011 [186]	Impulsive-compulsive behaviors are common in Asian Parkinson's disease patients: Assessment using the QUIP	Malaysia	Cross-sectional study	PD	N=200
A187	Limotai 2012 [187]	Addiction-like manifestations and Parkinson's disease: a large single center 9-year experience	USA	Patient records review	PD	N=1040
A188	Lipford 2012 [188]	Long-term use of pramipexole in the management of restless legs syndrome	USA	Cohort study	RLS under PPX treatment	N=50
A189	Liu 2021 [189]	Neuropsychiatric Symptoms in Parkinson's Disease After Subthalamic Nucleus Deep Brain Stimulation	Japan	Cohort study	PD with STN DBS	N=54
A190	Lopiano 2019 [190]	Motor and non-motor outcomes in patients with advanced Parkinson's disease treated with levodopa/carbidopa intestinal gel: final results of the GREENFIELD observational study	Italy	Cohort study	PD under LCIG treatment	N=145
A191	Lubomski 2021 [191]	Health-Related Quality of Life for Parkinson's Disease Patients and Their Caregivers	Australia	Cross-sectional study	PD and caregivers	N=184 (103+81)
A192	Lulé 2012 [192]	Deep brain stimulation and behavioural changes: Is comedication the most important factor?	Germany	Cross-sectional study	PD with STN DBS or under DA treatment	N=30 (15+15)
A193	Lyons 2010 [193]	Orally disintegrating selegiline in Parkinson patients with dopamine agonist-related adverse effects	USA	Intervention study other than RCT	PD with DA-related AEs	N=60
A194	Maggi 2024 [194]	Validation of the Italian version of the Questionnaire for Impulsive-Compulsive Disorders in Parkinson's Disease-Rating Scale (QUIP-RS) in an Italian Parkinson's disease cohort	Italy	Cross-sectional study	PD	N=100
A195	Maia 2003 [195]	Obsessive-compulsive symptoms, obsessive-compulsive disorder, and related disorders in Parkinson's disease	Brazil	Case-control study	PD	N=200
A196	Mamikonyan 2008 [196]	Long-term follow-up of impulse control disorders in Parkinson's disease	UK	Cross-sectional study (2 time points)	PD with ICD	N=15
A197	Marder 2007 [197]	Aripiprazole effects in patients with acute schizophrenia experiencing higher or lower agitation: A post hoc analysis of 4 randomized, placebo-controlled clinical trials	International	Posthoc study of 4 RCTs	Acute schizophrenia under aripiprazole or placebo treatment	N=1187 (790+397)
A198	Maréchal 2019 [198]	Impulsive-Compulsive Behaviours in Belgian-Flemish Parkinson's Disease Patients: A Questionnaire-Based Study	Belgium	Cross-sectional study	PD	N=88

A199	Marín-Lahoz 2018 [199]	Parkinson's Disease: Impulsivity Does Not Cause Impulse Control Disorders but Boosts Their Severity	Spain	Cross-sectional study	PD	N=100
A200	Marín-Lahoz 2019 [200]	Depression as a Risk Factor for Impulse Control Disorders in Parkinson Disease	International	Cohort study	PD	N=354
A201	Marín-Lahoz 2022 [201]	Predicting Impulse Control Disorders in Parkinson Disease through Incentive Biomarkers	Spain	Cohort study	PD	N=94
A202	Marković 2020 [202]	Dynamics of impulsive–compulsive behaviors in early Parkinson's disease: a prospective study	Serbia	Cohort study	Early PD and HCs	N=231 (106+125)
A203	Marques 2018 [203]	Impulse control disorders in Parkinson's disease patients with RLS: a cross sectional-study	France	Cross-sectional study	PD with and without RLS	N=80
A204	Marques 2021 [204]	The urge to move: From restless legs syndrome to impulse control disorders in Parkinson's disease	France	Cross-sectional study	PD with and without ICD	N=50 (17+33)
A205	Marques 2022 [205]	Decision making under uncertainty in Parkinson's disease with Rem sleep behavior disorder	France	Cross-sectional study	PD with and without RBD, HCs	N=80 (40+20+20)
A206	Martinez-Martin 2020 [206]	Impulse control and related behaviors in Parkinson's disease with dementia	Spain	Cross-sectional study	PD with and without dementia	N=529 (85+444)
A207	Martinez-Nunez 2025 [207]	Clinically probable RBD is an early predictor of malignant non-motor Parkinson's disease phenotypes	North America and Europe	Cohort study	PD with and without RBD	N=452 (180+272)
A208	Martini 2018 [208]	Risky decision-making and affective features of impulse control disorders in Parkinson's disease	UK	Case-control study	PD and HCs	N=42 (25+17)
A209	Martini 2019 [209]	Impact of Cognitive Profile on Impulse Control Disorders Presence and Severity in Parkinson's Disease	Italy	Cross-sectional study	PD	N=326
A210	Martinkova 2011 [210]	Impulse control disorders associated with dopaminergic medication in patients with pituitary adenomas	Slovakia	Cross-sectional study	Pituitary adenoma under DA treatment	N=20
A211	McKeown 2020 [211]	The experiences of carers looking after people with Parkinson's disease who exhibit impulsive and compulsive behaviours: An exploratory qualitative study	UK	Qualitative study	Family carers of PD with ICB	N=13
A212	Meira 2021 [212]	Long-term effect of apomorphine infusion in advanced Parkinson's disease: a real-life study	France	Cohort study	PD under apomorphine infusion	N=110
A213	Meng 2023 [213]	Longitudinal cognitive changes in patients with early Parkinson's disease and neuropsychiatric symptoms	USA, Europe, Israel, Australia	Cohort study	De novo PD patients (drug-naïve at BL)	N=423
A214	Merola 2017 [214]	Impulse control behaviors and subthalamic deep brain stimulation in Parkinson disease	Italy	Cohort study	PD with STN DBS	N=150
A215	Miyasaki 2007 [215]	Punding prevalence in Parkinson's disease	Canada	Cross-sectional study	PD	N=291
A216	Mohammad 2021 [216]	Impact of behavioral side effects on the management of Parkinson patients treated with dopamine agonists	USA	Patient records review	PD with DA-AEs	N=156

A217	Mohammad 2024 [217]	Impulse Control Disorders in Southern Iraqi Patients Medicated With Cabergoline for Prolactinoma	Iraq	Case-control study	Prolactinoma under cabergoline therapy and HCs	N=60 (30+30)
A218	Monaco 2018 [218]	Prevalence of impulsive-compulsive symptoms in elderly Parkinson's disease patients: A case-control study	Italy	Case-control study	Elderly PD and HCs	N=220 (115+105)
A219	Moore 2014 [219]	Reports of pathological gambling, hypersexuality, and compulsive shopping associated with dopamine receptor agonist drugs	International	Pharmacovigilance study	ICD-AE reports	N=1580
A220	Morgante 2016 [220]	Impulsive-compulsive behaviors in parkin -associated Parkinson disease	Italy	Case-control study	Early-onset PD with and without PARK2 mutations	N=48 (22+26)
A221	Morgante 2021 [221]	Levodopa–carbidopa intrajejunal infusion in Parkinson’s disease: untangling the role of age	Italy	Case-control study	PD with LCIG late elderly or not	N=50 (25+25)
A222	Morrow 2023 [222]	Examining the link between impulse control disorder and antidepressant use in Parkinson's disease	USA	Cross-sectional study	PD	N=200
A223	Morrow 2024 [223]	The Association of Antidepressant Use and Impulse Control Disorder in Parkinson's Disease	North America, Europe, Africa	Cohort study	Early PD, medication-naïve or minimal treatment at BL	N=1045
A224	Moum 2012 [224]	Effects of STN and GPi deep brain stimulation on impulse control disorders and dopamine dysregulation syndrome	USA	Patient records review	PD with STN/GPi DBS	N=159
A225	Nadeau 2018 [225]	Gambling Habits of People With Parkinson's Disease: An Exploratory Study	USA	Mixed-methods cross-sectional study	PD with and without PG and caregivers	N=33 (15+10+8)
A226	Nardone 2014 [226]	Repetitive transcranial magnetic stimulation transiently reduces punding in Parkinson's disease: A preliminary study	Italy	Intervention study other than RCT	PD with punding and HCs	N=13 (4+9)
A227	Nissen 2012 [227]	Duration of L-dopa and dopamine agonist monotherapy in Parkinson's disease	UK	Cohort study	PD	N=345
A228	Okai 2013 [228]	Trial of CBT for impulse control behaviors affecting Parkinson patients and their caregivers	UK	RCT	PD with ICD and carers	N=90 (45+45)
A229	Okai 2015 [229]	Predictors of response to a cognitive behavioral intervention for impulse control behaviors in Parkinson's disease	UK	Intervention study other than RCT	PD with ICB	N=42
A230	Olanow 2020 [230]	Apomorphine sublingual film for off episodes in Parkinson's disease: a randomised, double-blind, placebo-controlled phase 3 study	North America	RCT	PD under sublingual apomorphine or placebo	N=109 (54+55)
A231	Olley 2015 [231]	Dopaminergic Medication in Parkinson's Disease and Problem Gambling	Australia	Mixed-methods case-control study	PD with and without PG	N=40 (20+20)
A232	Ondo 2008 [232]	Predictors of impulsivity and reward seeking behavior with dopamine agonists	USA	Cross-sectional study	PD and RLS	N=300

A233	O'Sullivan 2010 [233]	Excessive hoarding in Parkinson's disease	UK	Case-control study	PD with and without ICBs and HCs	N=150 (39+61+50)
A234	O'Sullivan 2011 [234]	Sleep disturbance and impulsive-compulsive behaviours in Parkinson's disease	UK	Case-control study	PD with and without ICBs and HCs	N=140 (30+62+48)
A235	Ozdeniz Varan 2023 [235]	Effect of Dopaminergic Therapy on Impulse Control Disorders in Patients With a Prolactinoma	Turkey	Cross-sectional study	Prolactinoma with and without DA treatment and HCs	N=81 (31+20+30)
A236	Ozer 2024 [236]	Daytime impulsiveness, attention, and learning in the restless legs syndrome	Turkey	Cross-sectional study	RLS and HCs	N=55 (23+22)
A237	Ozkaya 2020 [237]	Patients with acromegaly might not be at higher risk for dopamine agonist-induced impulse control disorders than those with prolactinomas	Turkey	Cross-sectional study	Acromegaly, prolactinoma, non-functioning pituitary adenoma, HCs	N=150 (40+40+38+32)
A238	Paliwal 2019 [238]	Subjective estimates of uncertainty during gambling and impulsivity after subthalamic deep brain stimulation for Parkinson's disease	Australia	Cohort study	PD with STN DBS	N=38
A239	Pallanti 2010 [239]	Complex repetitive behavior: Punding after bilateral subthalamic nucleus stimulation in Parkinson's disease	Italy	Cross-sectional study	PD with STN DBS	N=24
A240	Papay 2011 [240]	Patient versus informant reporting of ICD symptoms in Parkinson's disease using the QUIP: Validity and variability	USA	Cross-sectional study	PD and informants	N=142 (71+71)
A241	Papay 2014 [241]	Naltrexone for impulse control disorders in Parkinson disease: a placebo-controlled study	USA	RCT	PD with ICD treated with naltrexone or placebo	N=50 (26+24)
A242	Paul 2020 [242]	Gender Differences in Impulse Control Disorders and Related Behaviors in Patients with Parkinson's Disease and its Impact on Quality of Life	India	Cross-sectional study	PD	N=102
A243	Paul 2023 [243]	Impulse-control disorders and restless leg syndrome in Parkinson's Disease: Association or coexistence	India	Cross-sectional study	PD	N=95
A244	Perez-Lloret 2012 [244]	Prevalence and pharmacological factors associated with impulse-control disorder symptoms in patients with parkinson disease	France	Cross-sectional study	PD and unmedicated stroke as controls	N=255 (203+52)
A245	Pettorruso 2016 [245]	Punding in non-demented Parkinson's disease patients: Relationship with psychiatric and addiction spectrum comorbidity	Italy	Cross-sectional study	PD with and without punding	N=155 (25+130)
A246	Phu 2014 [246]	Effect of impulse control disorders on disability and quality of life in Parkinson's disease patients	Australia	Cross-sectional study	PD	N=100
A247	Picillo 2021 [247]	Levodopa Versus Dopamine Agonist after Subthalamic Stimulation in Parkinson's Disease	Canada	Intervention study other than RCT	PD with LD or DA monotherapy post-STN DBS	N=35 (17+18)
A248	Pineau 2016 [248]	Executive functioning and risk-taking behavior in Parkinson’s disease patients with impulse control disorders	France	Case-control study	PD with and without ICD	N=37 (17+20)
A249	Poletti 2013 [249]	A single-center, cross-sectional prevalence study of impulse control disorders in Parkinson disease: Association with dopaminergic drugs	Italy	Cross-sectional study	PD	N=805
A250	Pondal 2013 [250]	Clinical features of dopamine agonist withdrawal syndrome in a movement disorders clinic	Canada	Patient records review	PD under DA withdrawal	N=84

A251	Pontieri 2015 [251]	Sociodemographic, neuropsychiatric and cognitive characteristics of pathological gambling and impulse control disorders NOS in Parkinson's disease	Italy	Cross-sectional study	PD	N=155
A252	Pontone 2006 [252]	Clinical features associated with impulse control disorders in Parkinson disease	USA	Cohort study	PD	N=100
A253	Porta 2016 [253]	Long-term safety and effectiveness of pramipexole in tetrahydrobiopterin deficiency	Italy	Cohort study	Tetrahydrobiopterin (BH4) deficiency under PPX treatment	N=7
A254	Pourcher 2010 [254]	Compulsive habits in restless legs syndrome patients under dopaminergic treatment	Canada	Cross-sectional study (3 time points)	RLS	N=97
A255	Rábano-Suárez 2021 [255]	Impulse Control Disorders in Parkinson's Disease: Has COVID-19 Related Lockdown Been a Trigger?	Spain	Patient records review	PD	N=249
A256	Rabinak 2010 [256]	Dopamine agonist withdrawal syndrome in parkinson disease	USA	Cohort study	PD	N=93
A257	Rana 2013 [257]	Factors associated with the development of impulse compulsive disorders in Parkinson patients	Canada	Cross-sectional study	PD	N=140
A258	Redenšek 2020 [258]	Clinical and clinical-pharmacogenetic models for prediction of the most common psychiatric complications due to dopaminergic treatment in Parkinson's disease	Slovenia	Cross-sectional study	PD	N=214
A259	Ren 2023 [259]	Evaluation of risk factors for impulse control disorder in Parkinson’s disease in northern China	China	Cross-sectional study	PD with and without ICD	N=285 (33+252)
A260	Ricciardi 2018 [260]	Can we predict development of impulsive-compulsive behaviours in Parkinson's disease?	North America, Europe, Australia	Case-control study within cohort	PD with and without ICB, HCs	N=126 (42+42+42)
A261	Ricciardi 2023 [261]	Dyskinesia and impulsive compulsive behaviour in Parkinson's disease are not related: Insights from a study with a wearable sensor	UK	Cross-sectional study	PD with and without ICD	N=89 (36+53)
A262	Rieu 2016 [262]	Impact of Mood and Behavioral Disorders on Quality of Life in Parkinson's disease	France	Cross-sectional study	PD	N=136
A263	Rizos 2016 [263]	A European multicentre survey of impulse control behaviours in Parkinson's disease patients treated with short- and long-acting dopamine agonists	UK, Spain, Denmark and Romania	Cross-sectional study	PD under DA treatment	N=425
A264	Rodríguez-Violante 2014 [264]	Impulse control and related disorders in Mexican Parkinson's disease patients	Mexico	Cross-sectional study	PD and HCs	N=450 (300+150)
A265	Rodríguez-Violante 2021 [265]	Assessment of therapeutic strategies for management of impulse control disorder in Parkinson's disease	Mexico	Cohort study	PD	N=132
A266	Rossi 2017 [266]	Measures of impulsivity in Parkinson's disease decrease after DBS in the setting of stable dopamine therapy	USA	Cohort study	PD with STN/GPi DBS	N=37

A267	Saadat 2020 [267]	Psychiatric symptoms in patients with Parkinson's disease and caregivers	Iran	Cross-sectional study	PD and caregivers	N=250
A268	Sachdeva 2014 [268]	Clinical profile of compulsive sexual behaviour and paraphilia in Parkinson's disease	UK	Case-control study	PD with CSB, other ICD, PD controls	N=73 (20+11+42)
A269	Sahin 2023 [269]	A Genetic Assessment of Dopamine Agonist-Induced Impulse Control Disorder in Patients With Prolactinoma	Turkey	Cross-sectional study	Prolactinoma under cabergoline therapy	N=72
A270	Sanjan 2023 [270]	Impulse Control Disorders with Short-term Use of Cabergoline in Macroprolactinomas: A Prospective Study with a Brief Review of Literature	India	Cohort study	Macroprolactinoma under cabergoline treatment and with non-functioning pituitary adenomas without dopaminergic treatment	N=30 (15+15)
A271	Santangelo 2009 [271]	Cognitive dysfunctions and Pathological gambling in patients with Parkinson's disease	Italy	Case-control study	PD with and without PG	N=30 (15+15)
A272	Santin 2021 [272]	Impact of Subthalamic Deep Brain Stimulation on Impulse Control Disorders in Parkinson's Disease: A Prospective Study	France	Cohort study	PD with STN DBS	N=217
A273	Santos García 2019 [273]	COPPADIS-2015 (COhort of Patients with PArkinson's DIsease in Spain, 2015): an ongoing global Parkinson's disease project about disease progression with more than 1000 subjects included. Results from the baseline evaluation	Spain	Cohort study	PD	N=694
A274	Santos-García 2021 [274]	Depression is Associated with Impulse-compulsive Behaviors in Parkinson's disease	Spain	Cross-sectional study	PD	N=613
A275	Santos-García 2021 [275]	Sleep Problems Are Related to a Worse Quality of Life and a Greater Non-Motor Symptoms Burden in Parkinson's Disease	Spain	Cross-sectional study	PD and HCs	N=887 (681+206)
A276	Sauerbier 2021 [276]	Predictors of short-term impulsive and compulsive behaviour after subthalamic stimulation in Parkinson disease	Germany and UK	Cohort study	PD with STN DBS	N=55
A277	Scavone 2020 [277]	Drugs-Induced Pathological Gambling: An Analysis of Italian Spontaneous Reporting System	Italy	Pharmacovigilance study	PG-AEs under dopaminergic medication	N=94
A278	Schreglmann 2012 [278]	Transdermal rotigotine causes impulse control disorders in patients with restless legs syndrome	Switzerland	Patient records review	RLS under rotigotine treatment	N=28
A279	Scott 2020 [279]	Co-occurrence of apathy and impulse control disorders in Parkinson disease	USA	Cross-sectional study	PD	N=887
A280	Scott 2022 [280]	From pleasure to punding: Distinct patterns of anhedonia and impulsivity linked to motivational disturbances in Parkinson disease	USA	Cross-sectional study	PD	N=64
A281	Scott 2024 [281]	Co-Occurrence of Apathy and Impulse Control Disorders in Parkinson Disease: Variation across Multiple Measures	USA	Cross-sectional study	PD	N=97
A282	Scullin 2013 [282]	Sleep and impulsivity in Parkinson's disease	USA	Cross-sectional study	PD	N=143
A283	Shapiro 2007 [283]	The four as associated with pathological Parkinson disease gamblers: Anxiety, anger, age, and agonists	USA	Cross-sectional study	PD	N=127
A284	Sharma 2015 [284]	Impulse control disorders and related behaviours (ICD-RBs) in Parkinson's disease patients: Assessment using "questionnaire for impulsive-compulsive disorders in Parkinson's disease" (QUIP)	India	Cross-sectional study	PD	N=299

A285	Simoni 2020 [285]	Impulse Control Disorders and Levodopa-Induced Dyskinesias in Parkinson's Disease: Pulsatile versus Continuous Dopaminergic Stimulation	Italy	Cross-sectional study	PD	N=117
A286	Şimşek Erdem 2023[286]	Neuropsychiatric Effects of Bilateral Subthalamic Nucleus Deep Brain Stimulation in Parkinson's Disease: Results at the 12-Month Follow-up	Turkey	Cohort study	PD with STN DBS	N=22
A287	Singh 2007 [287]	Risk factors for pathologic gambling and other compulsions among Parkinson's disease patients taking dopamine agonists	USA	Cross-sectional study	PD	N=300
A288	Siri 2010 [288]	Psychiatric symptoms in Parkinson's disease assessed with the SCL-90R self-reported questionnaire	Italy	Cross-sectional study	PD	N=486
A289	Siri 2010 [289]	Cognitive status of patients with Parkinson's disease and pathological gambling	Italy	Case-control study	PD with and without PG	N=63 (21+42)
A290	Siri 2015 [290]	Long-term cognitive follow-up of Parkinson's disease patients with impulse control disorders	Italy	Cohort study	PD with and without ICD	N=80 (40+40)
A291	Smith 2016 [291]	Incident impulse control disorder symptoms and dopamine transporter imaging in Parkinson disease	International	Cohort study	PD	N=320
A292	Sohtaoĝlu 2010 [292]	Long term follow-up of Parkinson's disease patients with impulse control disorders	Turkey	Cohort study	PD with ICD	N=22
A293	Solla 2011 [293]	Behavioral, neuropsychiatric and cognitive disorders in Parkinson's disease patients with and without motor complications	Italy	Cross-sectional study	PD	N=349
A294	Solla 2012 [294]	Gender differences in motor and non-motor symptoms among Sardinian patients with Parkinson's disease	Italy	Case-control study	PD and HCs	N=288 (156+132)
A295	Somma 2022 [295]	Psychiatric Symptoms in Parkinson's Disease Patients before and One Year after Subthalamic Nucleus Deep Brain Stimulation Therapy: Role of Lead Positioning and Not of Total Electrical Energy Delivered	Italy	Cohort study	PD with STN DBS	N=14
A296	Somme 2014 [296]	Impulse control and repetitive behaviors in Parkinson's disease - Are there differences in the relation to dopamine agonist treatment?	Spain	Cross-sectional study	PD	N=91
A297	Staubo 2024 [297]	Dopamine agonist serum concentrations and impulse control disorders in Parkinson's disease	Norway	Case-control study	PD with and without ICDs	N=46 (19+27)
A298	Staubo 2025 [298]	Impulse control and correlation to dopamine agonist serum concentrations in people with Parkinson's disease	Norway	Cross-sectional study	PD	N=100
A299	Sulu 2023 [299]	Risk of impulse control disorders in patients with Cushing's disease: do not blame cabergoline but do not give up caution	Turkey	Part A: Cohort study<br>Part B: Case-control study	Cushing's disease with and without cabergoline treatment	Part A: N=14, Part B: N=68 (34+34)
A300	Takahashi 2022 [300]	Validation of the Japanese Version of the Questionnaire for Impulsive-Compulsive Disorders in Parkinson's Disease-Rating Scale (QUIP-RS)	Japan	Cross-sectional study	PD	N=159
A301	Tanaka 2013 [301]	Impulsive compulsive behaviors in Japanese Parkinson's disease patients and utility of the Japanese version of the Questionnaire for Impulsive-Compulsive Disorders in Parkinson's disease	Japan	Cross-sectional study	PD	N=118

A302	Tang 2024 [302]	Excessive Daytime Sleepiness as a Risk Factor for Impulse Control Disorders in Parkinson’s Disease	International	Cohort study	PD without ICD at BL	N=260
A303	Tayim 2024 [303]	A qualitative study exploring the clinical phenomenology and impact of hypersexuality in patients with Parkinson's Disease	UK	Qualitative study	PD with CSB	N=9
A304	Tayim 2025 [304]	Impact of hypersexuality on spousal carers of patients with Parkinson's disease and frontotemporal dementia: a qualitative study	UK	Qualitative study	Carers of PD with CSB or behavioural-variant frontotemporal dementia with CSB	N=8
A305	Terenzi 2018 [305]	Reward sensitivity in Parkinson's patients with binge eating	Italy	Cross-sectional study	PD with and without BE, HCs	N=51 (16+15+20)
A306	Thomas 2010 [306]	Pathological gambling in parkinson disease is reduced by amantadine	Italy	Intervention study other than RCT	PD with PG	N=17
A307	Thomson 2021 [307]	Changes in Personality Associated with Deep Brain Stimulation: a Qualitative Evaluation of Clinician Perspectives	Australia	Qualitative study	DBS clinicians	N=16
A308	Todorova 2015 [308]	Infusion Therapies and Development of Impulse Control Disorders in Advanced Parkinson Disease: Clinical Experience after 3 Years' Follow-up	UK	Cohort study	PD with apomorphine or LCIG infusion	N=60 (41+19)
A309	Tolosa 2021 [309]	The Parkinson's Real-World Impact Assessment (PRISM) Study: A European Survey of the Burden of Parkinson's Disease in Patients and their Carers	France, Germany, Italy, Portugal, Spain, UK	Cross-sectional study	PD and carers	N=1117 (861+256)
A310	Toś 2023 [310]	Impulse Control Disorders in the Polish Population of Patients with Parkinson's Disease	Poland	Cross-sectional study	PD	N=135
A311	Toś 2024 [311]	Impulse Control Disorders in Parkinson’s Disease and Atypical Parkinsonian Syndromes—Is There a Difference?	Poland	Cohort study	PD and atypical parkinsonism	N=212 (185+36)
A312	Trovini 2025 [312]	Partial Dopamine D2/3 Agonists and Dual Disorders: A Retrospective-Cohort Study in a Real-World Clinical Setting on Patients with Schizophrenia Spectrum Disorders and Cannabis Use Disorder	Italy	Cohort study	Schizophrenia comorbid with cannabis use disorder	N=96
A313	Turcano 2024 [313]	Impulse control disorders and use of dopamine agonists in early onset Parkinson’s disease	USA	Cohort study	Early-onset PD	N=831
A314	Umemura 2011 [314]	Complications of subthalamic nucleus stimulation in Parkinson's disease	Japan	Cohort study	PD with STN DBS	N=180
A315	Valença 2013 [315]	Past smoking and current dopamine agonist use show an independent and dose-dependent association with impulse control disorders in Parkinson's disease	Brazil	Case-control study	PD and HCs	N=363 (152+212)
A316	Vargas 2019 [316]	Impulse control symptoms in patients with Parkinson's disease: The influence of dopaminergic agonist	Brazil	Case-control study	PD with and without DA treatment, HCs	N=437 (79+128+230)

A317	Varshney 2024 [317]	Nonmotor Symptoms and Impulse Control Disorders in Parkinson's Disease Patients in India: A Cross-sectional Study	India	Cross-sectional study	PD	N=32
A318	Veiga 2025 [318]	Are impulsive compulsive behaviors associated with levodopa-induced dyskinesia? A Brazilian cross-sectional study	Brazil	Cross-sectional study	Young PD	N=90
A319	Vela 2016 [319]	The high prevalence of impulse control behaviors in patients with early-onset Parkinson's disease: A cross-sectional multicenter study	Spain	Cross-sectional study	Early-onset PD and HCs	N=174 (87+87)
A320	Verbaan 2009 [320]	Psychotic and compulsive symptoms in Parkinson's disease	Netherlands	Cross-sectional study	PD	N=353
A321	Vitale 2011 [321]	Comparative neuropsychological profile of pathological gambling, hypersexuality, and compulsive eating in Parkinson's disease	Italy	Case-control study	PD with and without ICDs	N=77 (63+14)
A322	Voon 2006 [322]	Prevalence of repetitive and reward-seeking behaviors in Parkinson disease	Canada	Cross-sectional study	PD	N=297
A323	Voon 2006 [323]	Prospective prevalence of pathologic gambling and medication association in Parkinson disease	Canada	Cross-sectional study	PD	N=297
A324	Voon 2007 [324]	Factors associated with dopaminergic drug-related pathological gambling in Parkinson disease	Canada	Case-control study	PD with and without PG	N=63 (21+42)
A325	Voon 2010 [325]	Impulsive choice and response in dopamine agonist-related impulse control behaviors	USA	Case-control study	PD with and without ICDs, HCs	N=44 (14+14+16)
A326	Voon 2011 [326]	Impulse control disorders in Parkinson disease: A multicenter case–control study	North America	Case-control study	PD with and without ICDs	N=564 (282+282)
A327	Voon 2011 [327]	Frequency of impulse control behaviours associated with dopaminergic therapy in restless legs syndrome	Germany	Cross-sectional study	RLS	N=140
A328	Wang 2016 [328]	A survey of impulse control disorders in Parkinson's disease patients in Shanghai area and literature review	China	Cross-sectional study	PD	N=217
A329	Waskowiak 2022 [329]	Trait Anxiety as a Risk Factor for Impulse Control Disorders in de novo Parkinson's Disease	North America and Europe	Cohort study	PD	N=330
A330	Weintraub 2006 [330]	Association of dopamine agonist use with impulse control disorders in Parkinson disease	USA	Cross-sectional study	PD	N=272
A331	Weintraub 2010 [331]	Amantadine use associated with impulse control disorders in Parkinson disease in cross-sectional study	North America	Cross-sectional study	PD	N=3085
A332	Weintraub 2010 [332]	Impulse control disorders in Parkinson disease: A cross-sectional study of 3090 patients	North America	Cross-sectional study	PD	N=3090
A333	Weintraub 2013 [333]	Screening for impulse control symptoms in patients with de novo Parkinson disease: A casecontrol study	International	Case-control study	PD and HCs	N=311 (168+143)
A334	Wicks 2009 [334]	Pathological gambling amongst Parkinson's disease and ALS patients in an online community (PatientsLikeMe.com)	International	Cross-sectional study	PD and ALS	N=444 (208+236)
A335	Wirth 2024 [335]	Prognosis of impulse control disorders in Parkinson's disease: a prospective controlled study	France	Case-control study	PD with and without ICD	N=149 (78+71)
A336	Wolfschlag 2021 [336]	Increased risk for developing gambling disorder under the treatment with pramipexole, ropinirole, and aripiprazole: A nationwide register study in Sweden	Sweden	Register study	Patients with PG and HCs	N=11,067

A337	Wolfschlag 2024 [337]	Impulse control disorders in Parkinson's disease: a national Swedish registry study on high-risk treatments and vulnerable patient groups	Sweden	Register study	PD	N=55,235
A338	Wolfschlag 2025 [338]	Recognition, management, and patient perspectives of impulsive-compulsive disorders in Parkinson's disease	Sweden	Cross-sectional study	PD with ICB	N=170
A339	Wu 2014 [339]	Problematic Internet use in Parkinson's disease	UK	Cross-sectional study	PD with and without ICDs and healthy controls	N=68
A340	Wu 2024 [340]	Impulse control and related behavioral disorders (ICRD) in Idiopathic Parkinson's Disease treated with different dopamine agonists in Hong Kong: Is any dopamine agonist better?	China (Hong Kong)	Cohort study	PD	N=311
A341	Xu 2021 [341]	Validation of the Chinese Version of the Questionnaire for Impulsive-Compulsive Disorders in Parkinson's Disease	China	Cross-sectional study	PD	N=207
A342	Ye 2024 [342]	Associations between Sleep Disorders and Impulsive-Compulsive Behaviors in Parkinson's Disease: A Prospective Cohort Study	International	Cohort study	PD	N=807
A343	Yildirim 2020 [343]	Decision-making and impulse-control disorders in parkinson's disease: Influence of dopaminergic treatment	Turkey	Cross-sectional study	PD and HCs	N=86 (39+37)
A344	Zahodne 2011 [344]	Binge eating in Parkinson's disease: Prevalence, correlates and the contribution of deep brain stimulation	USA	Cross-sectional study	PD	N=96
A345	Zazu 2021 [345]	Do cariprazine and brexpiprazole cause impulse control symptoms? A case/non-case study	International	Pharmacovigilance study	ICD-AE reports under cariprazine or brexpiprazole treatment	N=7 in 2,276,306, N=7 in 1,559,281
A346	Zhang 2017 [346]	Clinical characteristics of impulse control and related disorders in Chinese Parkinson's disease patients	China	Cross-sectional study	PD	N=142
A347	Zhang 2024 [347]	Impulse control behaviors and apathy commonly co-occur in de novo Parkinson's disease and predict the incidence of levodopa-induced dyskinesia	International	Cohort study	De novo PD and HCs	N=617 (422+195)
A348	Zhong 2016 [348]	The relationship between specific cognitive defects and burden of care in Parkinson's disease	Australia	Cross-sectional study	PD and caregivers	N=100
A349	Zhu 2023 [349]	Assessing impulse control behaviors in early Parkinson's disease: a longitudinal study	International	Cohort study	PD and HCs	N=586 (401+185)

1. Abbes M, Lhommée E, Thobois S, et al. Subthalamic stimulation and neuropsychiatric symptoms in Parkinson's disease: Results from a long-term follow-up cohort study. *Journal of Neurology, Neurosurgery and Psychiatry* 2018; 89: 836-843. DOI: 10.1136/jnnp-2017-316373.
2. Abosch A, Gupte A, Eberly LE, et al. Impulsive behavior and associated clinical variables in parkinson's disease. *Psychosomatics* 2011; 52: 41. DOI: 10.1016/j.psym.2010.11.013.
3. Ahearn DJ, McDonald K, Barraclough M, et al. An exploration of apathy and impulsivity in Parkinson disease. *Curr Gerontol Geriatr Res* 2012; 2012: 390701. DOI: 10.1155/2012/390701.
4. Almalki MH, Alsuraikh MA, Almalki E, et al. Impulse control disorders in patients with dopamine agonist-treated pituitary adenomas: a cross-sectional multicenter study. *Pituitary* 2024; 27: 197-203. DOI: 10.1007/s11102-024-01383-2.
5. Alvarado-Bolaños A, Cervantes-Arriaga A, Rodríguez-Violante M, et al. Impact of neuropsychiatric symptoms on the quality of life of subjects with Parkinson's disease. *Journal of Parkinson's Disease* 2015; 5: 541-548. DOI: 10.3233/JPD-150597.

6. Amami P, Dekker I, Piacentini S, et al. Impulse control behaviours in patients with Parkinson's disease after subthalamic deep brain stimulation:De novo cases and 3-year follow-up. *Journal of Neurology, Neurosurgery and Psychiatry* 2015; 86: 562-564. DOI: 10.1136/jnnp-2013-307214.
7. Amstutz D, Petermann K, Sousa M, et al. Impulse Control Disorders and Effort-Based Decision-Making in Parkinson's Disease Patients with Subthalamic Nucleus Deep Brain Stimulation. *Movement Disorders Clinical Practice* 2025. DOI: 10.1002/mdc3.14318.
8. Angioletti L, Campanella S and Balconi M. Metacognition deficits and impulsivity in Parkinson's Disease patients with and without gambling behavior: A pilot study. *Neurology Psychiatry and Brain Research* 2020; 36: 88-95. DOI: 10.1016/j.npbr.2020.04.002.
9. Antonini A, Chaudhuri KR, Boroojerdi B, et al. Impulse control disorder related behaviours during long-term rotigotine treatment: a post hoc analysis. *European Journal of Neurology* 2016; 23: 1556-1565. DOI: 10.1111/ene.13078.
10. Antonini A, Barone P, Bonuccelli U, et al. ICARUS study: Prevalence and clinical features of impulse control disorders in Parkinson's disease. *Journal of Neurology, Neurosurgery and Psychiatry* 2017; 88: 317-324. DOI: 10.1136/jnnp-2016-315277.
11. Aoki R, Shiraishi M, Mikami K, et al. Deterioration of postural deformity in Parkinson's disease patients with punding and hobbyism. *Journal of Clinical Neuroscience* 2019; 69: 179-183. DOI: 10.1016/j.jocn.2019.07.069.
12. Ardouin C, Voon V, Worbe Y, et al. Pathological gambling in Parkinson's disease improves on chronic subthalamic nucleus stimulation. *Movement Disorders* 2006; 21: 1941-1946. DOI: 10.1002/mds.21098.
13. Ataoglu EE, Bozdag Y and Tokcaer AB. Impulse control disorders related cognitive and behavioral features in Parkinson's disease. *TURKISH JOURNAL OF NEUROLOGY* 2024; 30: 125-133. DOI: 10.55697/tnd.2024.109.
14. Aumann MA, Stark AJ, Hughes SB, et al. Self-reported rates of impulsivity in Parkinson’s Disease. *Annals of Clinical and Translational Neurology* 2020; 7: 437-448. DOI: 10.1002/acn3.51016.
15. Auyeung M, Tsoi TH, Tang WK, et al. Impulse control disorders in Chinese Parkinson's disease patients: The effect of ergot derived dopamine agonist. *Parkinsonism and Related Disorders* 2011; 17: 635-637. DOI: 10.1016/j.parkreldis.2011.06.001.
16. Avanzi M, Baratti M, Cabrini S, et al. Prevalence of pathological gambling in patients with Parkinson's disease. *Movement Disorders* 2006; 21: 2068-2072. DOI: 10.1002/mds.21072.
17. Ávila A, Cardona X, Martín-Baranera M, et al. Impulsive and compulsive behaviors in Parkinson's disease: A one-year follow-up study. *Journal of the Neurological Sciences* 2011; 310: 197-201. DOI: 10.1016/j.jns.2011.05.044.
18. Azmin S, Tan EL, Ruzyanei NJN, et al. Impulse control behaviours in a Malaysian Parkinson’s disease population. *Neurology Asia* 2016; 21: 137-143.
19. Baig F, Kelly MJ, Lawton MA, et al. Impulse control disorders in Parkinson disease and RBD: A longitudinal study of severity. *Neurology* 2019; 93: E675-E687. DOI: 10.1212/WNL.0000000000007942.
20. Bancos I, Nannenga MR, Bostwick JM, et al. Impulse control disorders in patients with dopamine agonist-treated prolactinomas and nonfunctioning pituitary adenomas: A case-control study. *Clinical Endocrinology* 2014; 80: 863-868. DOI: 10.1111/cen.12375.
21. Barake M, Evins AE, Stoeckel L, et al. Investigation of impulsivity in patients on dopamine agonist therapy for hyperprolactinemia: A pilot study. *Pituitary* 2014; 17: 150-156. DOI: 10.1007/s11102-013-0480-6.
22. Barbosa P, Lees AJ, Magee C, et al. A Retrospective Evaluation of the Frequency of Impulsive Compulsive Behaviors in Parkinson’s Disease Patients Treated with Continuous Waking Day Apomorphine Pumps. *Movement Disorders Clinical Practice* 2017; 4: 323-328. DOI: 10.1002/mdc3.12416.
23. Barbosa P, Djamshidian A, Lees AJ, et al. The Outcome of Dopamine Dysregulation Syndrome in Parkinson's Disease: A Retrospective Postmortem Study. *Movement Disorders Clinical Practice* 2018; 5: 519-522. DOI: 10.1002/mdc3.12671.
24. Barbosa PM, Grippe T, Lees AJ, et al. Compulsive sexual behaviour in Parkinson's disease is associated with higher doses of levodopa. *Journal of Neurology, Neurosurgery and Psychiatry* 2018; 89: 1121-1123. DOI: 10.1136/jnnp-2017-317298.
25. Barbosa P, Hapuarachchi B, Djamshidian A, et al. Lower nucleus accumbens α-synuclein load and D3 receptor levels in Parkinson's disease with impulsive compulsive behaviours. *Brain* 2019; 142: 3580-3591. DOI: 10.1093/brain/awz298.
26. Barbosa P, Djamshidian A, Lees AJ, et al. The prevalence of impulsive compulsive behaviors in patients treated with apomorphine infusion: A retrospective analysis. *Arquivos de Neuro-Psiquiatria* 2022; 80: 56-61. DOI: 10.1590/0004-282X-ANP-2020-0522.

27. Barbosa P, O'Sullivan SS, Joyce E, et al. Neuropsychiatric Features of Punding and Hobbyism in Parkinson's Disease. *Movement Disorders Clinical Practice* 2022; 9: 82-86. DOI: 10.1002/mdc3.13363.
28. Barone P, Antonini A, Stanzione P, et al. Risk factors for impulse control disorders and related behaviors in Parkinson's disease: secondary analyses of the ICARUS study. *J Drug Assess* 2019; 8: 159-166. DOI: 10.1080/21556660.2019.1675670.
29. Bastiaens J, Dorfman BJ, Christos PJ, et al. Prospective cohort study of impulse control disorders in Parkinson's disease. *Movement Disorders* 2013; 28: 327-333. DOI: 10.1002/mds.25291.
30. Baumann-Vogel H, Valko PO, Eisele G, et al. Impulse control disorders in Parkinson's disease: Don't set your mind at rest by self-assessments. *European Journal of Neurology* 2015; 22: 603-609. DOI: 10.1111/ene.12646.
31. Bayard S, Langenier MC and Dauvilliers Y. Decision-making, reward-seeking behaviors and dopamine agonist therapy in restless legs syndrome. *Sleep* 2013; 36: 1501-1507. DOI: 10.5665/sleep.3044.
32. Bayard S, Dauvilliers Y, Yu H, et al. Impulse control disorder and rapid eye movement sleep behavior disorder in Parkinson's disease. *Parkinsonism and Related Disorders* 2014; 20: 1411-1414. DOI: 10.1016/j.parkreldis.2014.09.020.
33. Beccuti G, Guaraldi F, Natta G, et al. Increased prevalence of impulse control disorder symptoms in endocrine diseases treated with dopamine agonists: a cross-sectional study. *Journal of Endocrinological Investigation* 2021; 44: 1699-1706. DOI: 10.1007/s40618-020-01478-0.
34. Bentivoglio AR, Baldonero E, Ricciardi L, et al. Neuropsychological features of patients with Parkinson's disease and impulse control disorders. *Neurological Sciences* 2013; 34: 1207-1213. DOI: 10.1007/s10072-012-1224-5.
35. Benussi A, Alberici A, Cantoni V, et al. Modulating risky decision-making in Parkinson's disease by transcranial direct current stimulation. *European Journal of Neurology* 2017; 24: 751-754. DOI: 10.1111/ene.13286.
36. Bermejo PE, Ruiz-Huete C and Anciones B. Zonisamide in managing impulse control disorders in Parkinson's disease. *Journal of Neurology* 2010; 257: 1682-1685. DOI: 10.1007/s00415-010-5603-7.
37. Bharmal A, Lu C, Quickfall J, et al. Outcomes of patients with Parkinson disease and pathological gambling. *The Canadian journal of neurological sciences Le journal canadien des sciences neurologiques* 2010; 37: 473-477. DOI: 10.1017/S0317167100010489.
38. Binck S, Pauly C, Vaillant M, et al. Contributing Factors and Evolution of Impulse Control Disorder in the Luxembourg Parkinson Cohort. *Frontiers in Neurology* 2020; 11. DOI: 10.3389/fneur.2020.578924.
39. Biundo R, Weis L, Abbruzzese G, et al. Impulse control disorders in advanced Parkinson's disease with dyskinesia: The ALTHEA study. *Movement Disorders* 2017; 32: 1557-1565. DOI: 10.1002/mds.27181.
40. Bostwick JM, Hecksel KA, Stevens SR, et al. Frequency of new-onset pathologic compulsive gambling or hypersexuality after drug treatment of idiopathic Parkinson disease. *Mayo Clinic Proceedings* 2009; 84: 310-316. DOI: 10.4065/84.4.310.
41. Brigas HC, Rodrigues AM, Morgadinho A, et al. Living with Parkinson's disease in Portugal: Findings from PRISM study. *Parkinsonism and Related Disorders* 2024; 128. DOI: 10.1016/j.parkreldis.2024.107124.
42. Bril A, Perez-Lloret S, Rossi M, et al. A multifactorial study on nutritional status, binge eating and physical activity as main factors directly influencing body weight in Parkinson’s disease. *npj Parkinson's Disease* 2017; 3. DOI: 10.1038/S41531-017-0018-0.
43. Cabrini S, Baratti M, Bonfà F, et al. Preliminary evaluation of the DDS-PC inventory: A new tool to assess impulsive - Compulsive behaviours associated to dopamine replacement therapy in Parkinson's disease. *Neurological Sciences* 2009; 30: 307-313. DOI: 10.1007/s10072-009-0101-3.
44. Callesen MB, Weintraub D, Damholdt MF, et al. Impulsive and compulsive behaviors among Danish patients with Parkinson's disease: Prevalence, depression, and personality. *Parkinsonism and Related Disorders* 2014; 20: 22-26. DOI: 10.1016/j.parkreldis.2013.09.006.
45. Callesen MB and Damholdt MF. Phenomenology and gender characteristics of hobbyism and punding in Parkinson's disease: A self-report study. *Basal Ganglia* 2017; 9: 1-6. DOI: 10.1016/j.baga.2017.06.002.
46. Canesi M, Lavolpe S, Cereda V, et al. Hypomania, depression, euthymia: New evidence in Parkinson’s disease. *Behavioural Neurology* 2020; 2020. DOI: 10.1155/2020/5139237.
47. Cao R, Chen X, Xing F, et al. Cross-sectional and longitudinal associations between probable rapid eye movement sleep behavior disorder and impulse control disorders in Parkinson’s disease. *European Journal of Neurology* 2020; 27: 757-763. DOI: 10.1111/ene.14177.

48. Carbunaru S, Eisinger RS, Ramirez-Zamora A, et al. Impulse control disorders in Parkinson's: Sleep disorders and nondopaminergic associations. *Brain and Behavior* 2018; 8. DOI: 10.1002/brb3.904.
49. Castrioto A, Funkiewiez A, Debû B, et al. Iowa gambling task impairment in Parkinson's disease can be normalised by reduction of dopaminergic medication after subthalamic stimulation. *Journal of Neurology, Neurosurgery and Psychiatry* 2015; 86: 186-190. DOI: 10.1136/jnnp-2013-307146.
50. Catalán MJ, de Pablo-Fernández E, Villanueva C, et al. Levodopa infusion improves impulsivity and dopamine dysregulation syndrome in Parkinson's disease. *Movement Disorders* 2013; 28: 2007-2010. DOI: 10.1002/mds.25636.
51. Catalan MJ, Molina-Arjona JA, Mir P, et al. Improvement of impulse control disorders associated with levodopa–carbidopa intestinal gel treatment in advanced Parkinson’s disease. *Journal of Neurology* 2018; 265: 1279-1287. DOI: 10.1007/s00415-018-8803-1.
52. Celik E, Ozkaya HM, Poyraz BC, et al. Impulse control disorders in patients with prolactinoma receiving dopamine agonist therapy: a prospective study with 1 year follow-up. *Endocrine* 2018; 62: 692-700. DOI: 10.1007/s12020-018-1744-8.
53. Cera N, Bifolchetti S, Martinotti G, et al. Amantadine and cognitive flexibility: Decision making in Parkinson's patients with severe pathological gambling and other impulse control disorders. *Neuropsychiatric Disease and Treatment* 2014; 10: 1093-1101. DOI: 10.2147/NDT.S54423.
54. Chaudhuri KR, Todorova A, Nirenberg MJ, et al. A Pilot Prospective, Multicenter Observational Study of Dopamine Agonist Withdrawal Syndrome in Parkinson's Disease. *Movement Disorders Clinical Practice* 2015; 2: 170-174. DOI: 10.1002/mdc3.12141.
55. Chen L, He X, Tao T, et al. Association between metabolic syndrome components and impulse control disorders in Parkinson’s disease. *Frontiers in Neuroscience* 2023; 17. DOI: 10.3389/fnins.2023.1191338.
56. Chen L, He X, Mao L, et al. APOE contributes to longitudinal impulse control disorders progression in Parkinson’s disease. *BMC Psychiatry* 2024; 24. DOI: 10.1186/s12888-024-06084-5.
57. Chiang HL, Huang YS, Chen ST, et al. Are there ethnic differences in impulsive/compulsive behaviors in Parkinson's disease? *European Journal of Neurology* 2012; 19: 494-500. DOI: 10.1111/j.1468-1331.2011.03571.x.
58. Choi JH, Lee JY, Jeon B, et al. Neuropsychiatric Traits Associated with Refractory Impulse Control Disorder in Parkinson's Disease. *Neurodegenerative Diseases* 2020; 19: 171-177. DOI: 10.1159/000507447.
59. Cilia R, Siri C, Canesi M, et al. Dopamine dysregulation syndrome in Parkinson's disease: From clinical and neuropsychological characterisation to management and long-term outcome. *Journal of Neurology, Neurosurgery and Psychiatry* 2014; 85: 311-318. DOI: 10.1136/jnnp-2012-303988.
60. Contin M, Lopane G, Marini L, et al. Screening for impulse control disorders in Parkinson’s disease and dopamine agonist use: a study of pharmacokinetic and psychological risk factors. *Neurological Sciences* 2023; 44: 565-572. DOI: 10.1007/s10072-022-06485-y.
61. Cooper CA, Jadidian A, Paggi M, et al. Prevalence of hypersexual behavior in Parkinson's disease patients: Not restricted to males and dopamine agonist use. *International Journal of General Medicine* 2009; 2: 57-61. DOI: 10.2147/ijgm.s4674.
62. Corbeil O, Corbeil S, Dorval M, et al. Problem Gambling Associated with Aripiprazole: A Nested Case-Control Study in a First-Episode Psychosis Program. *CNS Drugs* 2021; 35: 461-468. DOI: 10.1007/s40263-021-00801-4.
63. Corvol J-C, Artaud F, Cormier-Dequaire F, et al. Longitudinal analysis of impulse control disorders in Parkinson disease. *Neurology* 2018; 91: e189-e201. DOI: doi:10.1212/WNL.0000000000005816.
64. Crockford D, Quickfall J, Currie S, et al. Prevalence of problem and pathological gambling in Parkinson's disease. *J Gambl Stud* 2008; 24: 411-422. 20080617. DOI: 10.1007/s10899-008-9099-3.
65. Cuciureanu DI, Croitoru CG, Constantinescu V, et al. Neuropsychiatric changes in parkinson’s disease patients: A prospective observational two years study. *Romanian Journal of Neurology/ Revista Romana de Neurologie* 2019; 18: 161-166.
66. Cunnington A-L, White L and Hood K. Identification of possible risk factors for the development of dopamine agonist withdrawal syndrome in Parkinson's disease. *Parkinsonism & related disorders* 2012; 18: 1051-1052. DOI: 10.1016/j.parkreldis.2012.05.012.
67. Dalrymple WA, Trach SK, Flanigan JL, et al. Psychiatric predictors of quality of life in Parkinson's disease: A three-year longitudinal study. *Journal of the Neurological Sciences* 2024; 466. DOI: 10.1016/j.jns.2024.123248.
68. Dang D, Cunnington D and Swieca J. The Emergence of Devastating Impulse Control Disorders During Dopamine Agonist Therapy of the Restless Legs Syndrome. *Clinical Neuropharmacology* 2011; 34.

69. Dawood S, Smith JG, Nadeem N, et al. Safety and effectiveness of long-acting injectable Aripiprazole in daily practice: A naturalistic retrospective study. *European Journal of Psychiatry* 2020; 34: 82-89. DOI: 10.1016/j.ejpsy.2020.02.001.
70. De Chazeron I, Llorca PM, Chéreau-Boudet I, et al. Hypersexuality and pathological gambling in Parkinson's disease: A cross-sectional case-control study. *Movement Disorders* 2011; 26: 2127-2130. DOI: 10.1002/mds.23845.
71. de Chazeron I, Durif F, Chereau-Boudet I, et al. Compulsive eating behaviors in Parkinson's disease. *Eating and Weight Disorders* 2019; 24: 421-429. DOI: 10.1007/s40519-019-00648-1.
72. Delaney M, Simpson J and Leroi I. Perceptions of cause and control of impulse control behaviours in people with Parkinson's disease. *British journal of health psychology* 2012; 17: 522-535.
73. de la Riva P, Smith K, Xie SX, et al. Course of psychiatric symptoms and global cognition in early Parkinson disease. *Neurology* 2014; 83: 1096-1103. DOI: 10.1212/WNL.0000000000000801.
74. Delpont B, Lhommée E, Klinger H, et al. Psychostimulant effect of dopaminergic treatment and addictions in Parkinson's disease. *Mov Disord* 2017; 32: 1566-1573. DOI: 10.1002/mds.27101.
75. De Sousa SMC, Baranoff J, Rushworth RL, et al. Impulse Control Disorders in Dopamine Agonist-Treated Hyperprolactinemia: Prevalence and Risk Factors. *Journal of Clinical Endocrinology and Metabolism* 2020; 105. DOI: 10.1210/clinem/dgz076.
76. De Wit LE, Wilting I, Souverein PC, et al. Impulse control disorders associated with dopaminergic drugs: A disproportionality analysis using vigibase. *European Neuropsychopharmacology* 2022; 58: 30-38. DOI: 10.1016/j.euroneuro.2022.01.113.
77. Diago EB, del Val LJL, Lasaosa SS, et al. Association between REM sleep behaviour disorder and impulse control disorder in Parkinson's disease; patients with Parkinson's disease. *Neurologia* 2017; 32: 494-499. DOI: 10.1016/j.nr1.2016.02.008.
78. Di Rosa E, Mapelli D, Ronconi L, et al. Anxiety predicts impulsive-compulsive behaviours in Parkinson's disease: Clinical relevance and theoretical implications. *Journal of Psychiatric Research* 2022; 148: 220-229. DOI: 10.1016/j.jpsychires.2022.01.052.
79. Dogansen SC, Cikrikcili U, Oruk G, et al. Dopamine Agonist-Induced Impulse Control Disorders in Patients with Prolactinoma: A Cross-Sectional Multicenter Study. *Journal of Clinical Endocrinology and Metabolism* 2019; 104: 2527-2534. DOI: 10.1210/jc.2018-02202.
80. Drew DS, Muhammed K, Baig F, et al. Dopamine and reward hypersensitivity in Parkinson's disease with impulse control disorder. *Brain* 2020; 143: 2502-2518. DOI: 10.1093/brain/awaa198.
81. Driver-Dunckley ED, Noble BN, Hentz JG, et al. Gambling and increased sexual desire with dopaminergic medications in restless legs syndrome. *Clinical Neuropharmacology* 2007; 30: 249-255. DOI: 10.1097/wnf.0b013e31804c780e.
82. Du S, Huang Y, Ma Y, et al. The mediating effects of depression, anxiety, and rapid eye movement sleep behavior disorder on the association between dopaminergic replacement therapy and impulse control disorders in Parkinson's disease. *Neurological Sciences* 2023; 44: 557-564. DOI: 10.1007/s10072-022-06443-8.
83. Dubaz OM, Wu S, Cubillos F, et al. Changes in Prescribing Practices of Dopaminergic Medications in Individuals with Parkinson's Disease by Expert Care Centers from 2010 to 2017: The Parkinson's Foundation Quality Improvement Initiative. *Movement Disorders Clinical Practice* 2019; 6: 687-692. DOI: 10.1002/mdc3.12837.
84. El Otmani H, Mouni FZ, Abdulhakeem Z, et al. Impulse control disorders in Parkinson disease: A cross-sectional study in Morocco. *Rev Neurol (Paris)* 2019; 175: 233-237. DOI: 10.1016/j.neurol.2018.07.009.
85. Erdal Y, Perk S, Alnak A, et al. Obsessive–compulsive disorder in restless legs syndrome. *Sleep and Biological Rhythms* 2021; 19: 393-398. DOI: 10.1007/s41105-021-00329-6.
86. Erga AH, Alves G, Larsen JP, et al. Impulsive and Compulsive Behaviors in Parkinson's Disease: The Norwegian ParkWest Study. *Journal of Parkinson's Disease* 2017; 7: 183-191. DOI: 10.3233/JPD-160977.
87. Erga AH, Alves G, Tysnes OB, et al. Evolution of impulsive–compulsive behaviors and cognition in Parkinson's disease. *Journal of Neurology* 2020; 267: 259-266. DOI: 10.1007/s00415-019-09584-7.
88. Erga AH, Alves G, Tysnes OB, et al. Impulsive and compulsive behaviors in Parkinson's disease: Impact on quality of and satisfaction with life, and caregiver burden. *Parkinsonism and Related Disorders* 2020; 78: 27-30. DOI: 10.1016/j.parkreldis.2020.07.007.
89. Etminan M, Sodhi M, Samii A, et al. Risk of Gambling Disorder and Impulse Control Disorder with Aripiprazole, Pramipexole, and Ropinirole: A Pharmacoepidemiologic Study. *Journal of Clinical Psychopharmacology* 2017; 37: 102-104. DOI: 10.1097/JCP.0000000000000634.

90. Eusebio A, Witjas T, Cohen J, et al. Subthalamic nucleus stimulation and compulsive use of dopaminergic medication in Parkinson's disease. *Journal of Neurology, Neurosurgery and Psychiatry* 2013; 84: 868-874. DOI: 10.1136/jnnp-2012-302387.
91. Evans AH, Katzenschlager R, Paviour D, et al. Punding in Parkinson's disease: Its relation to the dopamine dysregulation syndrome. *Movement Disorders* 2004; 19: 397-405. DOI: 10.1002/mds.20045.
92. Evans AH, Lawrence AD, Potts J, et al. Factors influencing susceptibility to compulsive dopaminergic drug use in Parkinson disease. *Neurology* 2005; 65: 1570-1574. DOI: 10.1212/01.wnl.0000184487.72289.f0.
93. Evans AH, Lawrence AD, Cresswell SA, et al. Compulsive use of dopaminergic drug therapy in Parkinson's disease: Reward and anti-reward. *Movement Disorders* 2010; 25: 867-876. DOI: 10.1002/mds.22898.
94. Evans AH, Kettlewell J, McGregor S, et al. A conditioned response as a measure of impulsive-compulsive behaviours in Parkinson's disease. *PLoS One* 2014; 9. DOI: 10.1371/journal.pone.0089319.
95. Fabbri M, Rousseau V, Corvol JC, et al. Amantadine use in the French prospective NS-Park cohort. *Journal of Neural Transmission* 2024; 131: 799-811. DOI: 10.1007/s00702-024-02772-4.
96. Fan W, Ding H, Ma J, et al. Impulse control disorders in Parkinson's disease in a Chinese population. *Neuroscience Letters* 2009; 465: 6-9. DOI: 10.1016/j.neulet.2009.06.074.
97. Fantini ML, Macedo L, Zibetti M, et al. Increased risk of impulse control symptoms in Parkinson's disease with REM sleep behaviour disorder. *Journal of Neurology, Neurosurgery and Psychiatry* 2015; 86: 174-179. DOI: 10.1136/jnnp-2014-307904.
98. Fantini ML, Figorilli M, Arnulf I, et al. Sleep and REM sleep behaviour disorder in Parkinson's disease with impulse control disorder. *Journal of Neurology, Neurosurgery and Psychiatry* 2018; 89: 305-310. DOI: 10.1136/jnnp-2017-316576.
99. Fantini ML, Fedler J, Pereira B, et al. Is Rapid Eye Movement Sleep Behavior Disorder a Risk Factor for Impulse Control Disorder in Parkinson Disease? *Annals of Neurology* 2020; 88: 759-770. DOI: 10.1002/ana.25798.
100. Faouzi J, Bekadar S, Artaud F, et al. Machine Learning-Based Prediction of Impulse Control Disorders in Parkinson's Disease From Clinical and Genetic Data. *IEEE Open J Eng Med Biol* 2022; 3: 96-107. DOI: 10.1109/ojemb.2022.3178295.
101. Farnikova K, Obereigneru R, Kanovsky P, et al. Comparison of personality characteristics in parkinson disease patients with and without impulse control disorders and in healthy volunteers. *Cognitive and Behavioral Neurology* 2012; 25: 25-33. DOI: 10.1097/WNN.0b013e31824b4103.
102. Fasano A, Ricciardi L, Pettorruso M, et al. Management of punding in Parkinson's disease: An open-label prospective study. *Journal of Neurology* 2011; 258: 656-660. DOI: 10.1007/s00415-010-5817-8.
103. Fusaroli M, Raschi E, Giunchi V, et al. Impulse Control Disorders by Dopamine Partial Agonists: A Pharmacovigilance-Pharmacodynamic Assessment through the FDA Adverse Event Reporting System. *International Journal of Neuropsychopharmacology* 2022; 25: 727-736. DOI: 10.1093/ijnp/pyac031.
104. Fusaroli M, Giunchi V, Battini V, et al. Exploring the underlying mechanisms of drug-induced impulse control disorders: a pharmacovigilance-pharmacodynamic study. *Psychiatry and Clinical Neurosciences* 2023; 77: 160-167. DOI: 10.1111/pcn.13511.
105. Fusaroli M, Polizzi S, Menestrina L, et al. Unveiling the Burden of Drug-Induced Impulsivity: A Network Analysis of the FDA Adverse Event Reporting System. *Drug Safety* 2024; 47: 1275-1292. DOI: 10.1007/s40264-024-01471-z.
106. Galbiati A, Marelli S, Giora E, et al. Neurocognitive function in patients with idiopathic Restless Legs Syndrome before and after treatment with dopamine-agonist. *International Journal of Psychophysiology* 2015; 95: 304-309. DOI: 10.1016/j.ijpsycho.2014.12.005.
107. García-Rubio MI, Otero-Cerdeira ME, Toledo-Lozano CG, et al. Analysis of Impulse Control Disorders (ICDs) and Factors Associated with Their Development in a Parkinson's Disease Population. *Healthcare (Basel)* 2021; 9. DOI: 10.3390/healthcare9101263.
108. Garcia-Ruiz PJ, Martinez Castrillo JC, Alonso-Canovas A, et al. Impulse control disorder in patients with Parkinson's disease under dopamine agonist therapy: A multicentre study. *Journal of Neurology, Neurosurgery and Psychiatry* 2014; 85: 841-845. DOI: 10.1136/jnnp-2013-306787.
109. Garrett AR, Bazaco SL, Clausen SS, et al. Epidemiology of impulse control disorders and association with dopamine agonist exposure, active component, U.S. Armed Forces, 2014-2018. *Msmr* 2019; 26: 10-16.
110. Gee L, Smith H, De La Cruz P, et al. The Influence of Bilateral Subthalamic Nucleus Deep Brain Stimulation on Impulsivity and Prepulse Inhibition in Parkinson's Disease Patients. *Stereotactic and Functional Neurosurgery* 2015; 93: 265-270. DOI: 10.1159/000381558.

111. Gendreau KE and Potenza MN. Detecting associations between behavioral addictions and dopamine agonists in the Food & Drug Administration's Adverse Event database. *Journal of Behavioral Addictions* 2014; 3: 21-26. DOI: 10.1556/JBA.3.2014.1.3.
112. Gescheidt T, Losada VILY, Menšíková K, et al. Impulse control disorders in patients with young-onset Parkinson's disease: A cross-sectional study seeking associated factors. *Basal Ganglia* 2016; 6: 197-205. DOI: 10.1016/j.baga.2016.09.001.
113. Giladi N, Weitzman N, Schreiber S, et al. New onset heightened interest or drive for gambling, shopping, eating or sexual activity in patients with Parkinson's disease: The role of dopamine agonist treatment and age at motor symptoms onset. *Journal of Psychopharmacology* 2007; 21: 501-506. DOI: 10.1177/0269881106073109.
114. Giladi N, Boroojerdi B and Surmann E. The safety and tolerability of rotigotine transdermal system over a 6-year period in patients with early-stage Parkinson's disease. *Journal of Neural Transmission* 2013; 120: 1321-1329. DOI: 10.1007/s00702-013-1001-5.
115. Goerlich-Dobre KS, Probst C, Winter L, et al. Alexithymia-an independent risk factor for impulsive-compulsive disorders in Parkinson's disease. *Movement Disorders* 2014; 29: 214-220. DOI: 10.1002/mds.25679.
116. Gómez CCR, Dueñas MS, Bernal O, et al. A multi center comparative study of impulse control disorder in Latin American patients with Parkinson disease. *Clinical Neuropharmacology* 2017; 40: 51-55. DOI: 10.1097/WNF.0000000000000202.
117. Grall-Bronnec M, Victorri-Vigneau C, Rouaud T, et al. Parkinson's disease and iatrogenic impulsive- compulsive behaviors: A case/non-case study to build a complete model of individual vulnerability. *Journal of Behavioral Addictions* 2022; 11: 766-777. DOI: 10.1556/2006.2022.00051.
118. Grall-Bronnec M, Verholleman A, Victorri-Vigneau C, et al. Parkinson's Disease, Impulsive-Compulsive Behaviors, and Health-Related Quality of Life. *J Mov Disord* 2024; 17: 82-88. DOI: 10.14802/jmd.23042.
119. Grosset KA, Macphee G, Pal G, et al. Problematic gambling on dopamine agonists: Not such a rarity. *Movement Disorders* 2006; 21: 2206-2208. DOI: 10.1002/mds.21110.
120. Gunduz A, Çiftçi T, Erbil AC, et al. Impulse control disorders in Parkinson's disease: a retrospective analysis of 1824 patients in a 12-year period. *Neurological Sciences* 2024; 45: 171-175. DOI: 10.1007/s10072-023-07006-1.
121. Gungabissoon U, Kirichek O, El Baou C, et al. Comparison of long-term use of prolonged-release ropinirole and immediate-release dopamine agonists in an observational study in patients with Parkinson's disease. *Pharmacoepidemiology and Drug Safety* 2020; 29: 591-598. DOI: 10.1002/pds.4986.
122. Hack N, Akbar U, Thompson-Avila A, et al. Impulsive and compulsive behaviors in Parkinson Study Group (PSG) centers performing deep brain stimulation surgery. *Journal of Parkinson's Disease* 2014; 4: 591-598. DOI: 10.3233/JPD-140357.
123. Hall A, Weightman M, Jenkinson N, et al. Performance on the balloon analogue risk task and anticipatory response inhibition task is associated with severity of impulse control behaviours in people with Parkinson's disease. *Experimental Brain Research* 2023; 241: 1159-1172. DOI: 10.1007/s00221-023-06584-y.
124. Hassan A, Bower JH, Kumar N, et al. Dopamine agonist-triggered pathological behaviors: Surveillance in the PD clinic reveals high frequencies. *Parkinsonism and Related Disorders* 2011; 17: 260-264. DOI: 10.1016/j.parkreldis.2011.01.009.
125. Hauser RA, Schapira AHV, Barone P, et al. Long-term safety and sustained efficacy of extended-release pramipexole in early and advanced Parkinson's disease. *European Journal of Neurology* 2014; 21: 736-743. DOI: 10.1111/ene.12375.
126. Heim B, Djamshidian A, Heidbreder A, et al. Augmentation and impulsive behaviors in restless legs syndrome: Coexistence or association? *Neurology* 2016; 87: 36-40. DOI: 10.1212/wnl.0000000000002803.
127. Heim B, Pertl MT, Stefani A, et al. Reflection impulsivity perceptual decision-making in patients with restless legs syndrome. *Annals of Clinical and Translational Neurology* 2018; 5: 315-322. DOI: 10.1002/acn3.535.
128. Heim B, Ellmerer P, Stefani A, et al. Birds of a feather flock together: Disadvantageous decision making in augmented restless legs syndrome patients with and without impulse control disorders. *Brain Sciences* 2021; 11. DOI: 10.3390/brainsci11030383.
129. Herath HMMTB, Wijayawardhana KWSM, Wickramarachchi UI, et al. Impulse control disorders and other nonmotor symptoms in Sri Lankan patients with Parkinson's disease. *PLoS One* 2024; 19. DOI: 10.1371/journal.pone.0312342.
130. Hernandez-Con P, Lin I, Mamikonyan E, et al. Course of Impulse Control Disorder Symptoms in Parkinson's Disease: Deep Brain Stimulation Versus Medications. *Movement Disorders Clinical Practice* 2023; 10: 903-913. DOI: 10.1002/mdc3.13738.
131. Hidding U, Gulberti A, Horn A, et al. Impact of Combined Subthalamic Nucleus and Substantia Nigra Stimulation on Neuropsychiatric Symptoms in Parkinson's Disease Patients. *Parkinson's Disease* 2017; 2017. DOI: 10.1155/2017/7306192.

132. Hinkle JT, Perepezko K, Mills KA, et al. Attentional dysfunction and the punding spectrum in Parkinson's disease. *Parkinsonism and Related Disorders* 2021; 84: 23-28. DOI: 10.1016/j.parkreldis.2021.01.019.
133. Hinojosa-Amaya JM, Johnson N, González-Torres C, et al. Depression and Impulsivity Self-Assessment Tools to Identify Dopamine Agonist Side Effects in Patients With Pituitary Adenomas. *Frontiers in Endocrinology* 2020; 11. DOI: 10.3389/fendo.2020.579606.
134. Hollingworth SA, McGuire TM, Pache D, et al. Dopamine Agonists: Time Pattern of Adverse Effects Reporting in Australia. *Drugs - Real World Outcomes* 2015; 2: 199-203. DOI: 10.1007/s40801-015-0028-3.
135. Holman AJ. Impulse control disorder behaviors associated with pramipexole used to treat fibromyalgia. *Journal of Gambling Studies* 2009; 25: 425-431. DOI: 10.1007/s10899-009-9123-2.
136. Hu L, Lin C, Lin F, et al. Different impulse control disorder evolution patterns and white matter microstructural damage in the progression of Parkinson's disease. *Frontiers in Aging Neuroscience* 2023; 15. DOI: 10.3389/fnagi.2023.1260630.
137. Hurt CS, Alkufri F, Brown RG, et al. Motor phenotypes, medication and mood: Further associations with impulsive behaviours in Parkinson's disease. *Journal of Parkinson's Disease* 2014; 4: 245-254. DOI: 10.3233/JPD-130314.
138. Hälbig TD, Tse W, Frisina PG, et al. Subthalamic deep brain stimulation and impulse control in Parkinson's disease. *European Journal of Neurology* 2009; 16: 493-497. DOI: 10.1111/j.1468-1331.2008.02509.x.
139. Ihle J, Artaud F, Bekadar S, et al. Parkinson's disease polygenic risk score is not associated with impulse control disorders: A longitudinal study. *Parkinsonism and Related Disorders* 2020; 75: 30-33. DOI: 10.1016/j.parkreldis.2020.03.017.
140. Imamura A, Geda YE, Slowinski J, et al. Medications used to treat Parkinson's disease and the risk of gambling. *European Journal of Neurology* 2008; 15: 350-354. DOI: 10.1111/j.1468-1331.2008.02081.x.
141. Inoue Y, Kuroda K, Hirata K, et al. Efficacy, safety and dose-response of pramipexole in Japanese patients with primary restless legs syndrome: Randomized trial. *Neuropsychobiology* 2010; 63: 35-42. DOI: 10.1159/000322289.
142. Irmen F, Horn A, Meder D, et al. Sensorimotor subthalamic stimulation restores risk-reward trade-off in Parkinson's disease. *Movement Disorders* 2019; 34: 366-376. DOI: 10.1002/mds.27576.
143. Isaias IU, Siri C, Cilia R, et al. The relationship between impulsivity and impulse control disorders in Parkinson's disease. *Movement Disorders* 2008; 23: 411-415. DOI: 10.1002/mds.21872.
144. Izzo VA, Donati MA, Torre E, et al. Impulse control disorders in Parkinson's disease versus in healthy controls: A different predictive model. *Journal of neuropsychology* 2020; 14: 318-332. DOI: 10.1111/jnp.12193.
145. Jaakkola E, Kaasinen V, Siri C, et al. Impulse control disorders are associated with multiple psychiatric symptoms in Parkinson's disease. *Journal of Parkinson's Disease* 2014; 4: 507-515. DOI: 10.3233/JPD-140351.
146. Jakobsen KD, Bruhn CH, Pagsberg AK, et al. Neurological, metabolic, and psychiatric adverse events in children and adolescents treated with aripiprazole. *Journal of Clinical Psychopharmacology* 2016; 36: 496-499. DOI: 10.1097/JCP.0000000000000548.
147. Janssen MLF, Duits AA, Tourai AM, et al. Subthalamic nucleus high-frequency stimulation for advanced Parkinson's disease: Motor and neuropsychological outcome after 10 years. *Stereotactic and Functional Neurosurgery* 2014; 92: 381-387. DOI: 10.1159/000366066.
148. Jeon N and Bortolato M. What drugs modify the risk of iatrogenic impulse-control disorders in Parkinson's disease? A preliminary pharmacoepidemiologic study. *PLoS One* 2020; 15. DOI: 10.1371/journal.pone.0227128.
149. Jesús S, Labrador-Espinosa MA, Adarmes AD, et al. Non-motor symptom burden in patients with Parkinson's disease with impulse control disorders and compulsive behaviours: results from the COPPADIS cohort. *Scientific reports* 2020; 10: 16893. DOI: 10.1038/s41598-020-73756-z.
150. Jesús S, Periñán MT, Cortés C, et al. Integrating genetic and clinical data to predict impulse control disorders in Parkinson's disease. *European Journal of Neurology* 2021; 28: 459-468. DOI: 10.1111/ene.14590.
151. Jeyadevan A, Bakeberg M, Byrnes M, et al. Quality of life implications for elevated trait impulsivity in people with Parkinson's disease. *Quality of Life Research* 2023; 32: 1143-1150. DOI: 10.1007/s11136-022-03321-w.
152. Jiménez-Murcia S, Bove FI, Israel M, et al. Cognitive-Behavioral Therapy for Pathological Gambling in Parkinson's Disease: A Pilot Controlled Study. *European Addiction Research* 2012; 18: 265-274. DOI: 10.1159/000337442.

153. Johnson D, Townsend L, David AS, et al. Predictors of Burden in Carers of Patients with Impulse Control Behaviors in Parkinson's Disease. *Movement Disorders Clinical Practice* 2023; 10: 1360-1367. DOI: 10.1002/mdc3.13824.
154. Joutsa J, Martikainen K, Vahlberg T, et al. Effects of dopamine agonist dose and gender on the prognosis of impulse control disorders in Parkinson's disease. *Parkinsonism and Related Disorders* 2012; 18: 1079-1083. DOI: 10.1016/j.parkreldis.2012.06.005.
155. Joutsa J, Martikainen K, Vahlberg T, et al. Impulse control disorders and depression in Finnish patients with Parkinson's disease. *Parkinsonism and Related Disorders* 2012; 18: 155-160. DOI: 10.1016/j.parkreldis.2011.09.007.
156. Kapsomenakis A, Kasselimis D, Vaniotis E, et al. Frequency of Impulsive-Compulsive Behavior and Associated Psychological Factors in Parkinson's Disease: Lack of Control or Too Much of It? *Medicina (Kaunas, Lithuania)* 2023; 59. DOI: 10.3390/medicina59111942.
157. Ke XA, Wang LJ, Chen MP, et al. The side effects of dopamine receptor agonist drugs in Chinese prolactinoma patients: a cross sectional study. *BMC ENDOCRINE DISORDERS* 2022; 22. DOI: 10.1186/s12902-022-01009-3.
158. Kehagia AA, Housden CR, Regenthal R, et al. Targeting impulsivity in Parkinson's disease using atomoxetine. *Brain* 2014; 137: 1986-1997. DOI: 10.1093/brain/awu117.
159. Kenangil G, Özekmekçi S, Sohtaoglu M, et al. Compulsive behaviors in patients with Parkinson's disease. *Neurologist* 2010; 16: 192-195. DOI: 10.1097/NRL.0b013e31819f952b.
160. Kim J, Kim M, Kwon DY, et al. Clinical characteristics of impulse control and repetitive behavior disorders in Parkinson's disease. *Journal of Neurology* 2013; 260: 429-437. DOI: 10.1007/s00415-012-6645-9.
161. Kim YE, Kim HJ, Lee JY, et al. Impulse control and related behaviors after bilateral subthalamic stimulation in patients with Parkinson's disease. *Journal of Clinical Neuroscience* 2013; 20: 964-969. DOI: 10.1016/j.jocn.2012.07.020.
162. Kim YE, Jeon BS, Yang HJ, et al. REM sleep behavior disorder: Association with motor complications and impulse control disorders in Parkinson's disease. *Parkinsonism and Related Disorders* 2014; 20: 1081-1084. DOI: 10.1016/j.parkreldis.2014.03.022.
163. Kim A, Kim YE, Kim HJ, et al. A 7-year observation of the effect of subthalamic deep brain stimulation on impulse control disorder in patients with Parkinson's disease. *Parkinsonism and Related Disorders* 2018; 56: 3-8. DOI: 10.1016/j.parkreldis.2018.07.010.
164. Kobylecki C, Carrasco R, Boca M, et al. The association between pain and impulse control behaviours in Parkinson's disease. *Parkinsonism and Related Disorders* 2020; 78: 53-55. DOI: 10.1016/j.parkreldis.2020.07.005.
165. Koh J, Takahashi M, Ohmae Y, et al. A single-arm open-label pilot study of brief mindfulness meditation to control impulsivity in Parkinson’s disease. *PLoS One* 2022; 17. DOI: 10.1371/journal.pone.0266354.
166. Kon T, Ueno T, Haga R, et al. The factors associated with impulse control behaviors in Parkinson's disease: A 2-year longitudinal retrospective cohort study. *Brain and Behavior* 2018; 8. DOI: 10.1002/brb3.1036.
167. Kovács M, Makkos A, Pintér D, et al. Screening for problematic Internet use may help identify impulse control disorders in Parkinson's disease. *Behavioural Neurology* 2019; 2019. DOI: 10.1155/2019/4925015.
168. Kraemmer J, Smith K, Weintraub D, et al. Clinical-genetic model predicts incident impulse control disorders in Parkinson's disease. *Journal of Neurology, Neurosurgery and Psychiatry* 2016. DOI: 10.1136/jnnp-2015-312848.
169. Krause P, Reimer J, Kaplan J, et al. Deep brain stimulation in Early Onset Parkinson's disease. *Frontiers in Neurology* 2022; 13. DOI: 10.3389/fneur.2022.1041449.
170. Krause P, Berking S, Astalosch M, et al. Motor and non-motor improvements following short-term multidisciplinary day-clinic care in Parkinson´s disease. *Journal of Neural Transmission* 2022; 129: 1419-1426. DOI: 10.1007/s00702-022-02562-w.
171. Kuhlman GD, Flanigan JL, Sperling SA, et al. Predictors of health-related quality of life in Parkinson's disease. *Parkinsonism and Related Disorders* 2019; 65: 86-90. DOI: 10.1016/j.parkreldis.2019.05.009.
172. Kuzu M, Özer İŞ, Herdi O, et al. Switch from immediate-release pramipexole to extended-release pramipexole: The safety and efficacy characteristics of sixty-eight patients. *Turk Noroloji Dergisi* 2016; 22: 114-116. DOI: 10.4274/tnd.48751.
173. Lamberti VMJ, Pereira B, Lhommée E, et al. Profile of Neuropsychiatric Symptoms in Parkinson's Disease: Surgical Candidates Compared to Controls. *Journal of Parkinson's Disease* 2016; 6: 133-142. DOI: 10.3233/JPD-150698.

174. Laurencin C, Timestit N, Marques A, et al. Efficacy and safety of clonidine for the treatment of impulse control disorder in Parkinson's disease: a multicenter, parallel, randomised, double-blind, Phase 2b Clinical trial. *Journal of Neurology* 2023; 270: 4851-4859. DOI: 10.1007/s00415-023-11814-y.
175. Lawrence AJ, Blackwell AD, Barker RA, et al. Predictors of punding in Parkinson's disease: Results from a questionnaire survey. *Movement Disorders* 2007; 22: 2339-2345. DOI: 10.1002/mds.21702.
176. Lee JY, Kim JM, Kim JW, et al. Association between the dose of dopaminergic medication and the behavioral disturbances in Parkinson disease. *Parkinsonism and Related Disorders* 2010; 16: 202-207. DOI: 10.1016/j.parkreldis.2009.12.002.
177. Lee JY, Jeon B, Koh SB, et al. Behavioural and trait changes in parkinsonian patients with impulse control disorder after switching from dopamine agonist to levodopa therapy: Results of REIN-PD trial. *Journal of Neurology, Neurosurgery and Psychiatry* 2019; 90: 30-37. DOI: 10.1136/jnnp-2018-318942.
178. Leplow B, Renftle D, Thomas M, et al. Characteristics of behavioural addiction in Parkinson's disease patients with self-reported impulse control disorder and controls matched for levodopa equivalent dose: A matched case–control study. *Journal of Neural Transmission* 2023; 130: 125-133. DOI: 10.1007/s00702-023-02588-8.
179. Leroi I, Ahearn DJ, Andrews M, et al. Behavioural disorders, disability and quality of life in Parkinson's disease. *Age and Ageing* 2011; 40: 614-621. DOI: 10.1093/ageing/afr078.
180. Leroi I, Harbishettar V, Andrews M, et al. Carer burden in apathy and impulse control disorders in Parkinson's disease. *International Journal of Geriatric Psychiatry* 2012; 27: 160-166. DOI: 10.1002/gps.2704.
181. Leroi I, Andrews M, McDonald K, et al. Apathy and impulse control disorders in Parkinson's disease: A direct comparison. *Parkinsonism and Related Disorders* 2012; 18: 198-203. DOI: 10.1016/j.parkreldis.2011.10.005.
182. Leroi I, Barraclough M, McKie S, et al. Dopaminergic influences on executive function and impulsive behaviour in impulse control disorders in Parkinson's disease. *J Neuropsychol* 2013; 7: 306-325. DOI: 10.1111/jnp.12026.
183. Lertxundi U, Hernandez R, Medrano J, et al. Aripiprazole and impulse control disorders: Higher risk with the intramuscular depot formulation? *International Clinical Psychopharmacology* 2018; 33: 56-58. DOI: 10.1097/YIC.0000000000000194.
184. Lhommée E, Klinger H, Thobois S, et al. Subthalamic stimulation in Parkinson's disease: Restoring the balance of motivated behaviours. *Brain* 2012; 135: 1463-1477. DOI: 10.1093/brain/aws078.
185. Liang K, Li X, Ma J, et al. Predictors of dopamine dysregulation syndrome in patients with early Parkinson's disease. *Neurological Sciences* 2023; 44: 4333-4342. DOI: 10.1007/s10072-023-06956-w.
186. Lim SY, Tan ZK, Ngam PI, et al. Impulsive-compulsive behaviors are common in Asian Parkinson's disease patients: Assessment using the QUIP. *Parkinsonism and Related Disorders* 2011; 17: 761-764. DOI: 10.1016/j.parkreldis.2011.07.009.
187. Limotai N, Oyama G, Go C, et al. Addiction-like manifestations and Parkinson's disease: a large single center 9-year experience. *Int J Neurosci* 2012; 122: 145-153. DOI: 10.3109/00207454.2011.633722.
188. Lipford MC and Silber MH. Long-term use of pramipexole in the management of restless legs syndrome. *Sleep Medicine* 2012; 13: 1280-1285. DOI: 10.1016/j.sleep.2012.08.004.
189. Liu W, Yamamoto T, Yamanaka Y, et al. Neuropsychiatric Symptoms in Parkinson's Disease After Subthalamic Nucleus Deep Brain Stimulation. *Frontiers in Neurology* 2021; 12. DOI: 10.3389/fneur.2021.656041.
190. Lopiano L, Modugno N, Marano P, et al. Motor and non-motor outcomes in patients with advanced Parkinson's disease treated with levodopa/carbidopa intestinal gel: final results of the GREENFIELD observational study. *Journal of Neurology* 2019; 266: 2164-2176. DOI: 10.1007/s00415-019-09337-6.
191. Lubomski M, Davis RL and Sue CM. Health-Related Quality of Life for Parkinson's Disease Patients and Their Caregivers. *J Mov Disord* 2021; 14: 42-52. DOI: 10.14802/jmd.20079.
192. Lulé D, Heimrath J, Pinkhardt EH, et al. Deep brain stimulation and behavioural changes: Is comedication the most important factor? *Neurodegenerative Diseases* 2012; 9: 18-24. DOI: 10.1159/000328817.
193. Lyons KE, Friedman JH, Hermanowicz N, et al. Orally disintegrating selegiline in Parkinson patients with dopamine agonist-related adverse effects. *Clinical Neuropharmacology* 2010; 33: 5-10. DOI: 10.1097/WNF.0b013e3181b7926f.
194. Maggi G, Vitale C, Giacobbe C, et al. Validation of the Italian version of the Questionnaire for Impulsive-Compulsive Disorders in Parkinson's Disease-Rating Scale (QUIP-RS) in an Italian Parkinson's disease cohort. *Neurological Sciences* 2024; 45: 3153-3161. DOI: 10.1007/s10072-024-07304-2.

195. Maia AF, Pinto AS, Barbosa ER, et al. Obsessive-compulsive symptoms, obsessive-compulsive disorder, and related disorders in Parkinson's disease. *Journal of Neuropsychiatry and Clinical Neurosciences* 2003; 15: 371-374. DOI: 10.1176/appi.neuropsych.15.3.371.
196. Mamikonyan E, Siderowf AD, Duda JE, et al. Long-term follow-up of impulse control disorders in Parkinson's disease. *Movement Disorders* 2008; 23: 75-80. DOI: 10.1002/mds.21770.
197. Marder SR, West B, Lau GS, et al. Aripiprazole effects in patients with acute schizophrenia experiencing higher or lower agitation: A post hoc analysis of 4 randomized, placebo-controlled clinical trials. *Journal of Clinical Psychiatry* 2007; 68: 662-668. DOI: 10.4088/JCP.v68n0503.
198. Maréchal E, Denoiseux B, Thys E, et al. Impulsive-Compulsive Behaviours in Belgian-Flemish Parkinson's Disease Patients: A Questionnaire-Based Study. *Parkinson's Disease* 2019; 2019. DOI: 10.1155/2019/7832487.
199. Marín-Lahoz J, Pagonabarraga J, Martinez-Horta S, et al. Parkinson's Disease: Impulsivity Does Not Cause Impulse Control Disorders but Boosts Their Severity. *Frontiers in Psychiatry* 2018; 9. DOI: 10.3389/fpsyt.2018.00465.
200. Marín-Lahoz J, Sampedro F, Martinez-Horta S, et al. Depression as a Risk Factor for Impulse Control Disorders in Parkinson Disease. *Annals of Neurology* 2019; 86: 762-769. DOI: 10.1002/ana.25581.
201. Marín-Lahoz J, Martinez-Horta S, Pagonabarraga J, et al. Predicting Impulse Control Disorders in Parkinson Disease through Incentive Biomarkers. *Annals of Neurology* 2022; 92: 974-984. DOI: 10.1002/ana.26486.
202. Marković V, Stanković I, Petrović I, et al. Dynamics of impulsive–compulsive behaviors in early Parkinson’s disease: a prospective study. *Journal of Neurology* 2020; 267: 1127-1136. DOI: 10.1007/s00415-019-09692-4.
203. Marques A, Figorilli M, Pereira B, et al. Impulse control disorders in Parkinson's disease patients with RLS: a cross sectional-study. *Sleep Medicine* 2018; 48: 148-154. DOI: 10.1016/j.sleep.2018.02.004.
204. Marques A, Figorilli M, Lambert C, et al. The urge to move: From restless legs syndrome to impulse control disorders in Parkinson’s disease. *Journal of Sleep Research* 2021; 30. DOI: 10.1111/jsr.13127.
205. Marques A, Pereira B, Figorilli M, et al. Decision making under uncertainty in Parkinson's disease with Rem sleep behavior disorder. *Sleep Medicine* 2022; 90: 214-221. DOI: 10.1016/j.sleep.2022.01.025.
206. Martinez-Martin P, Wan YM, Ray Chaudhuri K, et al. Impulse control and related behaviors in Parkinson’s disease with dementia. *European Journal of Neurology* 2020; 27: 944-950. DOI: 10.1111/ene.14169.
207. Martinez-Nunez AE, Hutchinson H, Coutinho P, et al. Clinically probable RBD is an early predictor of malignant non-motor Parkinson’s disease phenotypes. *npj Parkinson's Disease* 2025; 11. DOI: 10.1038/s41531-025-00874-8.
208. Martini A, Ellis SJ, Grange JA, et al. Risky decision-making and affective features of impulse control disorders in Parkinson’s disease. *Journal of Neural Transmission* 2018; 125: 131-143. DOI: 10.1007/s00702-017-1807-7.
209. Martini A, Weis L, Fiorenzato E, et al. Impact of Cognitive Profile on Impulse Control Disorders Presence and Severity in Parkinson's Disease. *Frontiers in Neurology* 2019; 10. DOI: 10.3389/fneur.2019.00266.
210. Martinkova J, Trejbalova L, Sasikova M, et al. Impulse control disorders associated with dopaminergic medication in patients with pituitary adenomas. *Clinical Neuropharmacology* 2011; 34: 179-181. DOI: 10.1097/WNF.0b013e3182281b2f.
211. McKeown E, Saleem T, Magee C, et al. The experiences of carers looking after people with Parkinson's disease who exhibit impulsive and compulsive behaviours: An exploratory qualitative study. *Journal of clinical nursing* 2020; 29: 4623-4632. DOI: 10.1111/jocn.15499.
212. Meira B, Degos B, Corsetti E, et al. Long-term effect of apomorphine infusion in advanced Parkinson’s disease: a real-life study. *npj Parkinson's Disease* 2021; 7. DOI: 10.1038/s41531-021-00194-7.
213. Meng D, Jin Z, Wang Y, et al. Longitudinal cognitive changes in patients with early Parkinson's disease and neuropsychiatric symptoms. *CNS Neuroscience and Therapeutics* 2023; 29: 2259-2266. DOI: 10.1111/cns.14173.
214. Merola A, Romagnolo A, Rizzi L, et al. Impulse control behaviors and subthalamic deep brain stimulation in Parkinson disease. *Journal of Neurology* 2017; 264: 40-48. DOI: 10.1007/s00415-016-8314-x.
215. Miyasaki JM, Al Hassan K, Lang AE, et al. Punding prevalence in Parkinson's disease. *Movement Disorders* 2007; 22: 1179-1181. DOI: 10.1002/mds.21296.

216. Mohammad ME, Vizcarra JA, Garcia X, et al. Impact of behavioral side effects on the management of Parkinson patients treated with dopamine agonists. *Clinical Parkinsonism and Related Disorders* 2021; 4. DOI: 10.1016/j.prdoa.2021.100091.
217. Mohammad MM, Alidrisi HA and Mansour AA. Impulse Control Disorders in Southern Iraqi Patients Medicated With Cabergoline for Prolactinoma. *Cureus* 2024; 16: e58516. DOI: 10.7759/cureus.58516.
218. Monaco MRL, Petracca M, Weintraub D, et al. Prevalence of impulsive-compulsive symptoms in elderly Parkinson's disease patients: A case-control study. *Journal of Clinical Psychiatry* 2018; 79. DOI: 10.4088/JCP.17m11612.
219. Moore TJ, Glenmullen J and Mattison DR. Reports of pathological gambling, hypersexuality, and compulsive shopping associated with dopamine receptor agonist drugs. *JAMA Internal Medicine* 2014; 174: 1930-1933. DOI: 10.1001/jamainternmed.2014.5262.
220. Morgante F, Fasano A, Ginevrino M, et al. Impulsive-compulsive behaviors in parkin -associated Parkinson disease. *Neurology* 2016; 87: 1436-1441. DOI: 10.1212/WNL.0000000000003177.
221. Morgante F, Oppo V, Fabbri M, et al. Levodopa–carbidopa intrajejunal infusion in Parkinson’s disease: untangling the role of age. *Journal of Neurology* 2021; 268: 1728-1737. DOI: 10.1007/s00415-020-10356-x.
222. Morrow CB, Hinkle JT, Seemiller J, et al. Examining the link between impulse control disorder and antidepressant use in Parkinson's disease. *Parkinsonism and Related Disorders* 2023; 117. DOI: 10.1016/j.parkreldis.2023.105918.
223. Morrow CB, Hinkle JT, Seemiller J, et al. The Association of Antidepressant Use and Impulse Control Disorder in Parkinson's Disease. *American Journal of Geriatric Psychiatry* 2024; 32: 710-720. DOI: 10.1016/j.jagp.2023.12.024.
224. Moum SJ, Price CC, Limotai N, et al. Effects of STN and GPi deep brain stimulation on impulse control disorders and dopamine dysregulation syndrome. *PLoS One* 2012; 7: e29768. DOI: 10.1371/journal.pone.0029768.
225. Nadeau D, Giroux I, Simard M, et al. Gambling Habits of People With Parkinson's Disease: An Exploratory Study. *Journal of Gambling Issues* 2018: 149-171. DOI: 10.4309/jgi.2018.37.7.
226. Nardone R, De Blasi P, Höller Y, et al. Repetitive transcranial magnetic stimulation transiently reduces punding in Parkinson's disease: A preliminary study. *Journal of Neural Transmission* 2014; 121: 267-274. DOI: 10.1007/s00702-013-1100-3.
227. Nissen T, Newman EJ, Grosset KA, et al. Duration of L-dopa and dopamine agonist monotherapy in Parkinson's disease. *Scottish Medical Journal* 2012; 57: 217-220. DOI: 10.1258/smj.2012.012121.
228. Okai D, Askey-Jones S, Samuel M, et al. Trial of CBT for impulse control behaviors affecting Parkinson patients and their caregivers. *Neurology* 2013; 80: 792-799. DOI: 10.1212/WNL.0b013e3182840678.
229. Okai D, Askey-Jones S, Samuel M, et al. Predictors of response to a cognitive behavioral intervention for impulse control behaviors in Parkinson's disease. *Movement Disorders* 2015; 30: 736-739. DOI: 10.1002/mds.26108.
230. Olanow CW, Factor SA, Espay AJ, et al. Apomorphine sublingual film for off episodes in Parkinson's disease: a randomised, double-blind, placebo-controlled phase 3 study. *The Lancet Neurology* 2020; 19: 135-144. DOI: 10.1016/S1474-4422(19)30396-5.
231. Olley J, Blaszczynski A and Lewis S. Dopaminergic Medication in Parkinson's Disease and Problem Gambling. *Journal of gambling studies / co-sponsored by the National Council on Problem Gambling and Institute for the Study of Gambling and Commercial Gaming* 2015; 31: 1085-1106. DOI: 10.1007/s10899-014-9503-0.
232. Ondo WG and Lai D. Predictors of impulsivity and reward seeking behavior with dopamine agonists. *Parkinsonism and Related Disorders* 2008; 14: 28-32. DOI: 10.1016/j.parkreldis.2007.05.006.
233. O'Sullivan SS, Djamshidian A, Evans AH, et al. Excessive hoarding in Parkinson's disease. *Movement Disorders* 2010; 25: 1026-1033. DOI: 10.1002/mds.23016.
234. O'Sullivan SS, Loane CM, Lawrence AD, et al. Sleep disturbance and impulsive-compulsive behaviours in Parkinson's disease. *Journal of Neurology, Neurosurgery and Psychiatry* 2011; 82: 620-622. DOI: 10.1136/jnnp.2009.186874.
235. Ozdeniz Varan E and Gurvit H. Effect of Dopaminergic Therapy on Impulse Control Disorders in Patients With a Prolactinoma. *Cognitive and Behavioral Neurology* 2023; 36: 1-8. DOI: 10.1097/WNN.0000000000000320.
236. Ozer IS, Bayram E, Herdi O, et al. Daytime impulsiveness, attention, and learning in the restless legs syndrome. *Neurological Sciences* 2024; 45: 639-645. DOI: 10.1007/s10072-023-07020-3.

237. Ozkaya HM, Sahin S, Korkmaz OP, et al. Patients with acromegaly might not be at higher risk for dopamine agonist-induced impulse control disorders than those with prolactinomas. *Growth Hormone and IGF Research* 2020; 55. DOI: 10.1016/j.ghir.2020.101356.
238. Paliwal S, Mosley PE, Breakspear M, et al. Subjective estimates of uncertainty during gambling and impulsivity after subthalamic deep brain stimulation for Parkinson's disease. *Scientific reports* 2019; 9: 14795. DOI: 10.1038/s41598-019-51164-2.
239. Pallanti S, Bernardi S, Raglione LM, et al. Complex repetitive behavior: Punding after bilateral subthalamic nucleus stimulation in Parkinson's disease. *Parkinsonism and Related Disorders* 2010; 16: 376-380. DOI: 10.1016/j.parkreldis.2010.02.011.
240. Papay K, Mamikonyan E, Siderowf AD, et al. Patient versus informant reporting of ICD symptoms in Parkinson's disease using the QUIP: Validity and variability. *Parkinsonism and Related Disorders* 2011; 17: 153-155. DOI: 10.1016/j.parkreldis.2010.11.015.
241. Papay K, Xie SX, Stern M, et al. Naltrexone for impulse control disorders in Parkinson disease: a placebo-controlled study. *Neurology* 2014; 83: 826-833. DOI: 10.1212/WNL.0000000000000729.
242. Paul B, Singh G, Bansal N, et al. Gender Differences in Impulse Control Disorders and Related Behaviors in Patients with Parkinson's Disease and its Impact on Quality of Life. *Annals of Indian Academy of Neurology* 2020; 23: 632-637. DOI: 10.4103/aian.AIAN_47_20.
243. Paul BS, Aggarwal S, Paul G, et al. Impulse-control disorders and restless leg syndrome in Parkinson's Disease: Association or coexistence. *Annals of Indian Academy of Neurology* 2023; 26: 161-166. DOI: 10.4103/aian.aian_940_22.
244. Perez-Lloret S, Rey MV, Fabre N, et al. Prevalence and pharmacological factors associated with impulse-control disorder symptoms in patients with parkinson disease. *Clinical Neuropharmacology* 2012; 35: 261-265. DOI: 10.1097/WNF.0b013e31826e6e6d.
245. Pettorruso M, Fasano A, De Risio L, et al. Punding in non-demented Parkinson's disease patients: Relationship with psychiatric and addiction spectrum comorbidity. *Journal of the Neurological Sciences* 2016; 362: 344-347. DOI: 10.1016/j.jns.2016.02.016.
246. Phu AL, Xu Z, Brakoulias V, et al. Effect of impulse control disorders on disability and quality of life in Parkinson's disease patients. *Journal of Clinical Neuroscience* 2014; 21: 63-66. DOI: 10.1016/j.jocn.2013.02.032.
247. Picillo M, Phokaewvarangkul O, Poon YY, et al. Levodopa Versus Dopamine Agonist after Subthalamic Stimulation in Parkinson's Disease. *Movement Disorders* 2021; 36: 672-680. DOI: 10.1002/mds.28382.
248. Pineau F, Roze E, Lacomblez L, et al. Executive functioning and risk-taking behavior in Parkinson's disease patients with impulse control disorders. *Journal of Neural Transmission* 2016; 123: 573-581. DOI: 10.1007/s00702-016-1549-y.
249. Poletti M, Logi C, Lucetti C, et al. A single-center, cross-sectional prevalence study of impulse control disorders in Parkinson disease: Association with dopaminergic drugs. *Journal of Clinical Psychopharmacology* 2013; 33: 691-694. DOI: 10.1097/JCP.0b013e3182979830.
250. Pondal M, Marras C, Miyasaki J, et al. Clinical features of dopamine agonist withdrawal syndrome in a movement disorders clinic. *Journal of Neurology, Neurosurgery and Psychiatry* 2013; 84: 130-135. DOI: 10.1136/jnnp-2012-302684.
251. Pontieri FE, Assogna F, Pellicano C, et al. Sociodemographic, neuropsychiatric and cognitive characteristics of pathological gambling and impulse control disorders NOS in Parkinson's disease. *European Neuropsychopharmacology* 2015; 25: 69-76. DOI: 10.1016/j.euroneuro.2014.11.006.
252. Pontone G, Williams JR, Bassett SS, et al. Clinical features associated with impulse control disorders in Parkinson disease. *Neurology* 2006; 67: 1258-1261. DOI: 10.1212/01.wnl.0000238401.76928.45.
253. Porta F, Ponzone A and Spada M. Long-term safety and effectiveness of pramipexole in tetrahydrobiopterin deficiency. *European Journal of Paediatric Neurology* 2016; 20: 839-842. DOI: 10.1016/j.ejpn.2016.08.006.
254. Pourcher E, Rémillard S and Cohen H. Compulsive habits in restless legs syndrome patients under dopaminergic treatment. *Journal of the Neurological Sciences* 2010; 290: 52-56. DOI: 10.1016/j.jns.2009.11.010.
255. Rábano-Suárez P, Martínez-Fernández R, Natera-Villalba E, et al. Impulse Control Disorders in Parkinson's Disease: Has COVID-19 Related Lockdown Been a Trigger? *Movement Disorders Clinical Practice* 2021; 8: 940-943. DOI: 10.1002/mdc3.13276.
256. Rabinak CA and Nirenberg MJ. Dopamine agonist withdrawal syndrome in parkinson disease. *Archives of Neurology* 2010; 67: 58-63. DOI: 10.1001/archneurol.2009.294.
257. Rana AQ, Mansoor W, Hussaini S, et al. Factors associated with the development of impulse compulsive disorders in Parkinson patients. *International Journal of Neuroscience* 2013; 123: 503-506. DOI: 10.3109/00207454.2013.768243.

258. Redenšek S, Bizjan BJ, Trošt M, et al. Clinical and clinical-pharmacogenetic models for prediction of the most common psychiatric complications due to dopaminergic treatment in Parkinson's disease. *International Journal of Neuropsychopharmacology* 2020; 23: 496-504. DOI: 10.1093/ijnp/pyaa028.
259. Ren W, Qi Y, Liu Y, et al. Evaluation of risk factors for impulse control disorder in Parkinson’s disease in northern China. *Frontiers in Aging Neuroscience* 2023; 15. DOI: 10.3389/fnagi.2023.1257618.
260. Ricciardi L, Lambert C, De Micco R, et al. Can we predict development of impulsive-compulsive behaviours in Parkinson's disease? *Journal of neurology, neurosurgery, and psychiatry* 2018; 89: 476-481. DOI: 10.1136/jnnp-2017-317007.
261. Ricciardi L, De Angelis A, Siri C, et al. Dyskinesia and impulsive compulsive behaviour in Parkinson's disease are not related: Insights from a study with a wearable sensor. *Parkinsonism and Related Disorders* 2023; 115. DOI: 10.1016/j.parkreldis.2023.105813.
262. Rieu I, Houeto JL, Pereira B, et al. Impact of Mood and Behavioral Disorders on Quality of Life in Parkinson's disease. *Journal of Parkinson's Disease* 2016; 6: 267-277. DOI: 10.3233/JPD-150747.
263. Rizos A, Sauerbier A, Antonini A, et al. A European multicentre survey of impulse control behaviours in Parkinson's disease patients treated with short- and long-acting dopamine agonists. *European Journal of Neurology* 2016; 23: 1255-1261. DOI: 10.1111/ene.13034.
264. Rodríguez-Violante M, González-Latapi P, Cervantes-Arriaga A, et al. Impulse control and related disorders in Mexican Parkinson's disease patients. *Parkinsonism and Related Disorders* 2014; 20: 907-910. DOI: 10.1016/j.parkreldis.2014.05.014.
265. Rodríguez-Violante M, Ríos-Solís Y, Esquivel-Zapata O, et al. Assessment of therapeutic strategies for management of impulse control disorder in Parkinson's disease. *Arquivos de Neuro-Psiquiatria* 2021; 79: 989-994. DOI: 10.1590/0004-282X-ANP-2020-0507.
266. Rossi PJ, De Jesus S, Hess CW, et al. Measures of impulsivity in Parkinson's disease decrease after DBS in the setting of stable dopamine therapy. *Parkinsonism and Related Disorders* 2017; 44: 13-17. DOI: 10.1016/j.parkreldis.2017.08.006.
267. Saadat P, Faramarzi M, Salimkhani F, et al. Psychiatric symptoms in patients with Parkinson’s disease and caregivers. *Oman Medical Journal* 2020; 35: 1-22. DOI: 10.5001/omj.2020.91.
268. Sachdeva J, Harbishettar V, Barraclough M, et al. Clinical profile of compulsive sexual behaviour and paraphilia in Parkinson's disease. *Journal of Parkinson's Disease* 2014; 4: 665-670. DOI: 10.3233/JPD-140366.
269. Sahin S, Sudutan T, Kavla Y, et al. A Genetic Assessment of Dopamine Agonist-Induced Impulse Control Disorder in Patients With Prolactinoma. *Journal of Clinical Endocrinology and Metabolism* 2023; 108: e275-e282. DOI: 10.1210/clinem/dgac718.
270. Sanjan G, Das L, Ahuja C, et al. Impulse Control Disorders with Short-term Use of Cabergoline in Macroprolactinomas: A Prospective Study with a Brief Review of Literature. *Neurology India* 2023; 71: 107-112. DOI: 10.4103/0028-3886.370452.
271. Santangelo G, Vitale C, Trojano L, et al. Cognitive dysfunctions and Pathological gambling in patients with Parkinson's disease. *Movement Disorders* 2009; 24: 899-905. DOI: 10.1002/mds.22472.
272. Santin MDN, Voulleminot P, Vrillon A, et al. Impact of Subthalamic Deep Brain Stimulation on Impulse Control Disorders in Parkinson's Disease: A Prospective Study. *Movement Disorders* 2021; 36: 750-757. DOI: 10.1002/mds.28320.
273. Santos García D, Jesús S, Aguilar M, et al. COPPADIS-2015 (COhort of Patients with PArkinson's DIsease in Spain, 2015): an ongoing global Parkinson's disease project about disease progression with more than 1000 subjects included. Results from the baseline evaluation. *European Journal of Neurology* 2019; 26: 1399-1407. DOI: 10.1111/ene.14008.
274. Santos-García D, de Deus Fonticoba T, Cores Bartolomé C, et al. Depression is Associated with Impulse-compulsive Behaviors in Parkinson's disease. *J Affect Disord* 2021; 280: 77-89. DOI: 10.1016/j.jad.2020.11.075.
275. Santos-García D, Castro ES, de Deus Fonticoba T, et al. Sleep Problems Are Related to a Worse Quality of Life and a Greater Non-Motor Symptoms Burden in Parkinson’s Disease. *Journal of Geriatric Psychiatry and Neurology* 2021; 34: 642-658. DOI: 10.1177/0891988720964250.
276. Sauerbier A, Loehrer P, Jost ST, et al. Predictors of short-term impulsive and compulsive behaviour after subthalamic stimulation in Parkinson disease. *Journal of Neurology, Neurosurgery and Psychiatry* 2021; 92: 1313-1318. DOI: 10.1136/jnnp-2021-326131.
277. Scavone C, Stelitano B, Rafaniello C, et al. Drugs-Induced Pathological Gambling: An Analysis of Italian Spontaneous Reporting System. *Journal of Gambling Studies* 2020; 36: 85-96. DOI: 10.1007/s10899-019-09828-1.

278. Schreglmann SR, Gantenbein AR, Eisele G, et al. Transdermal rotigotine causes impulse control disorders in patients with restless legs syndrome. *Parkinsonism and Related Disorders* 2012; 18: 207-209. DOI: 10.1016/j.parkreldis.2011.10.010.
279. Scott BM, Eisinger RS, Burns MR, et al. Co-occurrence of apathy and impulse control disorders in Parkinson disease. *Neurology* 2020; 95: E2769-E2780. DOI: 10.1212/WNL.0000000000010965.
280. Scott BM, Eisinger RS, Rana AN, et al. From pleasure to punding: Distinct patterns of anhedonia and impulsivity linked to motivational disturbances in Parkinson disease. *APPLIED NEUROPSYCHOLOGY-ADULT* 2022. DOI: 10.1080/23279095.2022.2146506.
281. Scott BM, Eisinger RS, Mara R, et al. Co-Occurrence of Apathy and Impulse Control Disorders in Parkinson Disease: Variation across Multiple Measures. *Archives of clinical neuropsychology : the official journal of the National Academy of Neuropsychologists* 2024; 39: 907-913. DOI: 10.1093/arclin/acae036.
282. Scullin MK, Sollinger AB, Land J, et al. Sleep and impulsivity in Parkinson's disease. *Parkinsonism and Related Disorders* 2013; 19: 991-994. DOI: 10.1016/j.parkreldis.2013.06.018.
283. Shapiro MA, Chang YL, Munson SK, et al. The four as associated with pathological Parkinson disease gamblers: Anxiety, anger, age, and agonists. *Neuropsychiatric Disease and Treatment* 2007; 3: 161-167. DOI: 10.2147/nedt.2007.3.1.161.
284. Sharma A, Goyal V, Behari M, et al. Impulse control disorders and related behaviours (ICD-RBs) in Parkinson's disease patients: Assessment using "questionnaire for impulsive-compulsive disorders in Parkinson's disease" (QUIP). *Annals of Indian Academy of Neurology* 2015; 18: 49-59. DOI: 10.4103/0972-2327.144311.
285. Simoni S, Paoletti FP, Eusebi P, et al. Impulse Control Disorders and Levodopa-Induced Dyskinesias in Parkinson's Disease: Pulsatile versus Continuous Dopaminergic Stimulation. *Journal of Parkinson's Disease* 2020; 10: 927-934. DOI: 10.3233/JPD-191833.
286. Şimşek Erdem N, Güneş Gencer GY, Özkaynak SS, et al. Neuropsychiatric Effects of Bilateral Subthalamic Nucleus Deep Brain Stimulation in Parkinson’s Disease: Results at the 12-Month Follow-up. *Noropsikiyatri Arsivi* 2023; 60: 169-173. DOI: 10.29399/npa.28241.
287. Singh A, Kandimala G, Dewey Jr RB, et al. Risk factors for pathologic gambling and other compulsions among Parkinson's disease patients taking dopamine agonists. *Journal of Clinical Neuroscience* 2007; 14: 1178-1181. DOI: 10.1016/j.jocn.2007.01.009.
288. Siri C, Cilia R, De Gaspari D, et al. Psychiatric symptoms in Parkinson's disease assessed with the SCL-90R self-reported questionnaire. *Neurological Sciences* 2010; 31: 35-40. DOI: 10.1007/s10072-009-0165-0.
289. Siri C, Cilia R, De Gaspari D, et al. Cognitive status of patients with Parkinson's disease and pathological gambling. *Journal of Neurology* 2010; 257: 247-252. DOI: 10.1007/s00415-009-5301-5.
290. Siri C, Cilia R, Reali E, et al. Long-term cognitive follow-up of Parkinson's disease patients with impulse control disorders. *Movement Disorders* 2015; 30: 696-704. DOI: 10.1002/mds.26160.
291. Smith KM, Xie SX and Weintraub D. Incident impulse control disorder symptoms and dopamine transporter imaging in Parkinson disease. *Journal of Neurology, Neurosurgery and Psychiatry* 2016; 87: 864-870. DOI: 10.1136/jnnp-2015-311827.
292. Sohtaoĝlu M, Demiray DY, Kenangil G, et al. Long term follow-up of Parkinson's disease patients with impulse control disorders. *Parkinsonism and Related Disorders* 2010; 16: 334-337. DOI: 10.1016/j.parkreldis.2010.02.006.
293. Solla P, Cannas A, Floris GL, et al. Behavioral, neuropsychiatric and cognitive disorders in Parkinson's disease patients with and without motor complications. *Progress in Neuro-Psychopharmacology and Biological Psychiatry* 2011; 35: 1009-1013. DOI: 10.1016/j.pnpbp.2011.02.002.
294. Solla P, Cannas A, Ibba FC, et al. Gender differences in motor and non-motor symptoms among Sardinian patients with Parkinson's disease. *Journal of the Neurological Sciences* 2012; 323: 33-39. DOI: 10.1016/j.jns.2012.07.026.
295. Somma T, Esposito F, Scala MR, et al. Psychiatric Symptoms in Parkinson’s Disease Patients before and One Year after Subthalamic Nucleus Deep Brain Stimulation Therapy: Role of Lead Positioning and Not of Total Electrical Energy Delivered. *Journal of Personalized Medicine* 2022; 12. DOI: 10.3390/jpm12101643.
296. Somme JH, Gómez-Esteban JC, Tijero B, et al. Impulse control and repetitive behaviors in Parkinson's disease - Are there differences in the relation to dopamine agonist treatment? *Journal of the Neurological Sciences* 2014; 345: 252-253. DOI: 10.1016/j.jns.2014.07.004.
297. Staubo SC, Fuskevåg OM, Toft M, et al. Dopamine agonist serum concentrations and impulse control disorders in Parkinson's disease. *European Journal of Neurology* 2024; 31. DOI: 10.1111/ene.16144.
298. Staubo SC, Fuskevåg OM, Toft M, et al. Impulse control and correlation to dopamine agonist serum concentrations in people with Parkinson's disease. *Journal of Neurology* 2025; 272. DOI: 10.1007/s00415-024-12870-8.

299. Sulu C, Gul N, Tanrikulu S, et al. Risk of impulse control disorders in patients with Cushing's disease: do not blame cabergoline but do not give up caution. *Pituitary* 2023; 26: 495-509. DOI: 10.1007/s11102-023-01342-3.
300. Takahashi M, Koh J, Yorozu S, et al. Validation of the Japanese Version of the Questionnaire for Impulsive-Compulsive Disorders in Parkinson's Disease-Rating Scale (QUIP-RS). *Parkinson's Disease* 2022; 2022. DOI: 10.1155/2022/1503167.
301. Tanaka K, Wada-Isoe K, Nakashita S, et al. Impulsive compulsive behaviors in Japanese Parkinson's disease patients and utility of the Japanese version of the Questionnaire for Impulsive-Compulsive Disorders in Parkinson's disease. *Journal of the Neurological Sciences* 2013; 331: 76-80. DOI: 10.1016/j.jns.2013.05.013.
302. Tang X, Liang Q, Li T, et al. Excessive Daytime Sleepiness as a Risk Factor for Impulse Control Disorders in Parkinson's Disease. *Neuropsychiatric Disease and Treatment* 2024; 20: 2517-2527. DOI: 10.2147/NDT.S485339.
303. Tayim N, Panicker JN, Foley J, et al. A qualitative study exploring the clinical phenomenology and impact of hypersexuality in patients with Parkinson's Disease. *Scientific reports* 2024; 14: 28697. DOI: 10.1038/s41598-024-79966-z.
304. Tayim N, Panicker J, Foley J, et al. Impact of hypersexuality on spousal carers of patients with Parkinson's disease and frontotemporal dementia: a qualitative study. *BMJ Open* 2025; 15: e090870. DOI: 10.1136/bmjopen-2024-090870.
305. Terenzi D, Rumiati RI, Catalan M, et al. Reward sensitivity in Parkinson's patients with binge eating. *Parkinsonism and Related Disorders* 2018; 51: 79-84. DOI: 10.1016/j.parkreldis.2018.03.007.
306. Thomas A, Bonanni L, Gambi F, et al. Pathological gambling in parkinson disease is reduced by amantadine. *Annals of Neurology* 2010; 68: 400-404. DOI: 10.1002/ana.22029.
307. Thomson CJ, Segrave RA and Carter A. Changes in Personality Associated with Deep Brain Stimulation: a Qualitative Evaluation of Clinician Perspectives. *Neuroethics* 2021; 14: 109-124. DOI: 10.1007/s12152-019-09419-2.
308. Todorova A, Samuel M, Brown RG, et al. Infusion Therapies and Development of Impulse Control Disorders in Advanced Parkinson Disease: Clinical Experience after 3 Years' Follow-up. *Clinical Neuropharmacology* 2015; 38: 132-134. DOI: 10.1097/WNF.0000000000000091.
309. Tolosa E, Ebersbach G, Ferreira JJ, et al. The Parkinson's Real-World Impact Assessment (PRISM) Study: A European Survey of the Burden of Parkinson's Disease in Patients and their Carers. *J Parkinsons Dis* 2021; 11: 1309-1323. DOI: 10.3233/jpd-212611.
310. Toś M, Grażyńska A, Antoniuk S, et al. Impulse Control Disorders in the Polish Population of Patients with Parkinson's Disease. *Medicina (Kaunas, Lithuania)* 2023; 59. DOI: 10.3390/medicina59081468.
311. Toś M, Grażyńska A, Antoniuk S, et al. Impulse Control Disorders in Parkinson's Disease and Atypical Parkinsonian Syndromes—Is There a Difference? *Brain Sciences* 2024; 14. DOI: 10.3390/brainsci14020181.
312. Trovini G, Lombardozzi G, Kotzalidis GD, et al. Partial Dopamine D2/3 Agonists and Dual Disorders: A Retrospective-Cohort Study in a Real-World Clinical Setting on Patients with Schizophrenia Spectrum Disorders and Cannabis Use Disorder. *Current neuropharmacology* 2025. DOI: 10.2174/011570159X350599241214042724.
313. Turcano P, Jacobson J, Ghoniem K, et al. Impulse control disorders and use of dopamine agonists in early onset Parkinson's disease. *Frontiers in Neurology* 2024; 15. DOI: 10.3389/fneur.2024.1404904.
314. Umemura A, Oka Y, Yamamoto K, et al. Complications of subthalamic nucleus stimulation in Parkinson's disease. *Neurologia Medico-Chirurgica* 2011; 51: 749-755. DOI: 10.2176/nmc.51.749.
315. Valença GT, Glass PG, Negreiros NN, et al. Past smoking and current dopamine agonist use show an independent and dose-dependent association with impulse control disorders in Parkinson's disease. *Parkinsonism and Related Disorders* 2013; 19: 698-700. DOI: 10.1016/j.parkreldis.2013.03.004.
316. Vargas AP, Vaz LS, Reuter A, et al. Impulse control symptoms in patients with Parkinson's disease: The influence of dopaminergic agonist. *Parkinsonism and Related Disorders* 2019; 68: 17-21. DOI: 10.1016/j.parkreldis.2019.06.019.
317. Varshney A, Kumar P and Ram VS. Nonmotor Symptoms and Impulse Control Disorders in Parkinson's Disease Patients in India: A Cross-sectional Study. *Annals of African Medicine* 2024; 23: 400-405. DOI: 10.4103/aam.aam_198_23.
318. Veiga BAAG, da Silva CC, Saba RA, et al. Are impulsive compulsive behaviors associated with levodopa-induced dyskinesia? A Brazilian cross-sectional study. *Clinical Neurology and Neurosurgery* 2025; 249. DOI: 10.1016/j.clineuro.2024.108708.
319. Vela L, Martínez Castrillo JC, García Ruiz P, et al. The high prevalence of impulse control behaviors in patients with early-onset Parkinson's disease: A cross-sectional multicenter study. *Journal of the Neurological Sciences* 2016; 368: 150-154. DOI: 10.1016/j.jns.2016.07.003.

320. Verbaan D, van Rooden SM, Visser M, et al. Psychotic and compulsive symptoms in Parkinson's disease. *Movement Disorders* 2009; 24: 738-744. DOI: 10.1002/mds.22453.
321. Vitale C, Santangelo G, Trojano L, et al. Comparative neuropsychological profile of pathological gambling, hypersexuality, and compulsive eating in Parkinson's disease. *Movement Disorders* 2011; 26: 830-836. DOI: 10.1002/mds.23567.
322. Voon V, Hassan K, Zurowski M, et al. Prevalence of repetitive and reward-seeking behaviors in Parkinson disease. *Neurology* 2006; 67: 1254-1257. DOI: 10.1212/01.wnl.0000238503.20816.13.
323. Voon V, Hassan K, Zurowski M, et al. Prospective prevalence of pathologic gambling and medication association in Parkinson disease. *Neurology* 2006; 66: 1750-1752. DOI: doi:10.1212/01.wnl.0000218206.20920.4d.
324. Voon V, Thomsen T, Miyasaki JM, et al. Factors associated with dopaminergic drug-related pathological gambling in Parkinson disease. *Archives of Neurology* 2007; 64: 212-216. DOI: 10.1001/archneur.64.2.212.
325. Voon V, Reynolds B, Brezing C, et al. Impulsive choice and response in dopamine agonist-related impulse control behaviors. *Psychopharmacology* 2010; 207: 645-659. DOI: 10.1007/s00213-009-1697-y.
326. Voon V, Sohr M, Lang AE, et al. Impulse control disorders in Parkinson disease: A multicenter case–control study. *Annals of Neurology* 2011; 69: 986-996. DOI: 10.1002/ana.22356.
327. Voon V, Schoerling A, Wenzel S, et al. Frequency of impulse control behaviours associated with dopaminergic therapy in restless legs syndrome. *BMC Neurology* 2011; 11. DOI: 10.1186/1471-2377-11-117.
328. Wang XP, Wei M and Xiao Q. A survey of impulse control disorders in Parkinson's disease patients in Shanghai area and literature review. *Translational Neurodegeneration* 2016; 5. DOI: 10.1186/s40035-016-0051-7.
329. Waskowiak P, Koppelmans V and Ruitenberg MFL. Trait Anxiety as a Risk Factor for Impulse Control Disorders in de novo Parkinson's Disease. *Journal of Parkinson's Disease* 2022; 12: 689-697. DOI: 10.3233/JPD-212959.
330. Weintraub D, Siderowf AD, Potenza MN, et al. Association of dopamine agonist use with impulse control disorders in Parkinson disease. *Archives of Neurology* 2006; 63: 969-973. DOI: 10.1001/archneur.63.7.969.
331. Weintraub D, Sohr M, Potenza MN, et al. Amantadine use associated with impulse control disorders in Parkinson disease in cross-sectional study. *Annals of Neurology* 2010; 68: 963-968. DOI: 10.1002/ana.22164.
332. Weintraub D, Koester J, Potenza MN, et al. Impulse Control Disorders in Parkinson Disease: A Cross-Sectional Study of 3090 Patients. *Archives of Neurology* 2010; 67: 589-595. DOI: 10.1001/archneurol.2010.65.
333. Weintraub D, Papay K and Siderowf A. Screening for impulse control symptoms in patients with de novo Parkinson disease: a case-control study. *Neurology* 2013; 80: 176-180. DOI: 10.1212/WNL.0b013e31827b915c.
334. Wicks P and MacPhee GJA. Pathological gambling amongst Parkinson's disease and ALS patients in an online community (PatientsLikeMe.com). *Movement Disorders* 2009; 24: 1085-1088. DOI: 10.1002/mds.22528.
335. Wirth T, Goetsch T, Corvol JC, et al. Prognosis of impulse control disorders in Parkinson’s disease: a prospective controlled study. *Journal of Neurology* 2024; 271: 2412-2422. DOI: 10.1007/s00415-023-12170-7.
336. Wolfschlag M and Håkansson A. Increased risk for developing gambling disorder under the treatment with pramipexole, ropinirole, and aripiprazole: A nationwide register study in Sweden. *PLoS One* 2021; 16. DOI: 10.1371/journal.pone.0252516.
337. Wolfschlag M, Weber GC, Weintraub D, et al. Impulse control disorders in Parkinson’s disease: a national Swedish registry study on high-risk treatments and vulnerable patient groups. *Journal of Neurology, Neurosurgery and Psychiatry* 2024. DOI: 10.1136/jnnp-2024-334116.
338. Wolfschlag M, Cedergren Weber G, Timpka J, et al. Recognition, management, and patient perspectives of impulsive-compulsive disorders in Parkinson's disease. *Journal of Parkinson's Disease* 2025: 1877718X251323922. DOI: 10.1177/1877718X251323922.
339. Wu K, Politis M, O'Sullivan SS, et al. Problematic Internet use in Parkinson's disease. *Parkinsonism and Related Disorders* 2014; 20: 482-487. DOI: 10.1016/j.parkreldis.2014.01.019.
340. Wu HF, Yu ELM, Sheng B, et al. Impulse control and related behavioral disorders (ICRD) in Idiopathic Parkinson's Disease treated with different dopamine agonists in Hong Kong: Is any dopamine agonist better? *Clinical Parkinsonism and Related Disorders* 2024; 10. DOI: 10.1016/j.prdoa.2024.100235.

341. Xu T, Cao L, Long W, et al. Validation of the Chinese Version of the Questionnaire for Impulsive-Compulsive Disorders in Parkinson's Disease. *Frontiers in Neurology* 2021; 12. DOI: 10.3389/fneur.2021.731552.
342. Ye M, Kang X, Karlsson IK, et al. Associations between Sleep Disorders and Impulsive-Compulsive Behaviors in Parkinson's Disease: A Prospective Cohort Study. *Neuroepidemiology* 2024; 58: 256-263. DOI: 10.1159/000536555.
343. Yildirim E, Altinayar S and Cakmur R. Decision-making and impulse-control disorders in parkinson's disease: Influence of dopaminergic treatment. *Neurological Sciences and Neurophysiology* 2020; 37: 11-17. DOI: 10.4103/NSN.NSN_12_20.
344. Zahodne LB, Susatia F, Bowes D, et al. Binge eating in Parkinson's disease: Prevalence, correlates and the contribution of deep brain stimulation. *Journal of Neuropsychiatry and Clinical Neurosciences* 2011; 23: 56-62. DOI: 10.1176/appi.neuropsych.23.1.56.
345. Zazu L, Morera-Herreras T, Garcia M, et al. Do cariprazine and brexpiprazole cause impulse control symptoms? A case/non-case study. *European Neuropsychopharmacology* 2021; 50: 107-111. DOI: 10.1016/j.euroneuro.2021.05.001.
346. Zhang Y, He A, Li L, et al. Clinical characteristics of impulse control and related disorders in Chinese Parkinson's disease patients. *BMC Neurology* 2017; 17. DOI: 10.1186/s12883-017-0874-6.
347. Zhang Y, Zhu XB, Gan J, et al. Impulse control behaviors and apathy commonly co-occur in de novo Parkinson's disease and predict the incidence of levodopa-induced dyskinesia. *Journal of Affective Disorders* 2024; 351: 895-903. DOI: 10.1016/j.jad.2024.02.013.
348. Zhong M, Peppard R, Velakoulis D, et al. The relationship between specific cognitive defects and burden of care in Parkinson's disease. *International Psychogeriatrics* 2016; 28: 275-281. DOI: 10.1017/S1041610215001593.
349. Zhu X, Gan J, Wu N, et al. Assessing impulse control behaviors in early Parkinson's disease: a longitudinal study. *Frontiers in Neurology* 2023; 14. DOI: 10.3389/fneur.2023.1275170.
