# Supplementary material for: Clinical aspects of impulsive-compulsive behaviors under dopaminergic treatment—A scoping review
Source: Australas Psychiatry. 2026 Mar 28;34(5):432–6. doi: 10.1177/10398562261439053 (PMC13669450; doi:10.1177/10398562261439053)
Supplement: Supplementary file 1 [file sj-zip-1-apy-10.1177_10398562261439053.zip › Supplementary file S4_Included studies_Results.pdf]

Abbreviations

**AE**	Adverse event
**ALS**	Amyotrophic lateral sclerosis
**BE**	Binge eating
**BIS-11**	Barratt impulsiveness scale
**BL**	Baseline
**CB**	Compulsive buying
**CSB**	Compulsive sexual behavior
**COMT-I**	COMT inhibitor
**DA**	Dopamine agonist
**DAWS**	Dopamine agonist withdrawal syndrome
**DBS**	Deep brain stimulation
**DDS**	Dopamine dysregulation syndrome
**ER**	Extended-release
**FU**	Follow-up
**GPi**	Globus pallidus interna
**HC**	Healthy control
**ICB**	Impulsive-compulsive behaviour
**ICD**	Impulse control disorder (PG, CSB, CB, BE)
**IGT**	Iowa gambling task
**IR**	Immediate-release
**LCIG**	Levodopa-carbidopa intestinal gel
**LD**	Levodopa
**LEDD**	Levodopa equivalent daily dose
**MAOB-I**	MAO-B inhibitor
**OCD**	Obsessive-compulsive disorder
**PD**	Parkinson's disease
**PG**	Pathological gambling
**PPX**	Pramipexole
**QOL**	Quality of life
**QUIP**	Questionnaire for Impulsive-Compulsive Disorders in Parkinson’s Disease
**RBD**	REM sleep behavior disorder
**RCT**	Randomized controlled trial
**RLS**	Restless legs syndrome
**ROP**	Ropinirole
**STN**	Subthalamic nucleus

#	First author Year	Main findings: Prevalence, Impact	Main findings: Risk factors	Main findings: Comorbidity	Main findings: Management
A1	Abbes 2018		No increase in any ICB under STN DBS at 3-10 yrs follow-up after surgery		Decreased hobbyism, PG, CB, risk-seeking behaviour and dopaminergic addiction under STN DBS at 3-10 yrs FU after surgery; no effect on punding, CSB, BE
A2	Abosch 2011		General ICB prevalence higher in women; CB, kleptomania, and trichotillomania more common in women; intermittent explosive behavior, compulsive exercise, and CSB more common in men; men more likely to report multiple ICBs.<br><br>Younger age at screening and PD onset an ICB risk factor.		
A3	Ahearn 2012	Prevalence: 35% met DSM-IV criteria for at least one ICD<br><br>-PG 37%, CSB 29%, shopping 29%, BE 23%, DDS 9%	No sex difference in BIS-11 score, but higher score correlated with younger PD onset age and higher LEDD. No correlation with education level, DA LEDD and disease duration.	Impulsivity/ICDs were linked to higher anxiety, higher PG severity, greater motor complications and neuropsychiatric features of aggression, elation, irritability & disinhibition	
A4	Almalki 2024	Prevalence: 52 % reported ≥1 ICD item on the modified questionnaire.<br><br>One ICD type in 33 %, two in 12 %, three in 7 %.<br><br>CB 28.5 %, punding 24.5 %, CSB 24.5 %, PG 0.5 %.	Male patients: higher frequency of CSB. No sex effect on ICDs in general.<br>Trend for ICD patients to be younger.<br>No effect of treatment duration, bromocriptine or cabergoline use or dose.	Independent predictors (multivariable logistic regression): personal history of psychiatric illness (mostly depression/anxiety) — AOR 7.67 ($p = 0.021$).	
A5	Alvarado-Bolaños 2015	Prevalence: ICD 27 % (male: 61.7%,female: 38.3%)<br><br>In the final multiple-regression model predicting PDQ-8 (QoL), ICD severity remained a significant independent predictor ($\beta = 0.08$, $p = 0.04$) after mood/apathy, motor	With ICD: more DA use, higher DA LEDD		

		severity (MDS-UPDRS III), age and disease duration were controlled for.			
A6	Amami 2015		ICB patients: higher baseline doses of DAs, no differences regarding dosage of LD, amantadine or MAOB-Is, sex, age at onset, age at surgery and disease duration. 6/43 developed de novo ICB during, but all resolved until 3 years follow-up (0/43 with de novo ICB).		11/13 (84.6%) with ICB before DBS had no behavioural symptoms 3 years after DBS start (including 1 temporary relapse). Other 2 had a new ICB that replaced the old one. Clear DA dose reduction in ICB group after 3 years DBS.
A7	Amstutz 2025		With ICD: longer disease duration, higher LEDD, higher DA dose.		Reduction in QUIP-RS score after DBS.
A8	Angioletti 2020		No difference in age, LD dose, LEDD or % of DA on LEDD between patients with and without PG.		
A9	Antonini 2016		No rotigotine dose effect on ICDs, but higher LD dose. Patients with ICDs younger.		In 17 of 71 patients ICD led to a reduction in rotigotine dose (23.9%); ICD resolved in 14 patients.
A10	Antonini 2017	Prevalence: ICD ~28 % at BL, 29 % at year 1, 26.5 % at year 2.<br><br>Most common subtype: BE (≈ 12 %), followed by punding and CSB.<br><br>Prevalence (QUIP): slightly higher (~34 %, 35 %, 32 %).<br><br>Number of behaviours: majority had a single ICD; 8–10 % (mMIDI) or 15 % (QUIP) exhibited ≥ 2 subtypes.<br><br>Impact: ICD-positive patients reported significantly worse quality of life (PDQ-8), poorer sleep (PDSS-2) and higher depression scores (BDI-II).	With ICD: male sex, younger age, younger at PD onset, longer disease duration.<br>ICD prevalence highest under LD+DA treatment, similar under LD and DA monotherapy.	Significantly associated with ICD positivity at BL:<br><br>- Higher depression, greater overall non-motor burden (NMSS), especially sleep/fatigue, mood/apathy, attention/memory, sexual function.<br><br>- More neuropsychiatric symptoms (NPI-3); apathy/indifference most common.<br><br>-Lifestyle & social: current smoking, regular alcohol, unmarried status, and lower educational attainment were over-represented.	
A11	Aoki 2019	ICB prevalence: 45 % positive on any J-QUIP item;<br><br>Punding/hobbyism present in 41 % (33/80).	With ICB: higher LEDD, older. No difference in sex distribution or DA use.	Motor disability & longer disease duration were greater in ICB group.<br><br>Within punding or hobbyism subgroup, greater FFT associated	

				with greater lateral trunk flexion (LFT) and poorer ADL function.	
A12	Ardouin 2006				BL: DA reduction not possible or not effective. Behavioral therapy (n=4) and treatment with clozapine (n=1) ineffective.<br><br>Mean dopamine equivalent dose was decreased by 74% 3 months after surgery. In all patients, PG resolved postoperatively after 18 months on average (range, 0 – 48), although transient worsening occurred in two.
A13	Ataoglu 2024	Prevalence: 43% screened positive on QUIP	Higher total LEDD in ICD group. No difference in age, sex, disease duration, education level, LD LEDD, or DA LEDD in ICD group.	ICD comorbidities: Depression (higher BDI) – independent association	
A14	Aumann 2020		No difference in disease duration. ICB+ patients seem younger and more often male (not tested?).		
A15	Auyeung 2011	Overall ICD prevalence = 7.0 %<br><br>Sub-types: PG 6.1 %; CSB 3.8 %; CB 0.5 %; BE 0.5 %<br><br>53 % had ≥ 2 ICD behaviours.	With ICD: younger, younger at PD onset, more often male, longer disease duration, higher LEDD, higher DA LEDD, more often on LD+DA.	Psychiatric comorbidity: history of clinically-diagnosed anxiety or depression strongly associated with ICD<br><br>Motor complications: dyskinesia more common in ICD group on univariate analysis but not retained in multivariate model<br><br>No association with hallucinations, substance/alcohol misuse, or family psychiatric history was found.	
A16	Avanzi 2006	Prevalence of PG in PD=6.1% (50% men and 50% women) vs 0.25% in controls; all patients started PG after the onset of PD	No single dopaminergic agent or dose emerged as an independent predictor in this small sample.		
A17	Ávila 2011				15/18 discontinued DA treatment or decreased DA dosage. 13/15 reported full/partial remission, 2 unchanged. Remission also in some

					without change to medication.<br><br>11 prescribed antidepressants, benzodiazepines or quetiapine or their doses were increased, with no relation to changes in ICBs symptoms.<br><br>11 with punding, symptoms unchanged in 9 patients independently of changes in dopaminergic drugs (lower doses of DA in 4 cases, same doses of DA or LD in other 4 patients; higher dose of DA in one patient.<br><br>Remission with punding rarer than with other ICBs.
A18	Azmin 2016	Prevalence of ICB=11.3%;<br>Most frequent behaviours: compulsive medication use 7.5 %; hobbyism 6.3 %; CSB 5 %; CB 3.8 %; punding 2.5 %; walkabout 2.5 %; BE 1.3 %; PG 1.3 %	Higher total LD dose but not DA dose showed association with ICBs		
A19	Baig 2019	The prevalence of PD-ICB was estimated at 19.1% (95% confidence interval 10.1–28.2).ICD prevalence in the RBD group (1%) was similar to that in controls (0.7%) Hobbyism was the most common PD-ICB domain, with PG and walkabout the least common. BE, shopping, and CSB were reported at similar frequencies.	DA use associated with PD-ICB	severity of motor complications (MDS-UPDRS IV score), and apathy were associated with PD-ICB	There was no significant relationship between change in LEDD or DA LEDD and change in ICB severity.
A20	Bancos 2014	Prevalence of any ICD: 24.7 % of DA-treated prolactinomas vs 17.1 % controls (P=0.31).<br><br>CSB significantly higher with DA therapy (12.99 % vs 2.86 %).<br><br>Case interviews revealed ICS	In treated patients: ICDs associated with younger age. No relationship of ICDs with sex, DA type or treatment duration.<br>CSB more common under treatment, no difference in other ICDs.	No effect of psychiatric disorders. Pituitary factors (tumour size, surgery, other hormonal deficits) and psychiatric history were not linked to ICD occurrence.	Out of 23, 6 ICD patients had already been off DAs at the time of interview, and 8 patients planned to discuss lowering the dose or other therapeutic options.

		consequences in terms of financial losses, job loss, marital breakdown. Symptoms often abated after DA dose reduction or withdrawal.			
A21	Barake 2014		Total BIS-11 score similar between the three study groups.<br>Higher cabergoline dose correlated with higher impulsivity. No effect of treatment duration.		
A22	Barbosa 2017		With ICB: more often male, younger PD onset. No difference in disease duration, apomorphine treatment duration or dose.<br>1 patient with new-onset ICB after apomorphine initiation.		6 of 12 ICB patients experienced complete resolution of their ICBs and/or addictive behaviors after reduction (n=2) or withdrawal (n=4) of the DA prior to apomorphine treatment.<br><br>Other 6: After APO initiation, ICB resolved completely (n=1), partially (n=3), or not at all (n=2).<br><br>DA LEDD lower after APO start.
A23	Barbosa 2018		With DDS:younger age at PD onset and longer disease duration. No sex difference.	With DDS: Higher prevalence of dyskinesias, no effect of depression.	All patients with DDS received neurological and psychiatric care. 9/17 patients improved completely, 7 partially, and in 1 patient DDS remained unchanged until death. All patients had alteration of their dopaminergic therapy as a therapeutic measure. LD was reduced in 10 cases; cessation of DA was done in 4 cases whereas the DA was reduced in 3 patients.<br><br>Successful remission: Trend for lower lifetime cumulative LD LEDD. No differences in LEDD at DDS onset and at death, or duration of DA treatment. Patients who failed to achieve remission were exposed to higher DA doses and had longer duration of DDS.

A24	Barbosa 2018		CSB associated with male sex and younger age. No difference in LD use, DA use, MAOB-I, amantadine, COMT-I.<br><br>With CSB: Higher total LEDD, LD dose, COMT-I dose, COMT-I + LD dose. No difference in DA dose or MAOB-I dose.		
A25	Barbosa 2019		With ICB: higher total LEDD at death, trend for higher DA use in late stage, trend for higher DA LEDD. No difference in MAOB-I use or duration of DA use, cumulative lifetime LD dose.		
A26	Barbosa 2022		Larger number with apomorphine developed ICBs, but only 2 de novo cases of ICBs during apomorphine use. 7 patients developed behavioral addictions before starting treatment with the apomorphine pump.		Apomorphine infusion possibly a safe option in ICD patients
A27	Barbosa 2022		With punding or hobbyism: No differences in sex, age at PD onset, disease duration, DA use, amantadine, MAOB-I, DBS, total LEDD, DA LEDD. Trend for younger age with hobbyism.	Punding in PD was associated with frontal executive deficits, higher anxiety and motor/non-motor burden.	
A28	Barone 2019	Incidence:<br>– year-1: 13.7% new cases<br>– cumulative 2-year: 20.6 %<br><br>Each year about 9-10 % of whole cohort had new ICD cases, but 9-11 % remitted, prevalence therefore remained stable.	De novo ICD more often in: male, younger, younger at onset, longer disease duration	Higher BL BDI-II scores predicted incident ICDs. ICD-positive at BL previously shown to have more depression & non-motor symptoms.<br><br>Sleep: worse PDSS-2 (sleep disturbance) associated with later ICD onset.<br><br>Smoking identified as behavioural comorbidity raising risk.<br><br>No significant BL differences in cognition (MMSE, FAB, PD-CRS) or	

				overall non-motor symptom burden (PD-NMSS total).	
A29	Bastiaens 2013	39.1 % developed a new-onset ICD during FU — incidence density 1 case /100 person-months of DAA exposure.<br><br>Most frequent ICD:BE, followed by CSB, CB, PG.<br><br>50 % had ≥2 ICDs.	BL: no difference in sex, age, age of PD onset, disease duration, dopaminergics use and doses.<br>FU: higher max DA dose in ICD group. Disease duration, DA treatment duration, cumulative DA exposure, specific DA, concomitant LD usage, LD dosage, total LEDD, and duration of dopaminergic therapy did not differ.	Patients with ICD had higher lifetime caffeine use, cigarette smoking and a trend toward more alcohol.<br><br>Motor complications (dyskinesia / wearing-off / off-dystonia) more common at BL in ICD pos .<br>Mood/cognitive: slightly higher MMSE (29.6 vs 28.9) in ICD pos, but similar BAI-anxiety & BDI-depression scores<br><br>Trend for family history of depression.	Of 18 with ICD: ICDs resolved in all 10 subjects who discontinued DA therapy; in 3 of 5 subjects who reduced their DA dose; and in 0 of 3 subjects who continued the same dosage of DA. Similar effect on punding.<br><br>6 experienced DAWS when reducing/stopping DA, 4 developed DDS as a consequence. None of the 6 without ICD who tapered a DA developed DAWS.
A30	Baumann-Vogel 2015	Self- vs caregiver-reported prevalence:<br>• CSB 55 % (caregiver) vs 17 % (patient).<br>• DDS 31 % vs 3 %.<br>• Punding 22 % vs 9 %.<br>Conclusion: patients underestimate both presence and severity of several ICBs			
A31	Bayard 2013		No differences in impulsivity or ICDs between any groups. No difference in age, onset age, sex, disease duration in RLS with or without ICD. More advantageous choices in IGT by controls than either of the RLS groups, no effect of DA treatment.		
A32	Bayard 2014	ICD prevalence: 21.4 %<br>Sub-types: punding 14.3 %, CSB 7.1 %, CB 4.1 %, PG 2 %, BE 2 %; ~8 % had ≥2 ICDs.		After adjustment for age, sex, PD duration, severity, LEDD, depression, neither clinical nor PSG-confirmed RBD was associated with ICDs	
A33	Beccuti 2021	Any ICD or related behaviour positive in 52 % of DA-exposed vs 31 % of controls ($p < 0.01$).<br><br>Any ICD: 46 % vs 24 % ($p < 0.01$).	More ICBs under DA treatment. No DA dose or treatment duration effect.<br>DA use and higher age ICD		

		Any related behaviour: 31 % vs 17 % ($p < 0.05$).<br><br>Specific ICDs with significant excess in DA group:<br>– CSB 27 % vs 14 % ($p < 0.04$)<br>– punding 20 % vs 7 % ($p < 0.02$)	predictors.<br>Male sex predictor of CSB.		
A34	Bentivoglio 2013		With ICD: no difference in total LEDD, levodopa LEDD, DA LEDD, age, education, disease duration. More men but not significant.	Impulsivity is the key behavioural correlate: BIS-11 elevation in ICD group.<br><br>No significant group differences in depression (Hamilton-D) or anxiety (Hamilton-A).<br><br>Severe ICD cases sometimes co-occurred with psychotic symptoms (hallucinations/delusions) requiring atypical antipsychotics.	
A35	Benussi 2017				Cathodal vs anodal vs sham transcranial direct current stimulation (tDCS) over the right dorsolateral prefrontal cortex. No significant differences of impulsivity and gambling behaviour, as measured by BIS, Gambling Symptoms Assessment Scale and South Oaks Gambling Screen, were found between groups. In IGT: better performance after cathodal stimulation compared to sham.

A36	Bermejo 2010				Patients had had ICD for at least 1 year. Adaptation of LD and/or DA therapy had not improved ICD. Treatment with zonisamide for at least 120 days at 200 mg/day.<br><br>There was a marked reduction in the severity of impulsive behaviours and global impulsiveness. Although ICDs did not disappear completely, most patients subjectively improved and remained on zonisamide after the study was complete.
A37	Bharmal 2010		With PG: younger, no difference in disease duration. Patients who did not gamble (recreationally) prior to treatment did not develop PG with medication.<br>No PG on LD only, PG more prevalent under DA treatment vs LD. Highest prevalence of PG under PPX (non-significant).		Treatment included changing from one DA to another, lowering the DA dose, discontinuation of the DA, counseling, and/or participation in support groups (GA).<br>Switching/discontinuation of DA resolved PG in 5/5 cases. Switching from PPX to ROP seemed beneficial.
A38	Binck 2020	ICD in PD overall: 11.5 %, markedly higher in DA+ than DA⁻- 15.4 % vs 6.9 %; p = 0.005).DA-patients and controls showed similar low rates (6.9 % vs 4.6 %; n.s.).<br><br>Most common ICD subtype in men was BE and in women CB.	BL: no significant difference in ICD frequency<br>At visit 3, ICD frequency >4-fold higher in DA+ vs DA- while stable in DA- group		
A39	Biundo 2017	Prevalence<br>-any ICD/IRB: 55 %.<br>-clinically significant cases (QUIP-RS above published cut-offs): 36 % (n = 89).<br>-sub-type distribution within clinically significant group: single ICD 28 %, single related behaviour (hobbyism, punding, DDS) 38 %, multiple ICD/RB 34 %.	In group with only ICDs (excluding related behaviours), QUIP-RS total score linearly correlated with DA dose		
A40	Bostwick 2009		PG or CSB developed only under therapeutic doses of PPX/ROP, no		

			other medication strategies.<br>With PG/CSB: younger, no sex difference.		
A41	Brigas 2024	56 % of respondents reported at least one ICB on QUIP.<br><br>-BE 22.6 %,<br>-compulsive shopping 11.3 %,<br>-DDS 11.3 %,<br>-CSB 11.3 %,<br>-hobbyism 9.4 %,<br>-PG 5.7 %.<br><br>Among ICB-positive patients: 36.6 % were current DA users, 15.5 % past DA users, 15.5 % had never taken a DA		Psychiatric symptoms were associated with the prevalence of ICB	
A42	Bril 2017	ICDs present in 22 % of sample<br>BE: 7 %		BE remained an independent predictor of greater body weight, unrelated to depression, cognitive status, constipation, or dopaminergic medication dose in the model.	
A43	Cabrini 2009		DDS associated with younger age, younger onset age, higher total LEDD. No difference in disease duration or sex.<br>CSB more common in men.		
A44	Callesen 2014	Prevalence<br>- any ICD/ICB ever during PD: 35.9 % (n = 176)<br>- current ICB symptoms: 14.9 % (n = 73)<br>-multiple ICBs common: 23 % of whole cohort reported ≥2 behaviours.<br>-individual behaviours (ever):<br>hobbyism 18 %,<br>CSB 9 %,<br>punding 8 %,<br>BE 7 %,<br>PG 5 %,	Hobbyism and CSB: more men.<br>ICB predictors: younger age at PD onset	-symptoms of depression are associated with ICBs<br>-closely related to depression is the personality trait neuroticism, which appears to be the most important personality factor in ICBs in PD.	

		CB 5 %,<br>walkabout 4 %,<br>compulsive medication use 7 %.			
A45	Callesen 2017	35.9% of total sample reported symptoms of one (12.6% of total sample) or multiple ICBs (23.3% of total sample).<br><br>Hobbyism 16.7 % Punding 10.8 %<br><br>Time burden: 10.6 % of those with hobbyism/punding spent > 5 hours/day (1.8 % > 10 h).<br><br>Only 4.8 % (single-ICB) to 26.3 % (multiple-ICBs) had ever told a health-care professional. Men far more likely to disclose, no woman disclosed hobbyism/punding specifically	In both male/female with ICD: younger age, younger onset age. No difference in DA LEDD, total LEDD. Only in male with ICD: longer disease duration.<br>Hobbyism more common in men, same trend for punding.		
A46	Canesi 2020		No ICDs in drug-naive PD patients. With ICD: longer disease duration, younger onset age, longer and higher-dose LD treatment, more COMT-I use, higher total LEDD.		
A47	Cao 2020	ICD prevalence at BL(drug-naive) 12.3 %.<br><br>pRBD independently associated with existing ICDs (adjusted OR 2.56, 95 % CI 1.38-4.76).<br><br>Longitudinal:<br>25 % developed new ICDs over 5 years. BL pRBD predicted incident ICDs (HR 1.65, 95 % CI 1.05-2.58). Risk remained when stratified for dopamine-agonist (DA) treatment.	ICDs before DA therapy start common, but more common with DA use. With ICD: younger, younger onset age. No difference in sex, disease duration.	Anxiety and depression scores were higher in those who developed ICDs.	
A48	Carbunaru 2018	ICD Prevalence: 18.9% had ICDs. BE 7.5%, CSB 7.5%, shopping 7.5%,	Different QUIP-RS subscores associated with longer disease	Comorbid sleep disorders (such as RBD, OSA, PLMD) were very common (81.1%) but not associated	

		PG 3.8%, and hobbyism/punding 15.1%.	duration, DA use, PPX use, rotigotine use, MAOB-I use.	with overall ICD scores.<br>A minor association was observed between<br>OSA and thinking about PG and<br>PBD and thinking about punding.<br><br>Antidepressants (32.1%) and sleep inductors (15.1%) were significantly associated with higher ICD scores.	
A49	Castrioto 2015				3 months post OP LEDD decreased by 74%. 8/20 had ICD pre OP, remission in 7/8 post OP. IGT performance improved at 3 months post OP.
A50	Catalán 2013				In 4 patients, attempts at reducing DA resulted in intolerable motor disability and fluctuations. In the other 4 patients, DA were withdrawn 6 to 12 months prior to LCIG resulting in motor deterioration with no improvement of the psychiatric manifestations.<br><br>Motor complications improved in all patients. Nearly complete ICB symptom resolution in all patients. Less improvement in punding.
A51	Catalan 2018				LCIG infusion was associated with progressive and significant improvements in overall ICD symptoms over the study period. Reduction individually also for BE, hobbyism, punding, DDS.<br><br>Better improvement associated with longer disease duration, weaker improvement with higher age.
A52	Celik 2018	BL prevalence of ICD (before DA): 0 %<br>After DA exposure (cabergoline):<br>- new-onset ICDs in 2/25 patients (8	Cumulative DA dose, sex, and age not correlated to impulsivity scores		

		%) during the 12-month period (both with CSB)			
A53	Cera 2014				Shift towards less risky choices at follow-up in PG patients under amantadine treatment.
A54	Chaudhuri 2015				DA tapering/discontinuation due to side effects, mainly ICDs and hallucinations. 12 developed DAWS, 4 severe cases.
A55	Chen 2023	Most frequent ICD subtype: BE (48.7 % of ICD+ group).	With ICD: more often DA use, higher DA LEDD; no difference in sex, age, disease duration.<br>Higher DA LEDD predictor for any ICD and BE.	Metabolic factors: ICD+ patients more often had diabetes mellitus and higher HbA1c<br><br>Depression: ICD+ group showed greater depressive symptomatology<br><br>No significant group differences in cognitive score, BMI, dyslipidaemia, hypertension, smoking or alcohol history.	
A56	Chen 2024	5-year cumulative ICD incidence: 35.2 %	With ICD: younger, no difference in sex distribution, disease duration, DA use.	Carriers of APOE ε4: 44.3 % developed ICDs vs 32.3 % of non-carriers.<br><br>Anxiety (higher STAI) emerged as independent clinical risk factor.<br><br>BL sleepiness (ESS), depression (GDS) and probable RBD (RBDQ) were higher in patients who later developed ICDs	
A57	Chiang 2012	Prevalence of any ICB: 5.6 %.<br>– DDS 1.1 %; Punding 0.37 %; PG 1.49 %; CSB 2.99 %; BE 0.37 %; CB 0 %.	Male sex, higher total LEDD and LD LEDD associated with ICDs		
A58	Choi 2020		De novo compulsive behaviour in 3/38 after switch from DA to LD.	High BL OCD scores, higher anxiety and greater anger expression strongly predicted failure of DA switch off therapy for ICD.	40% showed increased ICDs after switch from DA to LD, 53% an improvement.<br><br>No effect on good/poor outcome by sex, age, disease duration, LD LEDD, DA LEDD, baseline ICD severity.

					Trend for higher total LEDD with poor outcome.
A59	Cilia 2014	66 % of DDS patients also had at least one ICD; 20 % had multiple ICDs	With DDS: no difference in age, disease duration, sex. Higher total LEDD, LD dose, DA dose.	Depression common pre-morbidly and at assessment.<br><br>Behavioural disinhibition, irritability, agitation frequent on NPI.<br><br>No strong association with dementia.<br><br>Substance-use history (nicotine, alcohol, cocaine, poly-drug) present in 17 % (personal) and 6 % (family) of DDS cases.	Symptom resolution in 14/35. Successful management: drastic LEDD reduction, some effect of advanced therapies (especially LCIG), clozapine treatment. Memantine in 1 patient. Monitoring by family in contrast to poor caregiving.<br><br>No/not much effect of optimising oral dopaminergic therapy (COMT, MAOB-I, tolcapone, LD fractioning) or antidepressants, psychological support, mood stabilisers.
A60	Contin 2023		With ICD: no difference in age, sex, disease duration, DA or LD treatment duration, DA type or dose, LD dose, total LEDD, other dopaminergics use.		
A61	Cooper 2009	Prevalence of CSB: 4.3%	CSB associated with younger onset age. No association with sex, DA use, total LD dose, disease duration.		
A62	Corbeil 2021	Prevalence of the problem PG in the cohort: 6.4%	Aripiprazole strong risk factor for PG, independent of confounders. No effect of treatment duration and dose.		
A63	Corvol 2018	Among patients without ICDs at BL the 5-year cumulative incidence of ICDs was 46.1%, DA ever-users: 51.5%.<br><br>ICD Prevalence increased over time: from 19.7% at BL to 32.8% after 5 years.	With BL ICD: younger, longer disease duration, less often white., higher DA use, higher DA LEDD,<br>No difference in PD onset age, sex, education, LD use, LD LEDD, selegiline use, amantadine use, COMT-I use.<br>ICD development during FU: more often male, younger, DA use, higher DA LEDD, cumulative DA LEDD, DA treatment duration.<br>Mild association with LD use, no effect of DA+LD.		30 with ICDs discontinued DA. ICDs resolved progressively, with 50% patients without ICDs 1 year after DA discontinuation.

			PPX and ROP equally strong risk factors. Lower risk with other DAs.		
A64	Crockford 2008	12-month prevalence in PD: problem PG 3.6 %, PG 5.7 % (total 9.3 %).			
A65	Cuciureanu 2019		Higher QUIP-RS for CSB in men. Higher QUIP-RS total associted with younger onset age, longer disease duration.		
A66	Cunnington 2012				DA tapered in 46/80 with side effects. ICD most common reason for therapy adaptation, all with ICD had DA tapered. DAWS more common with ICD.
A67	Dalrymple 2024	Higher depression, anxiety and apathy scores independently predicted worse QOL over time.<br><br>ICD presence was not associated with QOL change.			
A68	Dang 2011	PG: N=6, CB: N=4, CSB: N=3, Kleptomania: N=1<br><br>Consequences:<br>Financial loss AU $8 000 – $300 000, 3 marital separations, 1 criminal conviction, 2 suicide attempts.	ICD related to PPX, cabergoline and pergolide		
A69	Dawood 2020		Impulsive behaviour in 4/54 patients under aripiprazole depot and in 4/54 under aripiprazole oral treatment.		
A70	De Chazeron 2011	Prevalence (PD vs controls)<br>-PG: 0.87 % vs 0.87 %<br>- At-risk PG: 12.2 % vs 10.4 %<br>-CSB: 1.7 % vs 0 %		Alcohol: harmful use (AUDIT 8-10) lower in PD (2.6 % vs 10.6 %, $p<0.05$); alcohol dependence (AUDIT ≥ 11) similar (3 % vs 4 %).	
A71	De Chazeron 2019	BE disorder (BED) criteria: 3.9%<br>Food-addiction (FA) without BED: 39.2 %	With BE: more PPX use. No difference in LEDD, DA use.	Impulsivity and anxiety were the main correlates to FA	
A72	Delaney 2012	One of the key themes: ICBs as coping strategies: behaviours used to preserve identity, mitigate boredom/anxiety, or “live for the moment” given a feared future.			

		Conflicting views on causality: external attributions (dopamine replacement therapy) vs internal attributions (part of self, coping with PD).			
A73	De la Riva 2014		No total ICD increase over time in PD patients. More de novo ICDs in the group under dopaminergic treatment.		
A74	Delpont 2017		Female sex predictor for behavioural addictions.		
A75	De Sousa 2020	Any ICD 61 % vs 42 % in ctrls<br>CSB: 22 % vs 8 %<br>CB: 16 % vs 6 %<br>Punding: 19 % vs 6 %<br>Multiple ICDs: 33 % vs 15 %<br><br>Only 37 % knew DAs could cause ICDs<br><br>1/3 noticed symptoms fluctuated with dosing.	Male sex was risk factor for any ICD, CSB, PG. No sex difference was seen in BE, hobbyism, walkabout or DDS. CB: Younger age at diagnosis. No differences in DA treatment, pituitary tumor duration, DA type, weekly or cumulative cabergoline equivalent dose	Depression & anxiety scores higher in patients vs controls	Of 11 patients who interrupted DA treatment, 8 experienced resolution or improvement of ICD symptoms
A76	De Wit 2022	ICD frequency:<br>CSB: 17.7%<br>ICD:17.4%<br>DDS: 10.5%<br>PG: 9.3<br>Compulsive shopping: 8.6%	Disproportionality signal was mainly driven by dopaminergic drugs used in PD.<br>ICDs 6x more frequent in users of ADHD psychostimulants and 4x more frequent in users of dopaminergic antidepressants.<br>A high disproportionality signal for PPX, ROP, and rotigotine.		
A77	Diago 2017		ICDs more common under oral vs transdermal rotigotine DA treatment.	pRBD was significantly associated with ICDRB ($p = .001$).	
A78	Di Rosa 2022	Higher ICB scores in SWEDD vs PD and HC at 12 months ($p < 0.001$).		Anxiety (STAI-Y total) independently predicted higher QUIP scores and in SWEDD (drug-naïve) anxiety remained a significant predictor of ICBs.<br><br>Depression (GDS) and apathy (MDS-	

				UPDRS-I) were elevated in both patient groups vs controls but were not independent predictors of ICBs in multivariable models.<br><br>Cognition (MoCA) & executive / memory composites, motor severity and disease duration were not associated with ICB variability once anxiety was in the model.	
A79	Dogansen 2019	Any ICD prevalence 17 %<br>- CSB 9.7 %<br>- PG 2.9 %<br>- CB 4.5 %<br>- BE 6.1 %.<br>- Multiple ICDs 5.5 %.	Both any ICD and CSB more common in men, BE more common in women.		
A80	Drew 2020		With ICD: no difference in sex distribution, age, DA LEDD. Younger age at diagnosis, longer disease and symptom duration, higher LEDD.	78 % of PD + ICD met borderline/clinical apathy on LARS (esp. behavioural & emotional domains) despite high reward sensitivity.<br><br>Pupillary reward index could help identify patients at higher future ICD risk before clinical behaviours emerge.<br><br>No group differences in depression (BDI), anhedonia (SHAPS/TEPS) or global cognition (MoCA).	Current prevalence of ICDs less than anytime rates because in most cases patient's dopaminergic medication dose reduced to improve ICD symptoms. Of 11 only taking LD, nine had previously taken a DA before undergoing dopamine withdrawal to account for the emergence of impulsive behaviour.
A81	Driver-Dunckley 2007	7% noted a change in PG, 6% saying that increased urges and time spent PG specifically occurred after the use of DA<br><br>5% reported a high level of sexual desire, 4% associated it with the use of DA			
A82	Du 2023			Dopaminergic treatment elevated ICD risk predominantly through direct pathways, but part of its effect is channelled via worsening of RBD,	

				depression and anxiety, especially RBD.	
A83	Dubaz 2019				DA and amantadine prescriptions lower in 2017 vs 2010, higher MAOB-I use. No change in LD. No difference in DA in regression controlled for baseline variables -> no overall DA reduction despite increasing ICD awareness.
A84	El Otmani 2019	Any ICD prevalence 28 %.<br><br>CSB 8 %, CB 9.6 %, BE 7.2 %, PG 3.2 %, punding/hobbyism 11.1 %.<br><br>≥2 ICDs in 14 %; dopamine-dysregulation syndrome 10.4 %.	Risk factors any ICD: male sex, younger age. Disease duration not associated with ICDs.<br>BE, punding-hobbyism and CB mainly in women, while PG, CSB and DDS mainly in men.<br>With ICD: higher DA LEDD. No significant difference in DA type.		
A85	Erdal 2021		Similar OCD scores in patients and controls, no impact of PPX. More cleaning and doubting in RLS patients.		
A86	Erga 2017	ICB prevalence: 30.4 % in PD vs 11.9 % controls<br><br>Multiple ICBs: 8.8 % vs 1.3 %<br><br>Major ICDs in PD: BE 11 %, CSB 5.6 %, CB 4.8 %, PG 1.6 %.	No sex differences between different ICB types or ICB/no ICB. Patients with ICBs younger. No differences in MAO-B use, LD use or dose, DA LEDD, or total LEDD. Patients with ICBs more likely to use DA.<br>Highest ICB frequency in DA only, then DA+LD, then LD.	Compared to patients without ICBs, those with ICBs had higher MADRS total scores and MADRS subscores related to dysphoria and retardation.In addition, NPI items regarding depression, agitation, apathy, and irritability were more common in patients with than without ICBs	
A87	Erga 2020	Multiple ICBs were reported by 8.9% of patients and 1.3% of controls at initial assessment, 13.3% and 2.2% at 2-year FU, and 3.7% and 3.2% at 4-year FU, respectively.	ICB associated with lower age at each visit. DA use associated with ICB at BL and 2-year FU, but not at 4 years FU.	BL depressive symptoms (higher MADRS) were higher in ICB-positive patients	
A88	Erga 2020	Prevalence:<br>Any ICB reported: 45 % of cases.<br>– Self-reported by PD patients: 33 %.<br>– Only caregiver-reported: 12 %.<br><br>Impact on patients	Patients with self-reported ICBs younger, with higher total LEDD, more often under DA treatment. No effect of LD or DA dose.	ICB-positive patients more often showed depression, agitation, apathy and irritability	

		• PD patients who reported an ICB had significantly worse:<br>– Health-related quality of life<br>– Satisfaction with life<br><br>Impact on caregivers<br>• "Caregiver only" ICBs associated with marked caregiver distress<br>• Self-reported ICBs showed a smaller, non-significant rise in caregiver burden after adjustment.			
A89	Etminan 2017		Aripiprazole and PPX/ROP treatment risk factors for PG and other and ICDs.		
A90	Eusebio 2013		With pre-op DDS: younger, higher total LEDD, more often ICDs. Trend for more men. No difference in disease duration, LD treatment duration, DA dose.	Comorbidities in DDS:<br>high rates of psychiatric symptoms such as manic psychosis (61%), impulsive aggressiveness (61.1%), substance misuse (cannabis, alcohol), and smoking.<br><br>Greater non-motor fluctuations, especially 'on' euphoria/hypomania and 'off' dysphoria.	DDS resolved in 15/16 after surgery and ICDs in 24/25. No new DDS cases. No new ICD cases in pre DDS patients, 3 de novo ICDs in others.
A91	Evans 2004	Prevalence: punding identified in 17/123 (14 %) of all clinic patients and 34 % of the high-dose subgroup (17/50).	Punders: comparable age, sex distribution and disease duration, higher LEDD and more and higher rescue doses. More frequent apomorphine and cabergoline use, less frequent entacapone use. Stereotyped behaviours often influenced by subject’s previous occupation.<br>Higher punding severity correlated with younger age, higher total LEDD, and more frequent rescue doses.	Punders showed shorter nightly sleep compared to non-punders. Severity of punding correlated with sleep hours	
A92	Evans 2005		With DDS: younger onset age, longer disease duration, higher total LEDD. No difference in sex, DA use.<br>DDS risk factor: younger onset age.	Depressive symptoms strongly independently predicted DDS. All patients with DDS reported severe off-period dysphoria characterized by apathy, depression, anxiety.	

A93	Evans 2010		With DDS: younger, higher total/LD/DA LEDD. No difference in PD duration. DDS strongly associated with apomorphine infusion.		
A94	Evans 2014			Anxiety/depression may accompany the dopamine-driven form of ICD, but evidence is only suggestive.	
A95	Fabbri 2024	Prevalence of mild ICD among amantadine users (N=1049) 11.9%, moderate/severe ICD: 1.8%<br><br>DDS: mild: 1.1%, moderate/severe: 0.1%<br><br>Punding: mild: 1.5%, moderate/severe: 0.5%	Amantadine treatment associated with punding but not ICDs.		No significant improvement after amantadine introduction was observed for ICDs.
A96	Fan 2009	Prevalence of ICD among DA users: 6,3%<br><br>Nearly all ICD cases were taking piribedil (an oral D2/D3 agonist) CSB was the most common ICD behaviour identified in our study	Trend for lower mean ages and disease-onset ages of PD patients with ICD, trend for more male. Higher LEDD in ICD patients. No effect of amantadine. DAs a risk factor.		
A97	Fantini 2015	ICD/ICB prevalence: ≥ 1 ICD/ICB symptom in 53 % of PD-pRBD vs 28 % of PD-noRBD<br><br>Individual symptom rates higher in pRBD for: PG, CB, hobbyism, DDS.			
A98	Fantini 2018		With ICD: younger PD onset, longer disease duration, higher total LEDD, less ROP use.<br><br>No difference in age, sex, DA use, DA LEDD, PPX use, rotigotine use, piribedil use.	RBD prevalence: 85 % in ICD vs 53 % in controls (p = 0.002).<br><br>REM sleep without atonia (RSWA) higher in ICDs (42.5 % vs 28.6 %; p = 0.009).<br><br>Arousal index higher in ICDs (12.6 ± 10.6 vs 8.4 ± 4.8; p = 0.04).<br><br>Multivariate analysis: RBD independently associated with ICDs	

				(OR ≈ 4.6–4.9 after adjusting for age at onset, PD duration, severity, LEDD, DA-LEDD, antidepressants)	
A99	Fantini 2020	BL ICB symptoms: 20.7 %<br><br>Incident ICBs during FU: 39 %	With ICB: more DA use, more often male.	RBD, assessed by screening questionnaire, was not a clear independent risk factor for developing ICB symptoms in early PD.<br><br>Higher depressive symptoms were independent predictors of incident ICBs.	
A100	Faouzi 2022	BL ICD in PD with DA treatment:20%, lifetime ICD: 49%	Younger age, male sex, higher max dose and cumulative duration of DA treatment associated with ICDs		
A101	Farnikova 2012		With ICD: longer disease duration, higher total LEDD. No difference in sex, age, DA LEDD.		
A102	Fasano 2011				Intervention: 1) stepwise LD withdrawal, 2) D1 DA withdrawal, 3) introduction of COMT-I or other DAs, 4) amantadine introduction, 5) quetiapine/clozapine<br><br>Punding resolved in 8/10. amantadine introduction efficacious in 4/8.
A103	Fusaroli 2022	Prevalence pattern in FAERS: 74.5 % PG, 37 % CB, 34 % CSB, 10 % BE; 42 % had ≥ 2 ICD behaviours.<br><br>Co-reported impacts: economic problems 50 %, OCD symptoms 44 %, emotional disorders 34 %, suicidal/self-injury 29 %. Indicates substantial patient/family burden.	Vast majority of reports (94%) for aripiprazole, not brexpiprazole or cariprazine.<br>PG and CSB: More men.		
A104	Fusaroli 2023		Of all ICD reports, 30% for antipsychotics. 16% of all reports for DAs (60% PD, 34% RLS, 2% pituitary adenoma).<br>ICD reports more common in men and with younger age.		

			Antipsychotics: highest risk for aripiprazole, brexpiprazole, cariprazine. DAs: highest risk for pergolide, PPX, piribedil.		
A105	Fusaroli 2024		8% of all PPX reports were ICD-related, 5% of aripiprazole reports. ICD-AEs more common in men (PPX+aripiprazole) and younger patients (PPX).<br>ICD-AEs more common for PPX in PD than in RLS and more common for aripiprazole in mood disorders than psychotic disorders.		
A106	Galbiati 2015		Lower performance by RLS patients pre-treatment in stroop test and IGT. Improvement in both after 3 months PPX treatment.		
A107	García-Rubio 2021	Within the ICD group the most frequent behaviours were BE 59 %, CB 21 %, CSB 18 %, punding 5 % and PG 3 %<br><br>39 % had ≥2 ICDs.	With ICD: Longer PD duration, more often DA treatment, more often DBS, higher rasagiline doses, younger onset age in some ICDs.<br><br>CSB linked with male sex and DA treatment, BE with DA and LD treatment and DBS, CB with DA treatment.	The rigid-akinetic form of PD independently predicted compulsive shopping, whereas the tremor-dominant form was protective for that behaviour.<br><br>Non-motor symptoms (depression, anxiety, RBD, pain, hyposmia) were not different between groups in this cohort.	
A108	Garcia-Ruiz 2014	Prevalence: 39 % screened positive for at least one ICD on QUIP<br><br>Most common: hobbyism 45 (of 233), punding 29, CSB 28, CB 16, PG 9<br><br>41 % had ≥ 2 ICDs.	Associated with ICD: oral DA treatment (PPX and ROP), rasagiline treatment and younger age. No difference between immediate and extended release		
A109	Garrett 2019		Higher proportion of cases with history of RLS compared to controls, same for fibromyalgia. ICD cases: more DA use.		
A110	Gee 2015		4/16 had worse or stable post-op QUIP-RS.		Post-op QUIS-RS improvement in 12/16. Lower post-op total LEDD, no difference in total or DA LEDD

					between patients with improvement and not.
A111	Gendreau 2014		Only PG linked to DA presciptions but none of the other 3 ICDs.		
A112	Gescheidt 2016	ICD prevalence:<br><br>Any ICD symptom: 26.5 % of YOPD pts vs 10.5 % controls (trend, p = 0.07)<br><br>PG: 8.2 % vs 0 %<br>CSB: 10.2 % vs 0 %<br>CB 6 %<br>BE 6 % (no sig. diff.).<br><br>Two patients (4 %) showed ≥2 ICDs.	No association of ICD symptoms with PD duration, sex, LD use, total LEDD, DA+LD, amantadine use. Increased PG under DA treatment but no dose or treatment length effect.	Positive associations with ICDs: anxiety, higher somatization scores, smoking history and regular caffeine intake; personality styles: self-assertive/antisocial and reserved/schizoid<br><br>PG specifically linked to low conscientious/compulsive trait scores.<br><br><br>No link with depression scores, cognitive status, PD stage	
A113	Giladi 2007		27/193 patients with new onset ICD, none in control group. With ICD: younger onset age, higher apomorphine use. Trend for more often male and higher ROP use.<br>No difference in disease duration, LD use, LD treatment duration, DA use, DA treatment duration, amantadine use, selegiline use, entacapone use.		
A114	Giladi 2013		25/380 with ICD as AE, mostly under LD+rotigotine, not rotigotine monotherapy.		Rotigotine discontinuation after ICD-AE resolved ICDs in some cases.
A115	Goerlich-Dobre 2014	Prevalence (QUIP-RS): 57 % had ≥ 1 current ICD or related behaviour. 19.8 % met clinical diagnostic criteria (PG 4 %, CB 7.7 %, CSB 16.5 %, BE 19.8 %, hobbyism/punding 32 %).		Alexithymia, particularly difficulty recognizing and verbalising one's own emotions, was independently related to ICDs	
A116	Gómez 2017	Prevalence: 27.4 % screened positive for at least one ICD using QUIP-RS		Positive associations: RBD (OR ≈ 4.4),<br>consumption of tea (OR ≈ 3.1), mate (OR ≈ 3.7) and alcohol (OR ≈ 2.3).	

		ICD prevalence in DA-exposed patients 34.7 % vs 4.8 % in DA-naïve (RR ≈ 10.4)<br><br>Most frequent behaviour :hyperphagia/ BE 46 %, CSB 36 %, punding 20 %, PG 17 %, CB 14 %<br><br>23 % had ≥2 ICDs.			
A117	Grall-Bronnec 2022		With ICB: younger, younger at onset. No difference in sex, disease duration, treatment duration.<br>ICB risk factor: younger onset age.	Personal history of ICBs before PD onset strongly associated with later development of ICBs during PD.<br><br>Patients with ICBs showed higher impulsivity traits.	
A118	Grall-Bronnec 2024	28 % had ICBs at FU 74 % of ICB cases were receiving DA<br><br>higher PDQ-8 score was significantly associated with the presence of a current ICB. The highest score in PDQ scale was obtained for the Bodily Discomfort item, especially when an ICB was present.			
A119	Grosset 2006	Prevalence: 4.4 % of medicated PD patients met criteria for PG	With PG: younger, higher PPX dose. No difference in sex, DA type, ROP dose, LD dose, disease or DA treatment duration.		
A120	Gunduz 2024	Overall ICD prevalence: 7 %<br><br>Distribution (≥ 1 ICD, N patients):<br>-CB 52<br>-punding / hobbyism 50<br>-BE 34<br>-CSB 22<br>-PG 13<br>-DDS 3<br><br>59 patients (46 %) had ≥ 2 ICDs.	Patients with ICDs younger, more often male and more often in early PD stages, lower LD doses, higher PPX doses. DA use and DA+LD use more common with ICDs.<br>Effect of disease duration and DA+LD lost in regression analysis. PPX and ROP (both short- and long-acting), and DBS associated with ICDs.		ICDs generally improved after reduction of DA dosage or DA cessation
A121	Gungabissoon 2020		No ICDs in ROP ER group, less than 5 in DA IR group.		

A122	Hack<br>2014				Frequencies of centres reporting pre-op ICDs: 12%, post-op 5%. pre-op DDS: 21%, post-op 4%. Pre-op DAWS 5%, post-op 2.1%.<br><br>Pre-op strategies ICD: DA reduction/cessitation (83%/76%), psychiatric/psychological referrals (44%/20%), psychotropic medication.<br><br>Pre-op strategies DDS: LD reduction/cessitation (72%/37%), psychiatric/psychological referrals (37%/26%), psychotropic medication.<br><br>Psychotropic medication: ADs, mood stabilisers, quetiapine, clozapine, other APs.
A123	Hall<br>2023		Under DA with ICB: more often male. No difference in age, DA LEDD, DA type, total LEDD, DA treatment duration, disease duration.<br>Without DA with ICB: longer disease duration. Trend for higher total LEDD. No difference in sex, age.<br>ICB predictors: DA treatment duration		
A124	Hassan<br>2011	Compulsive behaviours (any): 22 %.<br><br>Pathological ICBs (harmful): 16 %.<br><br>When DA dose was ≥ therapeutic: pathological ICBs in 24 %; if dose reached ≥ target: 30 %<br><br>Sub-types among 69 cases (pathologic %): PG 36 % CSB 35 % compulsive shopping/spending 26 %	With CSB: more likely to be on "therapeutic" agonist dose and to be taking "target" dose. Dopamine agonist dosage appeared to be crucial.<br>LD add-on contributed to ICB development.With CSB: twice as likely to have undergone surgical PD treatment (DBS or pallidotomy).		DA reduced or stopped in 47 of the 69 compulsive behavior cases. Of 32 patients at FU, complete (24) or partial (5) resolution of compulsive behaviors. Remaining 5 patients had two or more behaviors with resolution of one behavior, while the other behavior continued despite dose reduction or cessation with FU pending.

		BE 17 %<br>compulsive hobbying 12 %<br>compulsive computer use 9 %.<br><br>42 % had ≥ 2 ICB types			
A125	Hauser<br>2014		Early PD under PPX ER: 13 ICD cases.<br>Advanced PD under PPX ER: 18 ICD cases		
A126	Heim<br>2016	Any ICD: 39.7%<br>Definitive ICD: 20.7%<br>ICD symptoms (n):<br>-BE 10<br>-punding / hobbyism 7<br>-CSB 8<br>-shopping 6<br>-PG 2<br>-hoarding 2<br>10 patients (43%) had ≥ 2 ICD symptoms.	Trend for higher total LEDD in ICD patients. No effect of age, sex, RLS severity, disease duration.	Augmentation was the independent predictor of ICD symptoms	
A127	Heim<br>2018		Higher reflection impulsivity under dopaminergic treatment, but no effect on reaction time	Elevated reflection impulsivity is present in RLS irrespective of medication, but dopaminergic therapy accentuates the tendency to decide with minimal evidence.	
A128	Heim<br>2021		With ICD: No difference in sex, age, disease duration, total LEDD.		
A129	Herath<br>2024	Prevalence (via QUIP):<br>Any ICD/CB: 16.7 %<br>Sub-types: BE 8.3 %; walkabout 10.9 %; CB 4.2 %; PG 1.6 %; CSB 1 %; hobbyism 6.3 %; punding 1 %; compulsive medication use 0.5 %.<br>47 % of ICD+ patients had ≥ 2 behaviours.		Independent predictors of having any ICD-CB:<br>greater functional disability,<br>personal history of psychiatric illness (depression/psychosis/bipolar),<br>family history of alcohol-use disorder.	
A130	Hernandez-Con<br>2023		Predictors of lower QUIP-RS in both groups: lower baseline QUIP-RS, lower time-varying LEDD or DA LEDD. No effect of age, sex, trend for longer disease duration.<br><br>4 patients in each group developed		No baseline differences in ICDs. Total LEDD decreased after DBS but not in controls. QUIP-RS decreased 6 months after DBS but increased slightly again until 12 months. No change in control group. No

			de novo ICD symptoms. All male, no obvious association with change in LEDD in DBS group.		difference in QUIP-RS between groups at any time point.
A131	Hidding 2017		QUIP-RS total and sub-scores: no change under STN and substantia nigra DBS, combined stimulation did neither aggravate nor improve ICD/ICB symptoms	Apathy: unchanged on group level<br><br>Depression & fatigue: no significant group effect.	QUIP-RS total and sub-scores: no statistically significant change under STN + substantia nigra DBS, combined stimulation did neither aggravate nor improve ICD/ICB symptoms
A132	Hinkle 2021	BL prevalence: punding 5.8 %, hobbyism 7.9 %, BE 10.3 %, CB 3.5 %, CSB 3.1 %, PG 0.8 %<br><br>Cumulative (5-yr) prevalence: punding 11 %, hobbyism 20 %.	High total LEDD but not DA LEDD predicted punding. No effect of either on hobbyism.	Worse attention (Letter-Number Sequencing, MoCA-attention sub-score) predicted higher odds of punding but not hobbyism.<br><br>ADL dysfunction (MDS-UPDRS II) independently predicted punding; motor score did not.	
A133	Hinojosa-Amaya 2020	ICD-risk overall 23.3%.	Non-significantly higher BIS-11 scores under DA treatment. Patients with BIS-11 scores of ≥60 points younger and received lower cumulative DA doses. No association with male sex, but trend for shorter treatment duration.	Clinically significant depression (moderate-severe) in about 23% of DA-treated patients; severe depression only in DA-treated.	
A134	Hollingworth 2015		ICB was 0% of all bromocriptine AEs, 14% for pergolide, 34% for cabergoline, 17% for PPX, 25% for ROP.		
A135	Holman 2009				Resolution of behaviors within 3–10 days after PPX discontinuation for 19 of 21 patients and for all within 3 months.
A136	Hu 2023	9.67% developed new ICD during years 1-4;<br><br>6.65% – ICD present at BL & persisted.	With ICD:No difference regarding age, sex.	Patients with ICDs had identifiable white-matter abnormalities at DTI; those relevant brain regions are involved in ICD and non-motor functions.<br><br>Patients with ICD have more frequent rapid eye movement sleep	

				behaviour disorders (RBDs) than those<br>without ICD<br><br>No differences in cognitive function	
A137	Hurt<br>2014	Prevalence of any ICB symptom (PG, CSB, CB or hobbyism): 17.8 %.<br><br>Hobbyism 11.8 %, shopping 6 %, PG 3.4 %, CSB 3 %.	With ICB: younger age, higher total LEDD, more DA use.<br>No difference in PD duration, sex, LD LEDD, DA LEDD.<br>PG more common in men.<br>Higher ICB rates on DA+LD than either in monotherapy.	No difference in ICB frequency between PIGD vs tremor-dominant/non-PIGD phenotypes.<br><br>Motor fluctuations associated with ICBs (39 % vs 26 %).<br><br>Anxious or anxious-depressed mood phenotypes strongly linked to ICBs.	
A138	Hälbig<br>2009		With DBS: no difference in OCD symptoms and ICD frequency, but higher impulsivity (BIS-11).		
A139	Ihle<br>2020	ICD Prevalence at BL: 19.4%.<br>Cumulative incidence over FU among patients without BL ICDs: 51.9%.<br>ICD prevalence increased by 8% per year (p < .001).	ICDs associated with DA use, higher DA dose, younger age at onset, and longer disease duration.		
A140	Imamura<br>2008		All PG patients men.<br>With PG: No differences in disease duration and dopaminergic therapy, with the exception of higher PPX doses in cases. Trend for younger onset in cases.		
A141	Inoue<br>2010		No episodes of ICDs were reported for any patient.		
A142	Irmen<br>2019			Change in risk-attitude was independent of depressive symptoms (BDI), self-reported trait impulsivity (BIS-11) or motor improvement magnitude.	
A143	Isaias<br>2008	ICD prevalence: 28% in PD vs 20% in controls<br>ICD types in PD (n): CB 5, CSB 2, intermittent explosive disorder 1, PG 1	Male sex associated with ICDs. No effect of total LEDD, PD duration or time under therapy.	Elevated impulsivity and depression in in-<br>dividuals with ICDs both PD and HCs	

		5 patients showed multiple ICDs			
A144	Izzo 2020	ICD prevalence: 19.6 % PD patients and 21.4 % HC met criteria for ≥ 1 ICD/related behaviour (in PD, n: PG 2, BE 3, punding 2, DDS 2, shopping + BE 1).		PD cohort: logistic regression showed higher delay-discounting "k" (preference for immediate reward) uniquely predicted ICD presence (OR≈19, p=.004) whereas reflection (CRT) and BIS-11 facets were non-significant.	
A145	Jaakkola 2014		With ICD: younger, younger at onset, more often male.<br>No difference in disease duration, total LEDD, LD LEDD, DA LEDD, MAOB-I LEDD, amantadine LEDD, LD use, DA use, MAOB-I use, amantadine use.	OC traits, depression and mild psychoticism were markedly higher in ICD+ PD patients. Patients with multiple ICDs exhibited more depressive and OCD symptoms than those with a single ICD.	
A146	Jakobsen 2016		1 case of BE in group without psychotic disorders, no other ICD-Aes		
A147	Janssen 2014		9/26 with impulsive behaviour. No differences between pre-operative characteristics of those with and without post-operative impulsivity, only trend for younger age.		
A148	Jeon 2020	3.0% developed ICD after initiation PPX/ROP.	Increased ICD risk when glutamatergic modulators, mainly amantadine, were added to PPX/ROP.	Comorbid psychiatric diagnoses did not explain the increased risk of ICDs	
A149	Jesús 2020	ICD prevalence: 12.7 % in PD vs 1.6 % in controls.<br><br>Compulsive behaviours (punding, hobbyism, walkabout, DDS) 7.2 % in PD vs 1.7 % in controls.	Higher presence of CSB in male patients with PD. No sex difference for ICDs in general.<br>PD patients with ICDs younger, younger at disease onset, longer PD duration.<br>After controlling for total LEDD, ICDs more frequent on DA treatment incl dose effect. No effect of LD or MAOB-I use, total LEDD or treatment duration with any agent.<br>DA type: Rotigotine with lowest risk, ROP highest , PPX in between, but no significant difference between	Patients with ICDs exhibited greater non-motor symptom burden (higher NMSS total; worse sleep, mood/cognition, urinary, sexual domains), more depression, higher FOG score, poorer PDSS sleep score, and worse quality of life (PDQ-39).	

			them.<br>QUIP-RS total score increased according to DA LEDD and total LEDD, but not with LD LEDD		
A150	Jesús 2021	Any ICD present in 25.1 %<br>Most frequent: BE (52 % of ICD cases).	More CSB in men, more CB in women. With ICD: younger, younger age at disease onset.<br>The use of DAs, DA doses related to ICDs. No effect of IR vs ER DAs, MAOB-I or LD use or any treatment duration.<br>PPX associated with the highest risk for ICDs, ROP trend for increased ICD risk, no differences for rotigotine.		
A151	Jeyadevan 2023	Increasing trait impulsivity is significantly associated with patient perceived health-related QOL in PD			
A152	Jiménez-Murcia 2012				Higher relapse risk in PG patients with PD after CBT (42% vs 24% without PD). Similar dropout and compliance in both groups. No difference in age and sex for relapses/non-relapses.
A153	Johnson 2023	Specific neuro-psychiatric symptoms—especially agitation, depression, apathy and irritability—meaningfully raise the burden carers feel.<br><br>Lack of an association between ICB severity, PD overall disease progression or ICB duration and carer burden			
A154	Joutsa 2012	At BL 42.5 % screened positive for any ICD/related behaviour with QUIP<br><br>FU: 38.7 % ICD+.	With ICD: more often male and higher DA LEDD. No differences between PPX and ROP.	Emergence of ICDs was accompanied by increase in depressive symptoms.	In 31 patients with resolved ICDs, 45.2% (14) patients were women compared to 17.1% (14 of 82) women in patients with stable ICDs.

		Dynamics: 8.1 % developed a new ICD, 11.5 % remitted, 30.4 % had persistent ICDs.		BL depression score did not predict future ICDs.	
A155	Joutsa 2012	Any ICD screen-positive: 34.8 %<br><br>multiple ICDs: 12.5 %<br><br>• PG : 7.0 % (problem PG 9.1 %)<br><br>CSB 22.8 %,BE 11.8 %, CB 10.1 %, PG 8.8 %.	Male sex risk factor for any ICD, PG, CSB. Younger age for any ICD. No LEDD effect.	Depression showed to be the strongest correlate with ICD. Patients with multiple ICDs had highest median BDI scores<br><br>Other associations: current alcohol use, current smoking, family history of PG	From those 13 who had changed medication because of ICDs, 11 still had a positive screen for ICDs: six for multiple ICDs, three for PG, and two for CSB.
A156	Kapsomenakis 2023	Overall frequency of ICD/ICB: 12.4 %<br><br>Most common single behaviours: BE 9 %; CB 3.9 %; PG 2.9 %; CSB 1.9 %.<br><br>70 % of ICD cases were receiving dopamine-agonists		Depression (29 of 111) and psychotic symptoms(39 of 111) frequently co-occurred with ICRBs<br><br>RBD and other parasomnias were highly prevalent in the ICRB group(65 of 111)<br><br>Physical comorbidities:<br><br>- Thyroid disorders in 26 of 111 patients<br>- Heart disease in 19 of 111 patients.<br>- Diabetes mellitus in 8 of 111 patients	
A157	Ke 2022		No difference in ICD incidence between any groups. Higher BIS-11 scores in untreated patients and controls.<br>Higher BIS-11 scores in women.<br>CSB correlated with female sex.<br>With ICD: no differences in bromocriptine treatment (duration, cumulative dose, daily dose).		
A158	Kehagia 2014		Atomoxetine added to dopaminergic therapy increased inhibition and reduced risk taking.		
A159	Kenangil 2010	ICD Prevalence: 5.9%<br><br>Punding: 57.5%	With ICD: No difference in age, onset age, disease duration, total LEDD, DA LEDD.		

		CSB/aggressive libido: 42.4%<br>BE: 27.2%<br>CB: 24.2%<br>Compulsive dopaminergic drug use: 21.1%<br>PG behaviour: 12.1%<br><br>Consequences:<br>behavioural symptoms led to insomnia, daytime sleepiness, occupational dysfunction, and serious financial/social consequences.<br>Case reports illustrated severe disruption of personal and family life (e.g., quitting jobs, selling homes, financial losses, marital strain).			
A160	Kim 2013	Any ICRB (ICD + RB + DDS): 15.5 %<br><br>ICD (PG, CSB, BE, compulsive shopping): 11.8 %.<br><br>CSB 7.1 %; BE 3.0 %; PG 1.3 %; shopping 1.0 %.<br><br>Repetitive behaviours (punding/hobbyism/walkabout): 6.7 %<br><br>Dopamine-dysregulation syndrome (DDS): 2.3 %<br><br>32.6 % of ICRB patients had ≥2 behaviour types<br><br>Impact on quality of life:<br>PDQ-39 scores were higher (worse HRQoL) in patients with any ICRB and rose further when ≥2 behaviours were present	ICD: younger, younger at PD onset, more often male, longer disease duration, higher LEDD, LD dose, DA dose.<br>Repetitive behaviour: longer disease duration, higher total LEDD and LD dose.<br>DDS: younger, lower onset age, higher DA dose. Abuse of both LD and DAs.		
A161	Kim 2013	Pre-DBS ICRB: 22.5 %	Pre OP ICBs compared with no pre OP ICBs: no difference in age, PD	Depression: Poor-outcome group (worsened/de-novo ICRB) had	Strong reduction of DA LEDD and total LEDD post OP.

		Post-DBS ICRB: 25.8 %<br><br>Improved/resolved in 65 %, 6 complete remissions.<br><br>De-novo ICRB in 10 %<br><br>Overall 32.6 % experienced ICRB pre- or post- DBS.	onset age, PD duration, total LEDD, DA LEDD.<br>Post OP ICBs compared with no post OP ICBs: no difference in age, PD onset age, PD duration, DA LEDD (pre, change), total LEDD (pre, change). Higher LEDD (post) and trend for higher DA LEDD (post).<br>De novo ICBs post OP in 9 patients (10%) + 3 aggravated pre OP ICB cases.<br>ICBs more common in men, especially CSB.	higher BL BDI and showed larger postoperative drop in BDI (improved mood) than good-outcome group.	Among pre OP ICB patients, ICB decreased in 13 patients (6 with complete resolution), aggravated in 3 patients, showed no change in 4<br><br>Good outcome (ICB improved or disappeared): no difference in age, PD onset age, PD duration, LEDD and DA LEDD (pre, change, post).
A162	Kim 2014			RBD prevalence: 61.2 %<br><br>Link between RBD and ICDs disappeared after controlling for age and disease duration.	
A163	Kim 2018		With BL ICD: younger onset age, younger, more DA use, higher total LEDD.<br><br>7 de novo ICDs at 1 year FU of which 6 resolved + 6 de novo ICDs at 7 years FU: No difference in sex, age, onset age, pre-op treatment, change in treatment, post-op treatment. Nominal greater reduction in DA LEDD and total LEDD.		Total LEDD reduction 7 years post-op. All 8 pre-op ICDs resolved at 7 years FU, most already after 1 year.
A164	Kobylecki 2020	Prevalence: 25 % screened positive for ≥ 1 ICB on QUIP	With ICB: higher LEDD	Participants with any ICB scored higher across all pain scales and were more likely to have high pain and neuropathic pain.<br><br>Neuropathic pain was associated with all ICB domains except PG and medication overuse, whereas there was no association between any ICB and nociceptive pain<br><br>The presence of any	

				ICB (OR = 1.5)<br>were associated with high pain.<br><br>CB behaviours associated with high pain (OR 4.0), higher total KPPS score (OR per point 1.02) and neuropathic pain (OR 2.1).<br><br>Eating behaviours linked to high pain (OR 2.8).<br><br>Punding linked to neuropathic pain (OR 2.0).	
A165	Koh<br>2022				8 weeks mindfulness: lower QUIP-RS score, trend for decreased BIS-11 score
A166	Kon<br>2018	ICB prevalence at BL: 21.3 %<br>New ICB developed in 12.6 %<br><br>Most frequent behaviours were hobbyism (43 %), BE (20 %), punding (20 %); no walkabout reported. Nearly half of ICB cases had >1 behaviour.	With ICB at BL vs not: more DA use, more pergolide use, more amantadine use. Trend for longer disease duration and more PPX IR use. No difference in sex, age, onset age, LD use, PPX ER use, ROP use, rotigotine use, selegiline use, entacapone use, total LEDD, LD LEDD, DA LEDD.<br>With ICB at BL and FU vs only at BL: younger, younger onset, more pergolide use. Trend for shorter disease duration, more DA use. No difference in sex, any medication at BL or medication development until FU.<br>With new ICB: more often male, more often new DA use, higher DA LEDD. More new rotigotine and entacapone treatment. Trend for younger onset age and higher total LEDD. No difference in age, disease duration, any other medication.	Dyskinesia significantly linked to incident ICB (50 % vs 17.5 % in non-developers, p = 0.006).<br><br>RBD, olfactory loss, cognition, mood (MMSE/UPDRS scores) not significantly different across ICB trajectories.	With new ICB: 64.3% had their DA discontinued, or dose reduced versus 15.5% without, PPX ER was discontinued or doses reduced more frequently.
A167	Kovács<br>2019		With ICD: higher DA LEDD, no difference in total or LD LEDD.	Depression (MADRS) and anxiety (PAS) had a moderate cor-relation with the ICDs (rho = 0 344	

				and 0.370, respectively, both p < 0 05)<br><br>No correlation between apathy (LARS) and ICDs.	
A168	Kraemmer 2016	ICD incidence:<br>19% of patients developed ICD behaviour over FU, per QUIP screening.	ICD predictors: DA use, no effect of age, other dopaminergics. Within patients on DA: male sex.		
A169	Krause 2022	Quality of life: PDQ-39 summary improved 30 % (p = 0.011). Motor UPDRS III improved 52 %.	No de-novo ICDs reported after DBS.		Lower QUIP-RS after DBS. Postoperative LEDD and DA reduction did not correlate with non-motor score changes.
A170	Krause 2022				3 week multidisciplinary rehabilitation program in day clinic improved QUIP-RS slightly. No correlation with age, disease duration, LEDD change.
A171	Kuhlman 2019	ICD symptoms judged infrequent and generally mild in this sample<br><br>No link between impulse-control symptoms (QUIP) or dopaminergic dose (LED) and health-related quality of life.			
A172	Kuzu 2016		Switch to PPX ER mostly without problems, CSB as AE in 1 patient.		
A173	Lamberti 2016		No difference between DBS candidates and controls in ICD frequency, with exception of more hobbyism and DDS in candidates.		
A174	Laurencin 2023				Stronger QUIP-RS reduction after 4 and 8 weeks under clonidine treatment than under placebo. Same number of patients affected in both groups though (4).
A175	Lawrence 2007		Punding in PD correlated with younger age, younger onset age, higher total LEDD, and non-LD LEDD, but not LD LEDD, longer disease duration. No sex difference.		

A176	Lee<br>2010	Overall ICD prevalence: 10.1 %<br><br>Punding 4.2 %, eating 3.4 %, sexual 2.8 %, CB 2.5 %, PG 1.3 %.<br><br>28.8 % of patients had ≥2 behaviours.	With ICB: more often male, younger, longer disease duration, younger onset age, longer dopaminergic treatment duration, higher LD and DA LEDD. Trend for more DA use.<br>PG and CSB more common in males.<br>DA LEDD but not LD LEDD predictor of ICDs. Vice versa for punding. No dose effect on BE.		
A177	Lee<br>2019			PD-ICD vs PD-non-ICD: higher K-NPI total/distress, anxiety (K-BAI), anger (STAXI), obsessive-compulsive (OCI-R).<br><br>No group difference on BIS-11 impulsivity score.	After 12 weeks intervention with LD substituting DAs, the mMIDI scores were significantly reduced in the ICD group. Strongest improvement in CSB. 4 patients showed paradoxical increases in mMIDI score after 12 weeks of intervention. Some problems with motor fluctuations and dyskinesia during intervention. 2 developed DAWS.
A178	Leplow<br>2023	Most common ICD behaviours: CSB 72 %, BE 53 %, CB 16 %, PG 6 %.<br><br>Patients with ICSs had worse quality-of-life subscores for cognition & communication (PDQ-39)	With ICD: more often male, younger, younger at onset. No difference in disease duration or any dopaminergic medication use or dose.	Patients with ICDs had greater depression severity (BDI-II, p = 0.04)<br><br>91 % of PD patients with ICD reported ≥ 1 criterion typical of substance-use disorder. Most frequent: positive expectancies, illusory control beliefs, positive mood after behaviour, persistence despite harm.	
A179	Leroi<br>2011	ICD group showed significantly worse HRQoL than behaviourally uncomplicated PD controls		Depression levels were high in ICD group	
A180	Leroi<br>2012	Carer burden is elevated when PD patients exhibit ICDs		Depression & anxiety were both significantly higher in ICD group.<br>In regression, depression was a key contributor to burden in the ICD group.<br><br>Motor complications: UPDRS-IV scores higher in ICD vs controls.	

A181	Leroi 2012		With ICD vs controls: younger, longer disease duration, higher total LEDD. No difference in sex, onset age, DA use, PPX use, ROP use, DA LEDD.	ICDs strongly associated with higher anxiety (HADS-A) and elevated NPI domains (sleep, appetite, irritability).	
A182	Leroi 2013			Impulsivity: ICD group had higher BIS-11 total scores than both PD-controls and HCs. When ON medication, ICD patients chose significantly more immediate/smaller rewards (greater impulsive choice) than non-ICD PD. This difference disappeared OFF medication.<br><br>Apathy: lower AES-C scores in ICD vs PD-controls (less apathy).<br><br>Mood disorders: Current DSM-IV depression similar (34 % ICD vs 18 % controls).	
A183	Lertxundi 2018		Strong association between ICD-AEs and aripiprazole, especially PG and CSB. Stronger association with depot vs oral.		
A184	Lhommée 2012	At BL:<br>Dopamine-dysregulation syndrome 6 %.<br><br>Behavioural addictions/ICDs 27 %<br><br>Isolated compulsive dopaminergic medication use 14 %.	1 de novo DDS patient.		Reduction of total LEDD, DA LEDD and LD LEDD post-op. All ICDs reduced post-op, especially hobbyism and DDS.
A185	Liang 2023	DDS incidence:<br>2.2% at BL.<br>Cumulative incidence over 5 years: 2.8%, 6.4%, 10.8%, 15.5%, and 18.7%.	With baseline DDS: No difference in age, sex, onset age, disease duration.<br>Highest risk to develop DDS under DAs, lowest under LD.	ICD-related behaviours (e.g., compulsive shopping, CSB, eating) were strongly predictive of DDS.<br>Anxiety was a significant independent risk factor.<br>Depression and RBD were more common in DDS cases at BL.	

A186	Lim 2011	Prevalence (QUIP-positive for ≥1 behaviour)<br>- 35 % by patient + informant report, 24.6 % by patient alone, 27.4 % by informant alone.<br>- Most frequent domains: BE (13.5 %), CSB (13.0 %), punding/hobbyism (20 %); PG (3.5 %) & compulsive medication use (4.5 %) were least common.	With ICB: more often male, younger onset age, longer disease duration, higher total LEDD, DA use, higher DA LEDD, amantadine use, higher amantadine dose, PPX use, ROP use. No difference in piribedil use. ICB predictors: male sex, longer disease duration.	No association with cognition (MoCA) or apathy (Starkstein scale)	
A187	Limotai 2012		With DAWS: higher total LEDD, LD LEDD, DA LEDD. Trend for longer disease duration. No difference in sex, age, onset age.<br>With DDS: younger onset age, higher total LEDD, LD LEDD, DA LEDD. No difference in sex, age, disease duration.<br>With ICD: more often male, younger, younger onset age, higher total LEDD, LD LEDD, DA LEDD. No difference in disease duration.		
A188	Lipford 2012	ICDs: 10 %			ICDs were treated by the discontinuation of PPX (n = 1), or dose reduction (n = 4).
A189	Liu 2021	Impulsivity after surgery remained high and was an independent predictor of poorer QOL	LEDD deceased at every FU compared to pre-surgery. No changes in BIS-11 score after DBS at any point.		
A190	Lopiano 2019		No de novo ICDs reported as severe AEs.		Improvement in QUIP-RS after initiation of LCIG.
A191	Lubomski 2021	ICDs were present in 19.4 % (QUIP).<br><br>Presence of an ICD and RBD were predictors of poorer health-related QOL (after adjusting for age, sex and disease duration)			
A192	Lulé 2012		Trend for worse IGT performance under higher doses in all 30. No effect of on vs off in DBS patients on IGT. PD-DA patients chose less		

			advantageous cards compared to PD-DBS off patients.		
A193	Lyons 2010				Orally disintegrating selegiline added to allow DA decrease. ICDs reduced in 13/25 and resolved in 8/25.
A194	Maggi 2024	ICD / ICB prevalence (sub-syndromal ≥1 on QUIP-RS): 54 % of patients. 22 % reported ≥2 ICD/ICB domains.<br><br>Most frequent domains: hobbyism/punding 21 %, DDS 20 %, BE 16 %, CSB 15 %, PG 13 %, CB 10 %.<br><br>Full-threshold ICDs (cut-off positive) in 11 % (BE 9 %, PG 6 %, CSB 3 %, CB 2 %).	No association of QUIP-RS with age, sex, disease duration, total LEDD, LD dose, DA dose, COMT-I dose, MAOB-I dose.	QUIP-RS scores were not correlated with depression (BDI-II) nor apathy (DAS)	
A195	Maia 2003	Frequency of OCD/OCS & related disorders (tic disorders, trichotillomania, body-dysmorphic disorder) did not differ between PD and controls:<br><br>OCS / sub-threshold / definite OCD in PD: 17 % vs 12 % controls (non signif).<br><br>Definite OCD: 5 % PD vs 3 % controls (non signif).<br><br>Severity of OCS (Y-BOCS scores) similar in both groups (≈ 17 vs 14, non signif).	No correlation of compulsive behaviour with disease duration, onset age, LD therapy.	CS, particularly symmetry obsessions & ordering/arranging and contamination/cleaning were significantly more common in PD patients whose motor symptoms predominated on the left side.	
A196	Mamikonyan 2008				At BL all 15 on DA, 7 discontinued at FU (all in full remission), 5 reduced dosage (all in full or partial remission). Additional help through support group and antidepressants. DA LEDD reduced by -52.6% on

					average, LD LEDD increased, no change in total LEDD.
A197	Marder 2007		ICD-AEs less common than in 5% or not present		
A198	Maréchal 2019	QUIP screening positive for ≥1 ICB in 37/88 (41%)<br><br>Positive for ≥1 classical ICD in 15/88 (17%): PG 1%, CSB 9%, CB 6%, BE 9%.<br><br>Positive for punding/hobbyism/walkabout in 30/88 (34%); DDS positive in 1%.<br><br>Semi-structured diagnostic interview completed in 22/37 QUIP-positive pts → ICB confirmed in 6 → Positive-predictive value (PPV) 27% (ICD PPV 33%).	With ICB: younger onset age. No difference in age, sex, disease duration, LD use, DA use, total LEDD.	History of mood disorder (24%), substance abuse (15%) captured, but no differences between QUIP-positive vs QUIP-negative	
A199	Marín-Lahoz 2018	ICD prevalence: 31 % incident cases identified by structured clinical interview (most frequent: hobbyism/punding, BE; no PG).	With ICD: younger, younger onset age, more DA use. Trend for higher total LEDD, significant after controlling for motor symptom severity. No difference in PD duration, sex, DA LEDD among DA users, LD LEDD, MAOB-I use, amantadine use.	Impulsivity (BIS-11) did not increase ICD occurrence, whereas pre-existing impulsivity amplifies the severity once an ICD has developed.<br><br>Current alcohol use and current smoking significantly associated with ICDs; earlier age of smoking initiation predicted higher risk and greater ICD severity.<br><br>No relation found with legal caffeine use, illegal drug history or family history of substance abuse.	
A200	Marín-Lahoz 2019	ICD incidence: 35 % during FU—50 % of depressed vs 34.7 % of non-depressed.	DA use and DA LEDD associated with ICD development independent of depression.	BL depression associated with higher risk of developing ICD	
A201	Marín-Lahoz 2022	19 % developed an ICD during the 30-month observation period.	With ICD: No difference in sex, age, onset age, disease duration, total LEDD, DA LEDD, cumulative total LEDD, cumulative DA LEDD, DA use.	BL assessments of impulsivity (BIS-11, delay discounting, risk-taking tasks), anxiety, depression, apathy, RBD, cognition (PD-CRS) showed no significant differences between	

				groups and none predicted ICD onset in survival models.	
A202	Marković 2020	Prevalence / dynamics:<br><br>ICB present at BL in 21/106 PD (19.8 %) vs 10.4 % in controls.<br><br>Cumulative prevalence rose significantly: 20.7 % (Y1) → 29.2 % (Y5); increase became significant after 2 years.<br><br>Multiple ICBs: 4.7 % at BL, 61.9 % at Y5.<br><br>Resolution in 30 % (most often BE); hobbyism sometimes evolved into punding.<br><br>Individual behaviours (BL → Y5):<br>– Hobbyism 11 % → 8 % (stable)<br>– Punding 2 % → 14 % (↑, p = 0.001)<br>– BE 2 % → 12 % (↑, p = 0.028)<br>– CSB 2 % → 12 % (↑, p = 0.001)<br>– CB 1 % → 6 % (↑, p = 0.024)<br>– PG remained rare (≈2 %).	PD with baseline ICB: more often male, more DA use. No difference in age, onset age, disease duration, total LEDD, DA LEDD, treatment duration.<br><br>ICB predictor during BL and FU: DA use at baseline. For CSB: male sex.	CB was associated with BL depression score.<br><br>No BL cognitive differences between PD-ICB and PD-noICB, but trends towards greater decline in attention/fluency in ICB group.<br><br>Psychiatric scores (depression, anxiety, apathy) worsened over time in both groups but did not differentiate incident ICBs.	
A203	Marques 2018	Prevalence / risk of ICDs (diagnosed by standard criteria & Ardouin Scale):<br>-any ICD/related behaviour: 50 % in PD-RLS vs 26 % in PD-noRLS (p = 0.03).<br>-BE: 30 % vs 10 % (p = 0.02).<br>- other individual ICDs (PG, shopping, CSB) not significantly different	RLS as independent, strong risk factor for ICDs. No effect of sex, age, treatment duration, total LEDD.	PD patients with RLS: more frequent ICD-RB, more BE behaviour. Trend for higher level of risk-taking behaviour (p=0.06) and more hobbyism (p=0.07).<br>More hyper-dopaminergic behaviours – hypomanic mood (13 % vs 0 %, p = 0.02) & excess motivation (27 % vs 4 %, p = 0.005).<br>No differences in hypo-dopaminergic items (depression, apathy, anxiety).	
A204	Marques 2021		With ICD: longer disease duration, longer treatment duration, higher LEDD. No difference in sex, age, DA LEDD, DA use.	RLS prevalence: 36 % overall (30 % non-ICD vs 47 % ICD; non signif diff)<br><br>Higher rates of anxiety, irritability	

				and antidepressant use in ICD group.<br><br>Excessive daytime sleepiness (Epworth) greater in ICD group.<br><br>No differences between groups in polysomnographic sleep measures, AHI, PLMS, or RBD prevalence.	
A205	Marques 2022			PD patients with RBD have a markedly higher prevalence of active ICDs (55 % of PD-RBD screened positive on QUIP vs 5 % in PD-nRBD).	
A206	Martinez-Martin 2020	Prevalence of any ICD: PD-D 23.5 % vs PD-ND 13.3 % (P = 0.02).<br><br>Hobbyism/Punding: 32.9 % vs 10.6 % (P < 0.001).<br><br>Dopaminergic-medication abuse / DDS: 8.2 % vs 3.2 % (P = 0.03).	With ICB: more DA use. ICD predictors: younger age, male sex. Punding/hobbyism predictor: younger age.	Dementia independently predicted hobbyism-punding	
A207	Martinez-Nunez 2025			In de-novo PD the presence of probable RBD at diagnosis is an independent early marker for later ICD (early-RBD group showed higher QUIP scores at every FU interval. Prevalence of ICDs 55 % in RBD vs 5 % in non-RBD).	
A208	Martini 2018			Patients with ICDs showed significantly higher depression and anxiety scores vs. HCs (HADS total and sub-scales).<br><br>Day-time sleepiness (ESS) was higher in patients with ICD vs. controls.<br><br>No between-group differences in apathy (Starkstein), impulsivity self-report (BIS-11) or cognitive stage scores.	

A209	Martini 2019	ICD/ICB present in 55 % of PD-normal cognition, 50 % of PD-mild cognitive impairment, 42 % of PD-dementia– differences not statistically significant.<br><br>Quality-of-life (PDQ-8) worse in ICD+ in PD-normal cognition & PD-mild cognitive impairment.		Depression (BDI-II) and anxiety (STAI) showed no between-group difference (ICD+ vs ICD–) within cognitive strata except higher BDI in PD-dementia overall.	
A210	Martinkova 2011		2 with an ICD, both male.		
A211	McKeown 2020	ICBs placed profound psychological, relational and economic strain on carers (low recognition of ICBs; lack of prior warning from clinicians; difficulty linking behaviours to DAs)		Carers noted partners' lack of insight and co-existing disinhibition, aggression and secretiveness.	
A212	Meira 2021	Prevalence of ICDs after continuous sub-cutaneous apomorphine infusion:<br>At M3: mild ICDs: 23.1%, moderate: 6.2%, marked: 6.2%. At M24: mild ICDs: 8.6%, moderate: 10.3%, marked: 3.4%	3/38 had to stop apomorphine due to ICD.<br>8/25 with previous ICD and completed treatment experienced worsening under apomorphine.	Depression & anxiety BL scores were not associated with ICD outcome	25 with previous ICD who completed treatment: 6 in remission, 5 with stable ICD, 6 with ICD recurrance.
A213	Meng 2023			ICDs were not associated with global cognitive decline	
A214	Merola 2017	BL ICB prevalence 17.3 %; post-DBS trend down to 12.7 % ($p = 0.095$).<br><br>Significant reductions in CSB (12 → 8 %), PG (10.7 → 5.3 %) and DDS (4.7 → 0 %) ($p < 0.05$).<br><br>Of 26 BL ICB cases: 69 % remitted, 31 % persisted.<br><br>• 7.3 % developed new-onset ICB	11/150 with de novo ICB post-op. With pre-op ICB: more often male, younger. No difference in onset age, disease duration, total LEDD, DA LEDD, DA use. With new-onset ICB: younger, more often female.	Persistent ICB was correlated with OCD personality traits.<br><br>New-onset ICB were correlated with borderline/schizoid/schizotypal traits.<br><br>Mood (BDI) trended worse in persistent & new-onset ICB groups. No major cognitive differences noted.	Total LEDD reduced post-op in ICB outcome groups. Trend for ICB prevalence reduction post-op. 70% with pre-op ICB in remission, 30% with persistant ICB.
A215	Miyasaki 2007	Punding prevalence 1.4 %			Punding reduced by reduction of LEDD in 3/4, 1 refused adjustment.
A216	Mohammad 2021		65 patients with behavioural side effects, of which 50% ICDs		DA tapering more common with behavioural side effects than without, but with similar speed.

					DAWS not correlated with occurrence of behavioural side effects.
A217	Mohammad 2024	ICD prevalence: 30 % vs 6.7 % in controls<br><br>ICD subtypes in cases: CSB 4 (all men), BE 4, CB 2, repetitive cleaning 2, repetitive door-closing 1; 3/9 had ≥2 ICDs.<br><br>No pathologic PG (illegal in Iraq).	With ICD: No difference in disease duration, age, onset age, sex, current cabergoline dose. Trend for higher maximal cabergoline dose.	ICDs often associated with irritability	
A218	Monaco 2018	44.7% had at least ICDs vs 25.2% in control group.<br>CSB and CB were significantly more common in the PD group compared to controls. The prevalence of other ICB was 42.5% in PD patients and 38.9% in the control group	PD with ICD: younger. Trend for more DA use. No difference in age, sex, LD use, LD+DA use, total LEDD, other dopaminergics use. PD with compulsive behaviour: Trend for more LD+DA use. No difference in age, sex, DA use, LD use, total LEDD.	Among PD patients with ICDs there was numerically higher use of antidepressants (23.5%)	
A219	Moore 2014		DA-related ICD-AEs in PD (62%), RLS (24%), hyperprolactinemia (4%), others. All DAs and aripiprazole associated with ICD-AEs, especially PPX and ROP. Even weakly associated with LD, antidepressant and antipsychotic use.		
A220	Morgante 2016	Overall prevalence of "≥1 ICB" similar between groups.<br><br>Specific ICBs more frequent in parkin-PD: CB, BE, punding/hobbyism<br><br>Total QUIP-RS score higher in parkin-PD.	With ICB: no difference in age, disease duration, age at onset, total LEDD, DA LEDD. No correlation between QUIP-RS and onset age, disease duration, total LEDD, DA LEDD.	Parkin genotype associated with smoking status.<br><br>No difference between groups in UPDRS-III, depression, psychosis, dementia, RBD	
A221	Morgante 2021		ICBs more frequent in the younger group, also trend for higher QUIP-RS. ICB associated with higher DA LEDD. Higher QUIP-RS score correlated with younger age, younger onset age.		

A222	Morrow 2023	ICD prevalence: 20 % had QUIP score > 1.	With ICD: Higher total LEDD. No difference in age, sex, disease duration, DA LEDD, DA use.	Participants with an ICD had higher anxiety and depressive scores in "on" and "off" states of medication and larger changes in depressive symptoms from OFF to ON states compared to those without an ICD.<br><br>Current antidepressant use associated with greater odds of ICD.<br><br>Alcohol-use disorder history more common with ICD	
A223	Morrow 2024	Cumulative ICD incidence: 27 % developed ICD during study; BL prevalence 10 %.	Increased ICD risk associated with male sex, younger age, DA use, MAOB-I use, higher total LEDD, longer disease duration. No effect of amantadine use.	Greater ICD risk in participants exposed to any serotonergic antidepressant vs unexposed	
A224	Moum 2012		With ICD and/or DDS: no difference in onset age, age, sex, pre-op DA use. Higher pre-op total LEDD in the one ICD+DDS patient. 2/7 pre-op ICD patients developed post-op DDS (STN), no other the novo DDS cases. No change in DA use, DA LEDD, total LEDD post- vs pre-op in 17/17 de novo ICD cases post-op. 11/17 after unilateral stimulation, 7 in GPi, 4 in STN. Resolved in 4/5 by additional bilateral stimulation. 6/17 after bilateral stimulation, 2 in GPi, 4 in STN.		No change in DA dose post- vs pre-op in ICD patients. 1/7 pre-op ICDs resolved after unilateral STN DBS, 2 additional patients in remission after bilateral DBS, one in STN, one in GPi. No significant difference between STN and GPi DBS.
A225	Nadeau 2018	PG patterns (past 6 months):<br>-predominant games: lotteries 93%, casino slot machines 33%, cards for money 13%.<br><br>Motivations: pleasure (23%), ‘‘hope to win money for PD cure or assistance’’ (31%), social outing (31%), "voluntary tax" (lottery).		Comorbidities: 1 CSB 1 CB; no alcohol/drug/BE disorders.	

		77% began PG before PD diagnosis; 3 started after.			
A226	Nardone 2014				Transient decrease in punding after low frequency repetitive transcranial magnetic stimulation (LF rTMS) over the right dorsolateral prefrontal cortex vs sham, no effect at last FU.<br><br>Transient decrease in punding after LF rTMS over the left DLPFC vs sham, no effect at second and last FU.
A227	Nissen 2012		ICDs most common reason to stop DA treatment (in 6%).		
A228	Okai 2013				Improvement in general symptom severity (CGI) under CBT, ICB frequency and impact was significantly reduced
A229	Okai 2015				CBT administered after changes in medication were unsuccessful in all patients. Improvement of general symptom severity and neuropsychiatric symptoms after CBT.<br><br>Associated with general improvement: fewer ICBs, DA use (!), lower LEDD. No age effect.<br><br>Associated with NPI improvement: lower LEDD, no effect of ICB number or DA use.
A230	Olanow 2020		No ICDs as AE. No difference in QUIP-RS between apomorphine and placebo.		
A231	Olley 2015		With PG: No difference in age, onset age, sex, disease duration. PG severity increase associated with dopaminergics.	Certain UPPS impulsivity traits (positive & negative urgency, lack of planning/perseverance) and higher anxiety scores were significantly more common in problem PG group compared to controls	Main strategies against PG: change in dose, medication class or specific drug. Remission in 6/20, reduced PG in 10/20. Also remission/PG reduction without medication changes.

					Other strategies: Therapy/counselling, GA meetings, self-exclusion.
A232	Ondo 2008	Prevalence of any DA-associated impulsivity: 19.7 %<br><br>Manifestations (non-exclusive, in n): PG 30; excessive spending 26; increased sexual activity 11; 1 case of compulsive travel (11 patients had ≥2 behaviours).<br><br>Only 18.6 % considered the behaviour harmful.<br><br>PG (DSM-IV criteria): 2.3 %; all PD; 6 on PPX, 1 on pergolide). All lost ≥US$10,000.	Younger age, higher DA LEDD and PD diagnosis risk factors for increased impulsive symptoms. PD lost significance after DA dose correction. No effect of disease duration, LD use, LD LEDD, sex, DA treatment duration.		3 with severe PG switched from PPX to ROP without PG recurrence in 2, but with in 1.
A233	O'Sullivan 2010	Prevalence of excessive hoarding (SIR total > 40):<br>• PD+ICB: 27.8 %<br>• PD–ICB: 3.5 %<br>• HC: 6 %<br><br>ICBs present in the PD + ICB cohort (overlap allowed): punding 20, BE 14, CSB 12, PG 11, CB 11, DDS 11.	PD with ICB: younger, younger onset age. No difference in PD duration, sex, LD LEDD, DA LEDD, LD+DA LEDD. PD with hoarding: younger onset age, longer PD duration. No difference in sex, age, total LEDD.	Hoarding severity did not relate to depression or anxiety scores (HADS).	
A234	O'Sullivan 2011		PD with ICB: Younger age, younger onset age, higher LD+DA LEDD. No difference in sex, PD duration, LD LEDD, DA LEDD.	PD + ICB reported significantly poorer global sleep than PD – ICB and controls.<br><br>PD + ICB exhibited higher anxiety and depression plus more manic-like symptoms (MDQ). Mood scores correlated with poorer sleep.<br>RBD: similar prevalence in PD + ICB vs PD – ICB.	
A235	Ozdeniz Varan 2023		No difference in BIS-11, MIDI, or IGT between any groups.		
A236	Ozer 2024		Higher impulsivity in RLS patients than in controls.		

A237	Ozkaya 2020		ICDs under DA treatment: 2/40 with acromegaly, 3/40 with prolactinoma (no difference in prevalence, despite higher DA doses in acromegaly). 4/5 female. No association of BIS-11 with DA dose, cumulative dose or DA treatment duration.		2/3 patients with prolactinoma and ICD underwent surgery and the other patient's cabergoline dose was reduced. Cabergoline dis-continued in the 2 patients with acromegaly. All patients' ICD symptoms resolved after intervention.
A238	Paliwal 2019		Higher QUIP-RS correlated with higher total LEDD. No correlation between BIS-11 and total LEDD.		Decrease in total LEDD and impulsivity post-op. Trend for lower QUIP-RS post-op.
A239	Pallanti 2010		With punding: No difference in age, disease duration, total LEDD, post-op LEDD decrease.		
A240	Papay 2011	Prevalence by diagnostic interview: overall ICDs in 12.7%: CSB 7.0%, BE 3.2%, CB 2.8%, PG 1.4%.<br><br>Validity of the QUIP (patient vs. informant):<br>-Sensitivity 100 % for both patient- and informant-rated QUIP.<br>-Specificity ≈ 75 % for both versions.<br>-NPV 100 % (a negative QUIP from either source ruled-out an ICD).<br>-PPV ≈ 37 % (many positive screens reflected sub-syndromal symptoms).<br><br>Patient–informant agreement:<br>-any ICD behaviours: moderate agreement (κ = 0.41).<br>-agreement highest for PG (κ = 0.55), lower for sex, CB and eating.			
A241	Papay 2014				No superiority of naltrexone compared to placebo in general symptom improvement, but greated QUIP-RS decrease.
A242	Paul 2020	Prevalence: ≥ 1 ICD-RB in 41.2%; ≥ 2 in 15.7%.	With ICD: higher total LEDD, higher total DA LEDD, trend for longer	Substance/alcohol use correlated with ICD.	

		Any ICD (PG, CSB, CB, BE): 16.7%<br><br>Most frequent behaviours: compulsive medication use & punding (each 12.8%), hobbyism 7.8%, BE 6.9%, PG 3.9%, CSB & CB 2.9% each.<br><br>2.9% had trichotillomania<br><br>Gender: overall ICD-RBs slightly more frequent in women (42%) than men (39%). Punding & compulsive medication use more common in women; CSB & walkabout only in men.<br><br>Quality of life (PDQ-39) worse in ICD-RB group.	disease duration, younger. No difference in sex distribution.		
A243	Paul 2023	Overall ICD-RB prevalence: 53.6% RLS prevalence: 18.9%. Co-occurrence: 12.6% had both RLS and ≥1 ICD-RB; i.e., 66.7% of those with RLS also had an ICD-RB.<br><br>Distribution of ICD-RBs in total cohort:<br>Compulsive medication use 47.4%.<br>BE 29.4%.<br>CB 17.6%.<br>PG 11.7%.<br>CSB 3.9%.<br>Other repetitive/compulsive behaviours 29.8% (e.g., hand washing, internet use, etc.).<br><br>In PD patients with RLS, PG (27.8%) and BE (44.4%) were significantly more common than in PD patients without RLS (PG 1.9%; eating 13.7%).		No significant association between addictive history and RLS-ICD co-occurrence was found	

A244	Perez-Lloret 2012	Prevalence of any ICD symptom 25 % in PD vs 0 % in controls.<br><br>CSB 10%<br>BE 14 %<br>CB 6 %<br>PG 3 %.	With ICD: younger, DA use, MAOB-I use, higher DA dose, higher LEDD (categorical). No difference in disease duration, LD use. Trend for more males. Bromocriptine, ROP, PPX individual risk factors.		
A245	Pettorruso 2016	≥1 ICD present in 40 % of punders vs 17 % controls (p<0.01).<br><br>PG 20 % vs 2 % (p<0.0001).<br><br>CSB 36 % vs 12 % (p<0.01).<br><br>Hedonistic homeostatic dysregulation 8 % vs 0 % (p<0.01).	With punding: more DA use, higher DA LEDD. No difference in age, sex, disease duration, education, onset age, LD use, LD LEDD, total LEDD.	Psychiatric comorbidity (punding): higher SCOPA-PC psychosis/compulsivity subscores; more bipolar-spectrum history (8 % vs 0 %); greater impulsivity (BIS-11) and anhedonia (SHAPS).<br><br>RBD more frequent in punders (56 % vs 37 %; p<0.05).<br>No significant difference in depression/anxiety scores.	
A246	Phu 2014	5 % had at least one impulse-control or related disorder.<br><br>ICDR independently predicted worse QoL, particularly emotional-well-being domain.<br><br>ICDR also independently associated with greater ADL disability (UPDRS-ADL) after adjusting for age, sex, MDD and PD duration.	With ICB: no difference in age, sex, disease duration, total LEDD, DA use.		
A247	Picillo 2021		No difference in post-op QUIP-RS between LD and DA group. 2 de novo ICD cases in DA group, none in LD group.		
A248	Pineau 2016			ICD-PD group had greater dysphoria (MADRS dysphoria factor) and apathy (Starkstein scale).<br><br>No correlation reported between executive scores and ICD severity; impulsivity (BIS-11) elevated in ICD group.	

A249	Poletti 2013	8.1 % had ≥1 ICD.<br><br>PG 3.2 %<br>CSB 3.0 %<br>BE 2.4 %<br>CB 1.0 %<br>Punding 0.2 %<br>DDS 0.2 %<br><br>Prevalence in cognitively intact vs demented: 9.6 % vs 3.8 %.	With ICD: more often male, younger, younger at diagnosis, more DA use, more amantadine use. No difference in disease duration, LD use. DA use itself associated with male sex, younger age, younger age at diagnosis. ICD predictors: male sex, younger age, DA use, LD use, less amantadine use. No difference between PPX and ROP.		
A250	Pondal 2013		13/84 with DAWS, all of them ICD patients, much higher frequency than in non-DAWS patients. DAWS linked to DDS in some cases. More ROP use in DAWS group. No correlation between DAWS and LD LEDD, LD treatment duration, DA LEDD, cumulative DA LEDD, DA treatment duration, total LEDD, age, sex, PD duration, onset age.		50% of all DA withdrawals due to ICDs. DAWS unresponsive to LD substitution.
A251	Pontieri 2015	Any ICD 37% (PG 14%, ICD-NOS 23%).	With PG and other ICD: younger, younger at PD onset, longer disease duration, higher LD dose, higher DA dose, higher total LEDD. No difference in sex.	PG showed higher depression (HDRS) and anxiety (HARS) scores than both ICD-NOS and No-ICD ($p < 0.005$).<br><br>Both PG & ICD-NOS had greater psychosis burden (PPRS total) vs No-ICD. ICD-NOS particularly elevated in hallucinations, illusions, paranoid ideation, ERS score.<br><br>No group differences in apathy or hedonic tone except trend to greater anhedonia (SHaPS) in PG.<br><br>No significant differences across groups on any cognitive test (executive, memory, attention, language).	
A252	Pontone 2006	ICD prevalence: 9% (CSB, pathologic PG, CB)	DA use and PPX use but not ROP use specifically risk factor for ICD.	Discrete symptoms of depressed mood, irritability, and appetite	

			With ICD: no difference in age, onset age, PD duration, LD LEDD.	changes were more likely to be present and mote severe in the ICD group	
A253	Porta 2016		3/7 experienced ICDs.		Prompt reduction of PPX dose to 0.008-0.017 mg/kg/day in all but one patients allowed the disappearance of ICD.
A254	Pourcher 2010	12.4 % on DA-agonist therapy developed new impulse-control / compulsive behaviours within 1–15 months of treatment.<br><br>Phenotypes (n): BE (4), CB (3), trichotillomania (2), pathologic PG (1), nocturnal food craving (1), new motor/vocal tics (2). Some patients had >1 feature.<br><br>Financial or metabolic consequences noted (credit-card debt, weight gain, abnormal glycaemia, PG losses up to $4 000).	No patients with compulsive behaviour without dopaminergic treatment. With compulsive behaviour: no difference in sex, age at onset, DA dose.	Patients with compulsions had higher depression scores (BDI-II), higher self-reported stress, and more sleep-disturbance (MOS Sleep Scale) than both comparison groups at every time-point ($p < 0.05$).	DAs stopped in three RLS patients because of compulsion exacerbation. Dopaminergic treatment dosage was reduced in 7 patients in order to stop the compulsions.
A255	Rábano-Suárez 2021	Overall ICD prevalence during lockdown: 13 % vs 4.5 % in control period ($p = 0.02$).<br><br>Among patients treated with DA ICD prevalence rose to 31 % vs 9.6 % pre-lockdown ($p = 0.026$).	With ICD: younger, younger onset age. No difference in DA LEDD.	Patients who developed ICD during lockdown more frequently had a history of depression (53 % vs 27 %; $p = 0.041$).	
A256	Rabinak 2010		5/26 with DAWS: all had ICDs, higher DA LEDD pre-withdrawal, higher cumulative DA LEDD. No difference in age, sex, onset age, disease duration, PPX use, total LEDD, DA treatment duration.		DAWS unresponsive to LD substitution, antidepressants, benzodiazepines, and CBT, but rapidly and dramatically with DA repletion.
A257	Rana 2013	Prevalence of ICD in the total cohort: 5.7 %.<br>Types of ICD observed (numb pats): PG/risk-taking (5 pts), CSB (2 pts), BE (2), CB/punding (1). One patient exhibited multiple ICD behaviours.			

A258	Redenšek 2020	Prevalence of ICDs: 15%		Depression emerged as the only comorbidity that independently and meaningfully predicted ICD	
A259	Ren 2023	Prevalence of any ICD: 11.6 % (QUIP-positive and interview).<br><br>PG 2.8 %; CSB 4.9 %; CB 3.9 %; BE 5.3 %.	With ICD: longer disease duration, higher LEDD, trend for DA use; no difference in sex, age, PPX use, amantadine use, selegiline use.	Independent risk correlates:<br><br>- Coffee consumption history (especially for multiple ICDs, PG, CSB)<br>- Anxiety (HAMA ≥ 14)<br>- Apathy (AES ≥ 14; linked to multiple ICDs & BE)<br>-Probable REM-sleep behaviour disorder (RBDSQ ≥ 5)<br>- Worse cognitive scores (MMSE / MoCA) showed positive correlation in univariate table.<br><br>No significant association with depression severity, motor severity (UPDRS-III/H–Y), or dyskinesia after adjustment.	
A260	Ricciardi 2018		PD with ICB: shorter disease duration to FU, lower total LEDD. No difference in age, sex, disease duration to BL, DA LEDD.	Comorbid non-motor symptoms (autonomic dysfunction, mild cognitive impairment, higher anxiety, probable RBD) present at diagnosis were associated with later ICB emergence.<br><br>No associations with motor severity. Imaging did not reveal BL morphological comorbidity markers.	
A261	Ricciardi 2023		With ICD: younger. Trend for higher total LEDD. No difference in sex, disease duration, DA LEDD.	Higher depression, anxiety, and apathy in PD-ICB group.<br><br>No association between presence or severity of ICBs and dyskinesia severity.	
A262	Rieu 2016	Prevalence (ASBPD)<br>-Hypodopaminergic symptoms ≥ 1: 80.7 %.<br>-Non-motor fluctuations: 50.7 % (ON 46 %, OFF 76 %).			

		-Hyper-dopaminergic behaviours ≥ 1: 89 % (19.9 % one behaviour; 38.9 % ≥3).<br><br>Specific ICD/ICRB mean severity scores (0–4): eating 0.60; hobbyism 0.40; compulsive shopping 0.23; PG 0.21; CSB 0.17; etc. Severe ICDs were rare (≤ 2 %).<br><br>Impact on quality of life:<br>-ICD/ICRBs showed modest, selective effects:<br>-CB ncreased emotional-well-being score<br>-BE behaviour worsened cognitive-impairment domain<br>-hobbyism and excess motivation were associated with better mobility or emotional-well-being scores, suggesting a possible hedonic/positive effect when mild.			
A263	Rizos 2016	Overall ICD prevalence 13.4 %<br><br>Rotigotine patch: 4.9 %<br>ROP pooled: 13.9 % , IR 14.0 %, XL 13.9 %.<br><br>PPX pooled: 13.8 %, IR 19.0 %, PR 6.6 %.<br><br>CSB was the leading ICD in ROP users	Lower ICD rate on rotigotine than ROP/PPX with exception of PPX ER. Fewer ICDs on PPX ER than PPX IR.		50.9% with ICDs discontinued DA therapy due to ICDs after a variable period following development of the ICD. Mean dose and mean duration of therapy were similar for ICD patients who discontinued versus those who did not.
A264	Rodríguez-Violante 2014	Any ICD-RD: PD 25.6 % vs controls 16.6 % (p = 0.032).<br><br>ICDs only: PD 10.6 % vs 5.3 % (p = 0.061).<br><br>Hobbyism/punding: PD 14.3 % vs 4.6 % (p = 0.002).	PD with ICD: younger, more DA use, more PPX use, higher DA LEDD. No difference in sex, LD use, total LEDD.		

		Most common ICD: BE (8.6 % PD). PG least frequent (1.3 %).<br><br>4.6 % of PD patients had ≥2 ICDs, none in controls.			
A265	Rodríguez-Violante 2021	At BL 18 % had ≥ 1 ICD by QUIP-RS. ICD spectrum: BE 45.8 %, hobbyism 45.8 %, punding 25 %, compulsive shopping 8.3 %, CSB 4.2 %, PG 0 %.	All ICD patients on DA. With ICD: Higher DA LEDD, higher total LEDD. No difference in onset age, sex.		Strategy most used with ICD: no change, followed by DA removal, DA switch and DA lowering. Unexpectedly, in 20.8% with ICD, the DA dose was increased. Antipsychotics, CBT and DBS strategies were not used.<br><br>No change most effective remission strategy. Even remission in 60% with increased DA dose.
A266	Rossi 2017		With ICD pre-op: Higher pre-op total LEDD. 1/14 with worse ICD post-op, 4/23 with de novo ICD.		No change in DA LEDD or total LEDD post-op. Only 2 pre-ICD patients had significant DA dose reduction post-op.<br><br>ICD resolved in 7/14, about half with and without DA dose reduction.<br><br>Lower QUIP-RS post-op in all patients and those with pre-ICD, both for GPi and STN, but only significant for GPi group.
A267	Saadat 2020	OCD 71.2%	With compulsive behaviour: No difference in sex distribution.		
A268	Sachdeva 2014		With CSB: higher total LEDD. No difference in age, sex, DA LEDD, PD duration. With other ICD: No difference in age, sex, total LEDD, DA LEDD, PD duration.	CSB associated with significantly higher anxiety (HADS-A) vs controls (also true for other ICD group).<br><br>No group differences in depression, cognition (MMSE, executive tests) or motor severity (UPDRS).<br><br>No association with family/personal history of substance abuse, forensic history, marital status or Hoehn-Yahr stage.	

A269	Sahin 2023	20 out of 72 patients had ICD: 8 patients (40%) with CB disorder, 7(35%) with punding, 5(25%) with CSB	With ICD: younger. No difference in sex, disease duration, cabergoline treatment duration, cabergoline dose, cumulative cabergoline dose.		
A270	Sanjan 2023		No difference in any ICD or BIS-11 between the two underlying conditions, one treated with cabergoline and one not.		
A271	Santangelo 2009		With PG: No difference in sex, age, onset age, PD duration, total LEDD, DA LEDD, PPX use, ROP use, LD monotherapy use, DA monotherapy use, LD+DA use.	PG was strongly associated with frontal-executive dysfunction.<br><br>Depression (HAM-D) severity did not differ between groups and was not a predictor of PG	
A272	Santin 2021	BL prevalence of ICDs: 10.6 %	7/194 patients without preoperative ICD developed de novo ICD, associated with higher LD and DA doses, and DA increase vs decrease at FU. No impact of stimulation position or electrical energy used.	BL apathy was associated with higher risk of developing a new ICD after DBS.<br><br>No significant differences in cognition , anxiety or depression between ICD/no-ICD groups after adjusting for medication.	22/23 patients (95.6%) with preoperative ICD improved and 1 remained stable after DBS. Trend for improvemeny being more likely with less motor symptoms/dyskinesia and lower LD and DA doses at follow-up.<br>Following DBS, the LEDD and DA daily dose dropped by 49.1% and 64.66%, respectively.
A273	Santos García 2019	ICD: 12.7 %; CB: 7.2 %.			
A274	Santos-García 2021	Prevalence of ICB: 17 %<br>Any ICD: 12 %<br>Any CB: 9 %.<br><br>QoL worst when depression and ICBs co-existed.	With ICB: younger, longer PD duration, higher total LEDD, higher LD LEDD, more DA use, higher DA LEDD, longer LD treatment duration, more polypharmacy. No difference in sex.	Depression more common with ICBs: 66.3 % vs 47.5 % (p < 0.0001).<br><br>Major depression: 22.1 % with ICBs vs 14.5 % without (p = 0.041).<br><br>Specific links: PG (88.9 % depressed), BE (65.9 %) and hobbyism-punding (69 %) – all significantly higher than in those without the behaviour.<br><br>ICB group showed higher non-motor burden , greater fatigue, sleep disturbance and UPDRS-IV scores.	

A275	Santos-García 2021	Prevalence of ICD:<br>PD without sleep problem: 5.4%<br>PD with sleep problems: 12.8%		Prevalence of ICD was higher in PD with sleep problems.	
A276	Sauerbier 2021	BL prevalence: 38.9 % had clinically relevant ICB by QUIP-RS (mainly punding/hobbyism, eating, shopping, CSB)	Larger change in QUIP-RS total was predicted by higher baseline QUIP-RS total and lower baseline DA LEDD. Out of 55, 15 patients experienced worsening of QUIP-RS total. Associated with worsening: higher baseline DA LEDD, lower baseline QUIP-RS total.	Higher BL attention/memory deficits (NMSS domain 5) predicted postoperative ICB worsening.	In subgroup with preoperative ICB (14/55), the QUIP-RS total improved significantly at 6-months FU. LEDD total and DA LEDD lower at FU both in all patients and those with pre-ICB.<br><br>Out of 55, 16 patients (29.1%) experienced clinically relevant improvement of QUIP-RS total.<br><br>In pre-ICB (14), clinically relevant improvement observed in 6 patients, worsening in 1 patient, stable in 7.
A277	Scavone 2020		56% under PPX use, 15% under ROP, 11% under LD.		
A278	Schreglmann 2012	21 % developed new-onset ICDs after initiating rotigotine. Manifestations (n): CSB (4), BE with marked weight gain (2), CB (2), PG (2), punding (1). Several patients had multiple behaviours.	No ICDs at baseline, 6/28 developed ICD under rotigotine treatment. All male, no difference in age.		ICDs improved after rotigotine discontinuation or dose reduction.
A279	Scott 2020	Prevalence: ≥1 ICD (QUIP-RS): 27.6 %	With ICD vs no ICD/apathy: higher DA LEDD. No difference in sex, age, total LEDD. Male sex predictor for higher QUIP-RS.	Depression (29 %) and anxiety (41 %) were common and positively correlated with ICD.	
A280	Scott 2022	Prevalence:<br>Apathy only (n = 7)<br>ICD only (n = 19)<br>Both apathy + ICD (n = 15)<br>Neither (n = 23).	With ICD: no difference in age, onset age, sex, total LEDD, disease duration.	Patients with both apathy + ICD showed the highest scores on emotion-based impulsivity (positive & negative urgency) and on anhedonia (consummatory), plus greater lack of pre-meditation/perseverance.<br><br>ICD-only patients displayed elevated sensation-seeking but comparatively less anhedonia.	

				For the whole sample, greater ICD severity (QUIP-RS) correlated with higher urgency scores and with depressive/anxiety symptoms.	
A281	Scott 2024	ICD prevalence: 49.5 % scored above threshold on the QUIP-RS. Subtype prevalence – PG 5.2 %, BE 16.5 %, CB 7.2 %, CSB 9.3 %, hobbyism/punding 39.2 %.<br><br>Higher ICD scores were linked to poorer quality of life.		Strong positive correlations between ICD scores and depression (BDI-II), trait anxiety (STAI), anhedonia (SHAPS)	
A282	Scullin 2013		With impulsivity: No difference in PD duration, age, sex, LD use, DA use.	Impulsive PD patients reported significantly more sleep complaints than non-impulsive PD patients (total sleep-complaint composite).<br><br>Largest effects: poor sleep efficiency (greater nocturnal awakenings, more difficulty initiating sleep) and higher daytime sleepiness (Epworth), both $p < 0.01$.<br><br>RLS also more frequent in impulsive group, $p < 0.05$.<br><br>No group difference in total sleep time or snoring/nightmares/nocturia.	
A283	Shapiro 2007	Pathological/compulsive PG prevalence among responders = 7.9 %.<br>PG profile: slots (70 %), scratch-offs (50 %), cards (40 %) most frequent in CGs; 60 % of CGs engaged in >1 PG modality.	With PG: younger, more DA use. No difference in sex, PD duration, LD use, amantadine use, MAOB-I use.	CGs showed higher anxiety, anger and confusion, but Beck Depression Inventory-II scores were not different among groups.<br><br>No significant group differences in personal depression history; however only CGs (10 %) reported personal alcoholism history.<br><br>Smoking: 20 % of CGs were current smokers vs 0 % in other groups ($p<0.001$).	

				"Four As" proposed by authors: Compulsive gamblers tend to be Anger-prone, Anxious, (younger) Age, and on DAs.	
A284	Sharma 2015	Prevalence (QUIP):<br>≥ 1 ICD-RB: 42.8 %.<br>• ≥ 1 classical ICD: 24.8 % (CSB 11 %, compulsive shopping 8.4 %, BE 5.4 %, PG 3.3 %).<br><br>≥ 1 ICD-related compulsive behaviour (ICRB): 31.1 % (punding 12.4 %, hobbyism 9.4 %, walkabout 4 %, compulsive medication use 7.7 %).<br><br>Multiple ICD-RBs (≥ 2) in 15.7 %.	Highest ICB prevalence under LD+DA, then DA monotherapy, then LD monotherapy, higher LD and DA LEDD, longer DA and LD treatment duration.<br><br>With ICB: younger onset age, longer PD duration, more LD use, more DA use, more amantadine use, more entacapone use and higher entacapone dose, less rasagiline use, higher rasagiline dose, higher amantadine dose, longer amantadine treatment duration. More PPX and less ROP use, higher PPX and ROP dose, longer PPX and ROP treatment duration. Higher DA and total LEDD. No difference in age, sex, rasagiline or entacapone treatment duration.<br><br>ICB predictors: longer PD duration, higher LD, DA and total LEDD.		
A285	Simoni 2020	Prevalence of clinically-significant ICRB: 34 %	ICB prevalence and QUIP-RS higher under oral treatment compared to LCIG in dyskinetic patients.		
A286	Şimşek Erdem 2023		Worsening of ICD post-op in 5/8. No de novo ICDs.		Lower post-op total LEDD. 1/8 with pre-op ICD in remission after DA reduction, no change in 2/8.
A287	Singh 2007	Prevalence: 19% reported a new compulsion after starting a DA. 6% of cohort met DSM criteria for PG.	With any ICB: younger, more often male, higher LD LEDD. Trend for more LD use. No difference in DA LEDD. With PG: more often male, younger. No difference in DA type, DA LEDD and DA treatment duration, LD cotreatment, LD LEDD, LD treatment duration, PD duration.		Interventions for PG: Counselling, abstincence programme, DA discontinuation/reduction.

A288	Siri 2010	OCD symptoms 48 % (10 % severe).	OCD behaviour less common under DA treatment than LD treatment. Higher total and LD LEDD. No difference in age, sex, onset age, PD duration, DA LEDD. Severity score correlated with LD LEDD, but not disease duration.		
A289	Siri 2010	Mean PG losses ≈ €2 500 per month (range €500–8 000).	With PG: younger, more often male. Trend for younger onset age. No difference in total LEDD, LD LEDD, DA use, PD duration, DA type.	NPI showed greater aggressiveness, disinhibition, irritability, and eating disorders in PG patients; controls had higher anxiety & apathy.<br><br>PD-PG patients showed intact (even slightly superior) cognitive and executive abilities.	
A290	Siri 2015		With ICD at BL: Trend for higher total LEDD. No difference in sex, onset age, PD duration, LD LEDD, DA LEDD, DA use, MAOB-I use. Same results for these patients at FU.	ICD patients exhibited more neuropsychiatric symptoms (disinhibition, aggression, abnormal eating), but depression and global cognition were comparable to controls.<br><br>Nicotine addiction tended to be more common in ICD group.	Full remission in 16/40, partial in 10/40. Associated with remission: PG as ICD, atypical antipsychotic use, trend for antidepressant use. No association with DA, LD or total LEDD decrease, sex, onset age, PD duration.<br><br>After DA withdrawal: 7/11 in remission, 4 persisted.
A291	Smith 2016	Cumulative incident ICD-symptom rates: 7.8 % (Y1) → 18.1 % (Y2) → 25.1 % (Y3).			
A292	Sohtaoĝlu 2010		With persisting ICD: Higher DA dose at BL. No difference in age, disease duration, LD dose at baseline, LD, DA or total LEDD at FU.		Lower DA LEDD at follow-up, higher LD LEDD. ICD persisted in 6/22, all under antipsychotic treatment. No benefit of quetiapine
A293	Solla 2011	Prevalence of ICDs: 10%<br><br>PG 3.2 %<br>CSB 4.0 %<br>Compulsive shopping 1.1 % (mainly females)<br>BE 2.9 %<br>Repetitive behaviours (punding) 3.3 %	With ICD: younger onset age, higher total LEDD, higher DA LEDD, higher LD LEDD, more DA use. CSB more common in men. No difference in sex, PD duration, LD use.	Any ICDs were more frequent in patients with motor complications (12 % vs 3 %, $p < 0.01$).<br><br>Dyskinesias specifically associated with CSB and CB. All DDS cases had dyskinesias.	

		Dopamine-dysregulation syndrome (DDS) 2.3 %			
A294	Solla 2012	Prevalence of ICD: about 13% in both sexes (13.2 % in men; 13.8 % in women).<br><br>No gender difference for PG, BE, CB.	With ICD: No difference in sex besides CSB (more frequent in men)		
A295	Somma 2022		Mild worsening in BIS-11 at 12-months FU.		
A296	Somme 2014	Prevalence of any ICB: 33 %.<br><br>BE 13 %; punding 11 %; ICD cluster (CB, PG, CSB) 15 % overall (some overlap).	Trend for more ICDs under DA treatment. Patients with DA monotherapy and combination therapy with LD showed similar proportions of ICB. Tendency for less ICBs on rotigotine.<br>With punding: higher DA and LD LEDD, longer disease duration.<br>BE as only ICD not related to dopaminergic therapy.		
A297	Staubo 2024		With ICD: Higher ROP serum concentration and dose, longer treatment duration with ROP, longer disease duration in ROP group. No difference in PPX serum concentration, dose, treatment duration.		
A298	Staubo 2025		Higher total ROP dose and exposure, but not PPX dose and exposure, correlated with higher QUIP-RS.		
A299	Sulu 2023	7.1 % developed a new-onset compulsive PG disorder after treatment start with carbegoline (CBG).<br><br>ICD prevalence in cross sectional arm (already were on treatment): 8.8 %in CBG-treated vs 5.9 %in DA-naïve controls (p = 1.0).	1/14 with PG after cabergoline initiation. No difference in QUIP-RS, BIS-11, MIDI or IGT after cabergoline initiation. With cabergoline vs without: No difference in QUIP-RS, BIS-11, MIDI, IGT.		PG improved after cabergoline dose reduction in 1/1.
A300	Takahashi 2022	Prevalence of ICBs: 16.4 % had ≥1 ICB; 4.4 % had ≥2.<br>Hobbyism 10.1 %, BE 5 %, punding	With ICB: younger onset age, longer PD duration, higher LD LEDD. No		

		3.8 %, CSB 3.8 %, CB 3.1 %, PG 0 %, DDS 3.8 %.	difference in age, sex, DBS use, DA LEDD.		
A301	Tanaka 2013	Prevalence (interview-confirmed):<br>PG 6.5 %<br>CSB 3.2 %<br>CB 3.2 %<br>BE 3.2 %<br>Punding 6.5 %<br>DDS 2.2 %<br><br>≥ 1 ICD (PG/sex/CB/eating) 12.9 %; ≥ 1 ICB (any of the above + punding + DDS) 21.5 %.	With ICB: younger, younger onset age, longer PD duration, higher total and LD LEDD. Trend for higher DA LEDD. No difference in sex, DA use, doses of PPX, ROP, selegiline, amantadine.	Non-motor symptoms (depression, apathy, sleep disorders, RBD, fatigue, orthostatic hypotension, constipation, hallucinations, olfactory loss) not associated with ICB presence.	
A302	Tang 2024	Frequency of ICDs at the end of FU (4y) was 23.8%.<br><br>54.7% with BE, 30.2% with CSB, 13.2% with<br>CB, and 1.9% with pathologic PG.<br><br>The mean duration from dopamine replacement therapy to develop ICDs was 3.30 ± 2.42 years.	With ICD: higher DA LEDD, more DA use. No difference in age, onset age, PD duration, sex distribution, total LEDD. ICD predictor: DA LEDD.	Excessive daytime sleepiness, high depression scores and<br>postural instability were associated with ICDs occurrence.	
A303	Tayim 2024	Impact on patients' lives was predominantly negative: marital strain, reduced emotional closeness, tension with partners, disruption of family and social life, work inefficiency, financial cost (e.g., paying for sex services).<br><br>Partners usually expressed distress, anger, or embarrassment.<br><br>High levels of stigma and shame; patients concealed behaviours from family and clinicians.<br><br>Barriers to help-seeking: embarrassment, fear of judgement, perception that clinicians “never ask”.		Past life factors: childhood sexual abuse in 2 participants, prior alcohol misuse in 1.<br><br>Mood symptoms (depression, loneliness) frequently triggered CSB episodes.	

		Patient aspirations: desire for clearer information, routine screening and open dialogue with health professionals about sexual side-effects.<br><br>Co-existing ICDs reported in several participants: BE (4), CB (3), compulsive PG (1).			
A304	Tayim 2025	Impact on carers included:<br>– emotional burden: distress, humiliation, anxiety, anger, sleep loss;<br>– relationship strain: loss of intimacy, fear of abuse, marital conflict, concerns about fidelity and sexually transmitted infections;<br>– social & stigma: secrecy, embarrassment, avoidance of social activities, perceived judgement from peers/clinicians;<br>– mental-health consequences: low mood, altered self-image, feelings of rejection, carer burnout.<br><br>In 4/5 PD cases, reducing or discontinuing the implicated DA led to some improvement, but urges often persisted.<br><br>Patients were not adequately informed about the likelihood and implications of CSB when taking drugs for PD (DA).			
A305	Terenzi 2018		With BE: no difference in disease duration, DA/LD/total LEDD.	With BE patients had significantly higher BMI than PD controls and HC, higher depression scores, and poorer working-memory / executive scores.	

A306	Thomas 2010				In all patients, attempts to reduce/switch DA to LD or change DA, gambling counseling, and behavioral strategies had been unsuccessful.<br><br>Robust PG improvement in all patients early on in amantadine treatment vs placebo.
A307	Thomson 2021	Impact on caregivers included distress, relationship strain, need for counselling.<br><br>Emphasised need for pre-operative counselling, informed consent about possible ICD-like changes, family education, and systematic post-operative monitoring/management.	A pool of common experiences after DBS included increased impulsivity (n=7) and impaired decision-making (n=3).		
A308	Todorova 2015		With apomorphine: 7 de novo ICD cases of which 4 resolved later on and 1 led to apomorphine discontinuation.<br><br>With LCIG: No de novo ICDs.		With apomorphine infusion: 1/4 ICDs resolved, 3/4 improved.<br><br>With LCIG: 6/8 ICDs resolved, 2/8 improved.
A309	Tolosa 2021	44.9 % of PD respondents reported ≥1 ICB.<br><br>Impact on patients: high non-motor burden, reduced HRQoL. Carers: mild–moderate burden. ICBs are recognised contributors to distress but specific burden scores by presence/absence of ICBs were not presented.	ICB more common under any DA use (past/present) and most common under present DA use.		
A310	Toś 2023	Prevalence of ICDs: 27.4%<br>BE: 12.6%<br>CSB: 11.1%<br>CB: 10.4%<br>PG: 5.2%<br><br>8.9% had ≥2 ICDs.	With ICD: Longer disease duration, more DA use, higher DA LEDD. No difference in sex, age, age at diagnosis, LD LEDD, LD use.<br>CSB and hobbyism more common in men.<br>No difference between PPX and ROP	Sleep disorders, longer disease duration, and motor complications were associated with increased ICD risk	

		ICD-RBs were present in 26.7% (hobbyism 20, punding 27, walkabout 8).<br><br>Only 13.5% of ICD patients were identified in routine medical history.	use. No ICDs under apomorphine infusion or rotigotine patch. Higher risk under DA+LD than only DA.<br>No effect of other dopaminergics or DBS.		
A311	Toś 2024	Prevalence of ICD: PD 24.7 %; PSP 18.5 %; MSA 11.1 %.<br>BE 11.5 %, CB 10.4 %, CSB 8.8 %, PG 4.4 %.<br>7.7 % had ≥2 ICDs.<br><br>PSP: CSB 11 %, CB 7 %, BE 3.7 %.	CSB and multiple ICDs occurred more often in men than in women. PD with ICD: Longer disease duration, more DA use, higher total, LD and DA LEDD. No difference in age, age at diagnosis, sex, other dopaminergics or DBS.	PD patients with ICDs had more motor complications and more sleep disorders. No differences in cognition or depression severity.	
A312	Trovini 2025		No change in BIS-11 from BL to 18 months FU in total population (aripiprazole or brexpiprazole treatment). No different effect on BIS-11 by aripiprazole or brexpiprazole.		
A313	Turcano 2024	ICD prevalence among DA users: 14.5 %; none occurred in non-exposed patients.<br><br>Most frequent ICD subtype: CSB 38.3 %, PG 25 %, CB 22 %, BE 20 %.	With ICD: DA monotherapy, DBS, trend for younger PD onset. CSB more frequent in men.	Subsequent dementia was more common in ICD + patients (13.3 % vs 3.0 %, p < 0.001)	ICDs resolved in 40/60 patients after decreasing or discontinuing DA
A314	Umemura 2011		Within 3 months after surgery: 2 transient ICD-AEs.		2/2 improved with drastic reduction of dopaminergic medication within a few weeks.
A315	Valença 2013	Prevalence (QUIP-S): at least one ICD in 18.4 % of PD patients vs 4.2 % of controls (p < 0.001).<br><br>PG 1.3 %, CSB 11.8 %, CB 10.5 %, BE 7.9 %.	With ICD: younger, less LD use, more PPX use, higher PPX dose. Trend for more often male. No difference in amantadine use, selegiline use, total LEDD, PD duration. Higher PPX dose predictor for ICDs.		
A316	Vargas 2019	Prevalence of current ICDs (≥1):<br>PD overall 16.9 %<br>Controls 15.2 % (non-sign diff)<br><br>PD + DA 27.8 % vs PD no-DA 10.2 % (p = 0.001) and vs controls (p =	DA users younger and with higher ICD frequency. PD vs controls: PG, CSB and punding more common.		

		0.014).<br><br>Punding: PD 5.3 % vs controls 1.3 % (p = 0.028).<br><br>Individual ICD frequencies (PD vs control):<br>CB 7.7 % vs 9.6 %.<br>BE 6.8 % vs 6.5 %.<br>PG disorder 4.8 % vs 1.3 % (p = 0.027). CSB 3.9 % vs 0.4 % (p = 0.015).			
A317	Varshney 2024	ICD prevalence:<br>-PG = 1 case<br>-BE/craving = 1 case<br>-punding = 1 case<br><br>Overall ICD frequency 9.4 % (3/32).			
A318	Veiga 2025	ICB prevalence: 52.2 %	With ICB: longer disease duration, higher total LEDD, more DA use. No difference in age, sex, DA LEDD. ICB predictors: younger disease onset, trend for male sex and DA use.	History of impulsivity pre-PD and higher depression score strongly elevated risk for ICD.	
A319	Vela 2016	Any ICB: 58.3 % in PD vs 32.9 % in HC<br><br>Multiple ICBs (≥2): 34.5 % PD vs 12.9 % HC.<br>Specific behaviours (% PD vs HC, p):<br>PG 10.7 vs 2.4 %<br>CSB 23.8 vs 10.6 %<br>CB 15.5 vs 0 %<br>BE 20.2 vs 10.6 %<br>punding 17.9 vs 5.9 % .<br><br>Hobbyism most common overall (29.8 % PD, 20 % HC)<br><br>Quality of life: PDQ-39 worse in ICB patients (median 40 vs 20, p = 0.021) but effect mediated by depression.	With ICB: More DA use. No difference in age, sex, LD use, rasagiline use, LD LEDD, total LEDD. Trend for higher DA LEDD dose, longer disease duration.<br>CSB more common in men and trend towards more BE in women. No differences between different DAs or type of release form. DA use predicts ICB.	ICBs were associated with depression.	

A320	Verbaan 2009	19% of all patients reported compulsive symptoms	With ICB: younger onset age, more often male, higher DA LEDD. No difference in age, PD duration, total and LD LEDD.	No correlation between psychotic and compulsive symptom scores.	
A321	Vitale 2011		With ICD: no difference in age, onset age, PD duration, total LEDD, DA LEDD.	All ICD groups performed worse than PD controls on spatial-planning (ROCF copy) and set-shifting (TMT B–A) tasks – indicating frontal-executive deficits.<br><br>CSB and multiple-ICD groups showed additional impairment on Stroop interference (poorer inhibitory control) and on verbal learning & delayed recall versus controls and pathological gamblers.<br><br>BE showed intermediate deficits (verbal memory & planning) but less severe than HS/mICDs.<br><br>PG associated only with executive (frontal) deficits.<br><br>No group differences in HAMD-17 or HADS anxiety/depression scores.	
A322	Voon 2006	PG: 3.4%, Pathologic CSB: 2.4%, Compulsive shopping: 0.7%.	With ICD: more often under DA monotherapy than LD monotherapy. No differences between DA types.<br>With CSB: more often male, younger, younger onset age, higher total LEDD. No difference in DA LEDD, LD use, DA use.<br>With CB: younger, younger onset age.<br>With PG: no differences.	CSB group: 6/7 had current major depression. Other findings included hypomania, panic disorder, subsyndromal OCD.<br><br>CB group: both cases had depression.<br><br>PG group: mix of hypomania, anxiety, depression in minority.	
A323	Voon 2006	Lifetime prevalence of PG: 3.4%.<br><br>3-month active PG prevalence: 1.7%<br><br>Lifetime PG prevalence among those on any DA: 7.2%	With PG: younger onset age, more DA use. No difference in sex, age, total LEDD, DA LEDD. No difference between DA monotherapy or combined with LD. No difference between PPX, ROP or pergolide.		

A324	Voon 2007		With PG: younger, younger onset age, more often under LD+DA. Trend for higher DA LEDD. No difference in total LEDD, sex, PD duration, DA monotherapy, DA type. PG predictor: onset age.	Strongest predictors for PG were high novelty-seeking temperament, personal or familial alcohol-use disorder, and early-onset PD.	
A325	Voon 2010			DA increased impulsive choice in PD patients with ICDs but not in PD-controls.<br><br>Patients with ICDs showed faster reaction times and reduced decision-conflict RT.<br><br>PD patients with ICDs displayed greater spatial-working-memory errors than PD-controls.	
A326	Voon 2011		With ICD: higher LD and total LEDD.	ICD patients had greater functional impairment (UPDRS-II) and lower motivation scores, higher depression (GDS), state & trait anxiety (STAI), and OCD symptoms (OCI-R).<br><br>Each single-ICD subgroup (PG, shopping, sexual, eating) vs controls: all shared higher depression & BIS. PG & shopping showed higher novelty-seeking; shopping showed highest choice-impulsivity.<br><br>Multiple-ICD patients (n = 81) had higher dyskinesia scores than single-ICD patients.<br><br>ICD patients more likely to be current/ex-smokers.	
A327	Voon 2011	Prevalence of ICD:<br>- any ICD 7.1 %<br>-BE 4.3 %<br>-CB 3.6 %	With ICB: more often female, higher DA LEDD, younger onset age. No difference in DA type. ICB predictor: DA LEDD.	History of experimental drug use and family history of PG disorder predicted ICD presence.	

		-PG 2.1 % (all Internet PG)<br>-punding 2.1 %<br>-CSB 1.4 %			
A328	Wang 2016	Prevalence of ICD behaviours (confirmed by specialist interview after modified MIDI screen):<br>-any ICD 4.15 %<br>-CSB 1.84 %<br>-PG 1.38<br>– CB 0.46 %<br>– BE 0.46 %	With ICD: higher DA and total LEDD. Trend for higher LD LEDD and more DA use. No difference in age, sex, PD duration.		
A329	Waskowiak 2022	45 % developed an ICD over mean 35 months	ICD predictors: DA use, male sex.	Higher trait anxiety at diagnosis independently increased the prospective risk of developing ICDs, whereas BL depression did not.	
A330	Weintraub 2006		With ICD: younger, longer disease duration, higher total LEDD, more DA use, more amantadine use, higher DA LEDD. No difference in sex, LD LEDD.<br>DA use was ICD predictor.<br>No differences between PPX, ROP, pergolide. ICDs associated with higher pergolide dose but not PPX or ROP dose.		7/18 became asymptomatic either with discontinuation of DA treatment (n=4), DA dose reduction (n=2), or counseling (n=1).
A331	Weintraub 2010	Any ICD: 17.6 % with amantadine vs 12.4 % without (p < 0.001; OR 1.49, 95 % CI 1.19–1.87).<br><br>PG: 7.4 % vs 4.2 % (p < 0.001; OR 1.78).<br>CSB: 5.1 % vs 3.0 % (p = 0.001; OR 1.70).<br>CB: 8.0 % vs 5.0 % (p = 0.005; OR 1.60).<br>BE: 4.4 % vs 4.2 % (ns).	With ICD: more amantadine use, younger age, DA use, higher LD LEDD.		
A332	Weintraub 2010	Prevalence of ≥1 ICD: 13.6 %.<br><br>Problem/PG 5.0 %<br>CB 5.7 %<br>BE disorder 4.3 %	With ICD: younger, more DA use, more LD+DA use. No difference in sex, PD duration, DBS, DA LEDD. No difference between PPX and ROP.	Smoking and family PG history independently elevated ICD risk.	

		CSB 3.5 %<br><br>ICD prevalence with DA vs without DA: 17.1 % vs 6.9 %	CSB more common in men, BE and CB in women.<br>ICD predictor: younger age, DA use, LD use, higher LD LEDD.		
A333	Weintraub 2013	ICD or related behaviour symptoms: 18,5%<br>PG: 1.2%<br>CB: 3%<br>CSB: 4.2%<br>BE: 7.1%<br>Punding: 4.8%<br>Hobbyism: 5.4%<br>Walk-about: 0.6%	With ICB: no difference in sex, age.	Increasing severity of depression was associated with presence of any ICDs	
A334	Wicks 2009		13% PG in PD, 3% in ALS and higher gambling score in PD. No correlation of PG with type of dopaminergic drug in PD.<br>Only 2 ALS patients on dopaminergics experimentally (PPX), none of them with PG.		
A335	Wirth 2024	At FU, 54 % of BL-ICD + still had ICD. 21 % of BL-ICD- had developed new ICD.<br><br>Documented consequences among ICD +: casino bans, cash-withdrawal limits, bankruptcies, divorce, convictions, weight gain.	With ICD: lower DA LEDD at baseline. No difference in LD and total LEDD.	At BL,<br>psychiatric disorders were more common among BL-ICD+(42.3 vs 12.3% among BL-ICD-, p<0.01)<br><br>PD-ICD + more frequently experienced<br>akinesia and rigidity worsening .<br><br>At FU, the prevalence of cognitive decline was lower among BL-ICD+.	Strong decrease in DA dose or discontinuation of DA in cases at follow-up. DA LEDD decreased in both cases and controls, LD LEDD only in cases. No predictors of remission identified in treatment strategy.
A336	Wolfschlag 2021	GD diagnosis in 69.8 % of dopamine-agonist users vs 38.7 % of users of other dopaminergic drugs.<br><br>Among patients with psychotic disorders, GD present in 88.8 % of aripiprazole users vs 71.2 % of non-users.	PPX/ROP use vs other dopaminergics and aripiprazole vs other psychotic patients are risk factors for PG.		

A337	Wolfschlag 2024	29 patients with GD.<br>27 patients with other ICDs (some overlap with GD group).	With PG/other ICD: more men, younger onset age, higher DA use before ICD diagnosis.<br>With PG: higher MAOB-I use before diagnosis.<br>No difference in other dopaminergics to control group, no difference in combined therapy DA+LD or MAOB-I+LD.<br>ROP more likely to be prescribed before ICD than PPX.	Psychiatric comorbidities prior to ICD diagnosis in GD group:<br>- alcohol use disorder (10.3% vs 1.6% in controls, p = 0.011).<br>-mood disorders (27.6% vs 12.3%, p = 0.021).<br>-anxiety disorders (24.1% vs 9.2%, p = 0.014).<br><br>Other ICD group:<br>-substance use (non-alcohol) (14.8% vs 1.6%, p < 0.001).<br>-mood disorders (22.2% vs 12.3%, p = 0.041).<br>-anxiety disorders (44.4% vs 9.2%, p < 0.001).<br><br>Post-ICD diagnosis:<br>increased prescriptions of antipsychotics, antidepressants, and mood/anxiety diagnoses (especially in GD group).<br><br><br>Drugs used for addiction treatment (nicotine, buprenorphine, naltrexone) were more frequent in GD group before diagnosis (p = 0.004).	

A338	Wolfschlag 2025	Only 49 % of questionnaire-respondents had ever told their PD clinician about the ICD. Merely 14 % said clinicians pro-actively asked.<br><br>Most prevalent ICD phenotypes (questionnaire):CSB, CB, BE, punding, hobbyism, PG. Two-thirds reported ≥ 1 ICD subtype.<br><br>Impact on patients/relatives:average impact rated mild–moderate overall, but a subset reported severe effects on close relationships, family life and mental health. Finances and employment were less affected.<br><br>Only 12 % (records) / 13 % (questionnaire) had any contact with psychiatry specifically for ICDs, despite higher ICD severity in that subgroup.	ICD severity not influenced by the patient's age or sex.	Psychiatric/cognitive symptoms frequently co-occurred: sleep disturbance 63 %, vivid dreams 56 %, apathy/lack of motivation 56 %, depression 30 %, anxiety 30 %, hallucinations 33 %, mental exhaustion 33 %.<br><br>Substance-related comorbidity was less common: alcohol use disorder 10 %, other SUD 2 %.<br><br>Family history: alcohol use disorder 25 %, ICD/ICB 18 %.	In most cases, ICDs handled exclusively within clinical PD. Only few patients with severe behavioral symptoms and multiple ICDs had been in contact with psychiatric care, either referred by their PD care or on own initiative.<br><br>In two thirds of all patients, the medication suspected for causing the ICD was reduced in dose (44%) or discontinued (22%). Other strategies: change from IR to depot. The majority of adjustments were made to DA treatment.<br><br>Two thirds of the patients that initiated DBS treatment had experienced an improvement of their ICD after the procedure (67%, 6/9) and none reported a worsening of the ICD.
A339	Wu 2014	PD patients with ICDs exhibit significantly greater obsessive-compulsive Internet use than PD patients without ICDs or HCs. Excessive Internet activity aligns with the content of the individual's ICD (e.g., online PG, pornography).			
A340	Wu 2024	Incidence of ICD: 13.8%PG 34.8 %, compulsive shopping 33.3 %, punding/hobbyism next; 36 % had ≥ 2 subtypes.	Highest ICD rates under PPX and ROP, lower under rotigotine and bromocriptine. ICD risk factors: male sex, younger onset age.		In 66/69 ICD resolved or improved with managements. 17/69 could be kept on same DA dose. No DAWS.
A341	Xu 2021	ICRD prevalence (diagnosis confirmed by interview: 9.7 %. BE 3.9 %, DDS 2.9 %, CSB 1.9 %, hobbyism 1.9 %, CB 1.0 %, punding 0.5 %, walkabout 0.5 %, PG 0.5 %. 2.9 % had ≥ 2 ICRDs.	With ICB: longer disease duration, higher total/LD/DA LEDD, trend for younger onset age. No difference in sex, age, DA use, LD use, MAOB-I LEDD, amantadine LEDD.	Depression and heavier overall non-motor symptom burden associated with presence of ICRDs.<br><br>No significant association with anxiety or dyskinesia in this sample.	

A342	Ye 2024	BL prevalence of ICB: 24.9 %.	With ICB: younger, longer disease duration, higher total LEDD. No difference in sex.	Excessive daytime sleepiness and probable RBD were associated with higher ods of developing ICB.<br><br>ICB-positive patients showed more severe depressive and anxiety symptoms.	
A343	Yildirim 2020		Higher QUIP-RS total asociated with longer disease duration, younger onset age, and higher DA dose.		
A344	Zahodne 2011	Prevalence (psychometric, not DSM):<br>– BE Disorder (BED): 1%<br>– Sub-threshold BE: 8.3%<br>– Other ICBs: problem PG 17.8 %; CB 11.5 %; hoarding 8.3 %; CSB 1 %; mania 1 %.<br><br>Co-occurrence: 67 % of overeaters vs 29 % of non-overeaters met criteria for ≥1 additional ICD (χ^2 = 5.37, p = 0.02).	With BE: Trend for more DBS. No difference in sex, age, onset age, PD duration, total LEDD, DA use.		
A345	Zazu 2021	Cariprazine cases: CSB (3), CB (3), BE (3). Diagnoses: mostly schizo-affective disorder.<br><br>Brexpiprazole cases: PG (6), CB (2), CSB (1). Diagnoses: mainly major depression.	Cariprazine and brexpiprazole associated with ICDs		
A346	Zhang 2017	Prevalence of any ICRD (QUIP screen): 31 %<br><br>Most frequent subtypes: compulsive medication use 11.3 %, punding 9.2 %, PG 7.0 %, hobbyism 6.3 %, BE 5.6 %, CB 4.9 %, CSB 2.8 %, walkabout 1.4 %.			
A347	Zhang 2024	BL prevalence (PD): ICBs 20.6 %; apathy 16.8 %; co-occurrence 26.4 % of those with ICBs.	With ICB compared to no ICB/apathy: no difference in age, sex, onset age, disease duration, education, first total LEDD. No	Depression, trait anxiety, autonomic dysfunction and daytime sleepiness were the dominant comorbidities that clustered with ICDs.	

			group differences in total LEDD or DA use at 5 year FU.	RBD was frequent in ICB-positive patients but not specific to the co-occurrence phenotype.	
A348	Zhong 2016	Prevalence: 60 % of the PD sample screened positive for at least one impulsive/compulsive behaviour on the QUIP (sub- or full-syndrome).<br><br>Neither total QUIP score nor the presence of any ICB correlated with caregiver burden as measured by the Parkinson's Disease Caregiver Burden (PDCB) scale (Spearman ρ = 0.238, $p = 0.10$).			
A349	Zhu 2023	BL ICD prevalence: PD 20.7 % vs HC 19.5 % (ns).<br><br>5-year prevalence change: PD increased to 27.3 % ($p < 0.001$); HC decreased to 15.2 %.<br><br>Specific behaviours rising in PD over time: PG, CSB, CB, BE, hobbyism, punding. DDS also rose.	Increase in all ICDs in PD patients over time. PD with ICB: no difference in sex, age, onset age, disease duration. Higher total LEDD at 3 year FU only, higher MAOB-I use at 2 and 3 year FU, higher DA use at year 3, 4, 5. No difference in DA LEDD or any other dopaminergics use at any time point.	CB+ PD patients showed more depression, anxiety, greater autonomic dysfunction and higher excessive daytime sleepiness.	
